# Vitrectomy, subretinal Tissue plasminogen activator and Intravitreal Gas for submacular haemorrhage secondary to Exudative Age-Related macular degeneration (TIGER): study protocol for a phase 3, pan-European, two-group, non-commercial, active-control, observer-masked, superiority, randomised controlled surgical trial

**DOI:** 10.1186/s13063-021-05966-3

**Published:** 2022-01-31

**Authors:** Timothy L. Jackson, Catey Bunce, Riti Desai, Jost Hillenkamp, Chan Ning Lee, Noemi Lois, Tunde Peto, Barnaby C. Reeves, David H. Steel, Rhiannon T. Edwards, Jan C. van Meurs, Hatem Wafa, Yanzhong Wang

**Affiliations:** 1 Faculty of Life Sciences and Medicine, King’s College London, London, UK; 2 The Royal Marsden NHS Foundation Trust, London and Surrey, UK; 3 Department of Ophthalmology, King’s College Hospital NHS Foundation Trust, London, UK; 4 Department of Ophthalmology, University of Wurzburg, Wurzburg, Germany; 5 Wellcome-Wolfson Institute for Experimental Medicine, Queen’s University, Belfast, Northern Ireland; 6 Network of Ophthalmic Reading Centres UK, Queen’s University of Belfast, Belfast, Northern Ireland; 7 University of Bristol, Bristol, UK; 8 Bioscience Institute, Newcastle University, Newcastle, UK; 9 Centre for Health Economics and Medicines Evaluation, Bangor University, Bangor, Wales; 10 Rotterdam Eye Hospital and Erasmus Medical Centre, Rotterdam, the Netherlands; 11 Population Health Sciences, Faculty of Life Sciences and Medicine, King’s College London, London, UK

**Keywords:** Neovascular age-related macular degeneration, Submacular haemorrhage, Alteplase, Tissue plasminogen activator, Pars plana vitrectomy, Gas tamponade, Surgery, Randomised controlled trial, Aflibercept, Anti-vascular endothelial growth factor (anti-VEGF), Economic analysis, Cost-effectiveness, Quality-adjusted life year (QALY)

## Abstract

**Background:**

Neovascular (wet) age-related macular degeneration (AMD) can be associated with large submacular haemorrhage (SMH). The natural history of SMH is very poor, with typically marked and permanent loss of central vision in the affected eye. Practice surveys indicate varied management approaches including observation, intravitreal anti-vascular endothelial growth factor therapy, intravitreal gas to pneumatically displace SMH, intravitreal alteplase (tissue plasminogen activator, TPA) to dissolve the clot, subretinal TPA via vitrectomy, and varying combinations thereof. No large, published, randomised controlled trials have compared these management options.

**Methods:**

TIGER is a phase 3, pan-European, two-group, active-control, observer-masked, superiority, randomised controlled surgical trial. Eligible participants have large, fovea-involving SMH of no more than 15 days duration due to treatment-naïve or previously treated neovascular AMD, including idiopathic polypoidal choroidal vasculopathy and retinal angiomatous proliferation. A total of 210 participants are randomised in a 1:1 ratio to pars plana vitrectomy, off-label subretinal TPA up to 25 μg in 0.25 ml, intravitreal 20% sulfahexafluoride gas and intravitreal aflibercept, or intravitreal aflibercept monotherapy. Aflibercept 2 mg is administered to both groups monthly for 3 doses, then 2-monthly to month 12. The primary efficacy outcome is the proportion of participants with best-corrected visual acuity (BCVA) gain of ≥ 10 Early Treatment Diabetic Retinopathy (ETDRS) letters in the study eye at month 12. Secondary efficacy outcomes (at 6 and 12 months unless noted otherwise) are proportion of participants with a BCVA gain of ≥ 10 ETDRS letters at 6 months, mean ETDRS BCVA, Radner maximum reading speed, National Eye Institute 25-item Visual Function Questionnaire composite score, EQ-5D-5L with vision bolt-on score, Short Warwick and Edinburgh Mental Wellbeing score, scotoma size on Humphrey field analyser, and presence/absence of subfoveal fibrosis and/or atrophy and area of fibrosis/atrophy using independent reading centre multimodal image analysis (12 months only). Key safety outcomes are adverse events, serious adverse events, and important medical events, coded using the Medical Dictionary for Regulatory Activities Preferred Terms.

**Discussion:**

The best management of SMH is unknown. TIGER aims to establish if the benefits of SMH surgery outweigh the risks, relative to aflibercept monotherapy.

**Trial registration:**

ClinicalTrials.govNCT04663750; EudraCT: 2020-004917-10.

**Supplementary Information:**

The online version contains supplementary material available at 10.1186/s13063-021-05966-3.

## Administrative information

The numbers in curly brackets in this protocol refer to SPIRIT checklist item numbers. The order of the items has been modified to group similar items (see http://www.equator-network.org/reporting-guidelines/spirit-2013-statement-defining-standard-protocol-items-for-clinical-trials/). A full SPIRIT checklist is available as a supplementary appendix (Appendix 11).

**Table:** 

**Title {1}**	Vitrectomy, subretinal Tissue plasminogen activator and Intravitreal Gas for submacular haemorrhage secondary to Exudative Age-Related macular degeneration (TIGER): a phase 3, pan-European, two-group, non-commercial, active-control, observer-masked, superiority, randomised controlled surgical trial.
**Trial registration {2a and 2b}.**	Clinical.Trials.gov identifier: NCT04663750, registered 11th December 2020; EudraCT identifier: 2020-004917-10, registered 12th October 2020. All items from the World Health Organisation (WHO) Trial Registration Dataset are available as a supplementary appendix (Appendix 12).
**Protocol version {3}**	Version 1.3, 19^th^ January 2021
**Funding {4}**	EURETINA sought to facilitate a study of vitrectomy, TPA and gas for submacular haemorrhage secondary to neovascular AMD. It commissioned Fight for Sight to establish a pan-European competition seeking bids to run the study, and to administer the award. King’s College London was awarded the research grant from Fight for Sight. To help facilitate set-up prior to the main grant commencing, EURETINA provided King’s College London a smaller start-up research grant. Different prospective sites were known to use different intravitreal drugs to treat wet AMD, and therefore Bayer was approached and agreed to provide and distribute free aflibercept to sites that required it, to standardise background treatment. In the UK, sites are additionally supported by the National Institute for Health Research (NIHR) through its Clinical Research Network. A copy of the funding letter of support is included in Appendix 10.
**Author details {5a}** (Names and Affiliations of Protocol Contributors)	Professor Timothy L. Jackson, Faculty of Life Sciences and Medicine, King’s College London, London, UK Dr Catey Bunce, The Royal Marsden NHS Foundation Trust, London and Surrey UK Mrs Riti Desai, Department of Ophthalmology, King’s College Hospital NHS Foundation Trust, London, UK Professor Jost Hillenkamp, Department of Ophthalmology, University of Wurzburg, Wurzburg, Germany Dr Chan Ning Lee, Department of Ophthalmology, King’s College Hospital NHS Foundation Trust, London, UK Professor Noemi Lois, Wellcome-Wolfson Institute for Experimental Medicine, Queen’s University, Belfast, Northern Ireland Professor Tunde Peto, Network of Ophthalmic Reading Centres UK, Queen’s University of Belfast, Belfast, Northern Ireland Professor Barnaby C. Reeves, University of Bristol, Bristol, UK Professor David H. Steel, Bioscience Institute, Newcastle University, Newcastle, UK Professor Rhiannon T. Edwards, Centre for Health Economics and Medicines Evaluation, Bangor University, Bangor, Wales Dr Hatem Wafa, Population Health Sciences, Faculty of Life Sciences and Medicine, King’s College London, London, UK Dr Yanzhong Wang, Population Health Sciences, Faculty of Life Sciences and Medicine, King’s College London, London, UK
**Name and contact information for the trial sponsor {5b}**	Professor Timothy L. Jackson, Department of Ophthalmology, King’s College Hospital, London SE5 9RS, UK
**Role of sponsor {5c}**	The Sponsor was responsible for the study design. Fight for Sight ran the competition for funding, and as part of the review process its anonymised experts provided feedback on trial design that was incorporated into the final design. EURETINA’s brief to Fight for Sight defined the two groups to be compared (surgery and anti-VEGF therapy versus anti-VEGF monotherapy). The study team (employed by the Sponsor) will be responsible for the collection, management, analysis, and interpretation of data; writing of this and subsequent reports; and the decision to submit all reports for publication. Bayer has no input into any of the design, execution or reporting of the trial, other than provision of free Eylea® to sites that request it.

## Introduction

### Background and rationale {6a}

In high-income countries, exudative and non-exudative AMD together cause more blindness than all other eye diseases combined [1]. Exudative AMD, also called wet or neovascular AMD, can sometimes be associated with a large submacular haemorrhage (SMH). Whilst exudative AMD is a very common disease, an associated large SMH is not. A population-based study in two UK centres found that SMHs larger than 1 disc diameter across occur in 24 people per million per year, with a Scottish Ophthalmic Surveillance Unit (SOSU) study reporting that SMHs larger than 2 disc diameters occur in only 5.4 people per million per year [2, 3]. These data suggest a prevalence rate that would meet the European Commission’s definition of a rare disease [4, 5].

Untreated, SMH typically leads to permanent and severe loss of vision, ranging from 6/30 to light perception [5]. The control group of the submacular surgery trial reported that only 11% of eyes had a final best-corrected visual acuity (BCVA) better than 6/60 [6]. Preclinical studies suggest loss of vision occurs because subfoveal blood leads to rapid photoreceptor damage due to iron-catalysed free radicals via the Fenton reaction, mechanical fibrin contraction, and reduced oxygen and nutrient flux [7]. The end-result is usually a large fibrotic macular scar (38%), atrophy (25%), or retinal pigment epithelium (RPE) tear (22%), with a resulting central scotoma [5].

There are no large, published randomised controlled trials (RCTs) evaluating treatments of SMH. The registration RCTs testing anti-vascular endothelial growth factor (VEGF) drugs for exudative AMD specifically excluded this group. In 2016, we published a systematic review and quantitative synthesis of the SMH literature, containing 159 references identified using PubMed Medline, EMBASE and Cochrane Library Databases [7]. This revealed no RCTs. The literature confirmed the poor natural history of SMH and identified three main treatment strategies:

1. Intravitreal gas tamponade (to mechanically displace the haemorrhage) and tissue plasminogen activator (TPA, to dissolve the clot), with or without anti-VEGF therapy (to control the underlying disease)
2. Vitrectomy, subretinal TPA injection and gas, with or without anti-VEGF therapy
3. Anti-VEGF monotherapy

Since our review, there has been one published RCT. This exploratory study (*N* = 24) compared intravitreal TPA, gas and bevacizumab versus vitrectomy, subretinal TPA, gas and aflibercept. It lacked power to detect any statistically significant difference and concluded that larger studies are needed to determine the best treatment approach [8].

We have undertaken a multicentre, factorial, pilot RCT investigating intravitreal gas, intravitreal TPA and intravitreal ranibizumab (TAPAS; ClinicalTrials.gov Identifier: NCT01835067). Recruitment to TAPAS (*n* = 55) is complete and results are in preparation.

A French RCT (*n* = 90) compares vitrectomy, air, TPA and anti-VEGF therapy versus intravitreal gas, TPA and anti-VEGF therapy (ClinicalTrials.gov Identifier: NCT02557451). Results of the STAR study are awaited.

Due to differences in case mix, none of the different case series can be directly compared, and likewise none of the emerging RCTs are large enough to definitively determine the best management option. An appropriately powered RCT is therefore needed to determine how best to treat SMH secondary to exudative AMD. Methodologically, we favour a study design that compares what we believe to be the best new treatment with standard of care.

### Objectives {7}

We aim to test the hypothesis that vitrectomy, subretinal TPA, intravitreal sulfahexafluoride (SF_6_) gas tamponade and aflibercept are superior to aflibercept monotherapy, with respect to BCVA.

### Trial design {8}

TIGER is a phase 3, pan-European, two-group, 1:1 allocated, active-control, observer-masked, superiority, non-commercial, randomised controlled surgical trial. TPA (alteplase, Actilyse®, Boehringer-Ingelheim) is used off-label, and therefore, from a regulatory perspective, TIGER is a Clinical Trial of an Investigational Medicinal Product (CTIMP). Aflibercept is given accordingly to its marketing authorisation in both arms of the trial, and it is considered standard of care background treatment. Hence, aflibercept is not considered an IMP.

## Methods: participants, interventions and outcomes

### Study setting {9}

Participants will be recruited from vitreoretinal centres across Europe, predominantly from those based in academic hospitals. Potential countries include Austria, Belgium, Bulgaria, Denmark, France, Germany, Hungary, Ireland, Italy, Luxemburg, The Netherlands, Poland, Portugal, Slovenia, Spain, Switzerland and the UK. As countries and sites are selected, details will be available at: www.tigerstudy.org.uk.

### Eligibility criteria {10}

Eligibility will be assessed by study ophthalmologists based on the following criteria:

#### Inclusion criteria

##### General

1. Males or females aged at least 50 years

##### Study eye

2. SMH, comprising sub-neuroretinal haemorrhage with or without sub-RPE haemorrhage, that occurs secondary to treatment-naïve, or previously treated exudative AMD, including choroidal neovascularisation (CNV), idiopathic polypoidal choroidal vasculopathy (IPCV) and retinal angiomatous proliferation (RAP).
3. SMH involving the foveal centre that measures at least 1 disc diameter in greatest linear dimension.
4. Sub-neuroretinal haemorrhage at least 125 microns thick, measured at the foveal centre using spectral domain optical coherence tomography (SD-OCT).
5. BCVA between counting fingers and an Early Treatment of Diabetic Retinopathy Study (ETDRS) letter score of 70, inclusive.

#### Exclusion criteria

##### General

1. Serious allergy to fluorescein or indocyanine green (ICG).
2. Hypersensitivity to alteplase (Actilyse®), gentamicin, arginine, phosphoric acid, polysorbate 80 or aflibercept (Eylea®)
3. Stroke, transient ischaemic attack or myocardial infarction within 6 months.
4. Participation in another interventional study within 12 weeks of enrolment or planned to occur during this study.
5. Women who are breast feeding, pregnant, or planning to become pregnant during the clinical trial. Any sexually active women of childbearing potential must agree continued abstinence from heterosexual intercourse or to use highly effective methods of birth control for the duration up to 12 weeks post IMP administration. Men must also agree to use a condom if their partner is of childbearing potential, even if they have had a successful vasectomy. Females of childbearing potential are females who have experienced menarche and are not surgically sterilised (e.g. hysterectomy or bilateral salpingectomy) or post-menopausal (defined as at least 1 year since last regular menstrual period). Highly effective methods of birth control are those with a failure rate of < 1% per year when employed consistently and correctly, e.g. combined (oestrogen and progestogen containing) hormonal contraception associated with inhibition of ovulation via oral, intravaginal, and transdermal routes; progestogen-only hormonal contraception associated with inhibition of ovulation via oral, injectable, implantable, intrauterine device (IUD), or intrauterine hormone-releasing system (IUS); or vasectomised partner.
6. International Normalised Ratio (INR) greater than 3.5, unless it is anticipated that the INR can be brought below this level prior to vitrectomy, balancing the systemic risks with those of intraocular haemorrhage.
7. Unwilling, unable, or unlikely to return for scheduled follow-up for the duration of the trial.
8. Any other condition which, in the opinion of the investigator, would prevent the participant from granting informed consent or complying with the protocol, such as dementia, mental illness, or serious systemic medical disease.

##### Study eye

9. SMH that is known or estimated to have been present for longer than 15 days, as evidenced by history, pre-trial clinical documentation or fundus appearance.
10. SMH due to eye disease other than exudative AMD.
11. Current active proliferative diabetic retinopathy.
12. Current intraocular inflammation.
13. Current ocular or periocular infection other than blepharitis.
14. Current or known former high myopia (> 6 dioptres).
15. Aphakia.
16. Other current or pre-existing ocular conditions that, in the opinion of the Investigator, will preclude any improvement in BCVA following resolution of SMH, such as severe central macular atrophy or fibrosis, dense amblyopia, macular hole involving the fovea, or very poor BCVA prior to presentation with SMH (counting fingers or worse).
17. Inadequate pupillary dilation or significant media opacities, which will prevent adequate clinical evaluation of the posterior segment or fundus imaging.
18. Intraocular surgery within 12 weeks of enrolment except for uncomplicated cataract surgery, which is permitted within 8 weeks of enrolment.

### Who will take informed consent? {26a}

Sites’ Principle Investigator (PI) or delegated Sub-Investigators (SI) will be responsible for obtaining informed consent from trial participants. Potential participants will mostly be identified from referrals to trial vitreoretinal centres, or otherwise from emergent disease within the PI’s own clinic population. Patients will be invited by the PI or SI to join the study, having had the risks and benefits of participation explained, and having signed a trial-specific informed consent form (ICF). A trial ICF and patient information sheet (PIS) have been included as supplementary materials (Appendix 7 and 8).

### Additional consent provisions for collection and use of participant data and biological specimens {26b}

Participants consent to the collection of routine clinical data from their eye clinic after the study ends, but can withdraw this consent at any time, and remain in TIGER.

Participants are also asked to consent to anonymised data sharing, should other research wish to answer different clinical questions after the study concludes.

## Interventions

### Explanation for the choice of comparators {6b}

EURETINA determined the trial should compare surgery plus anti-VEGF therapy versus anti-VEGF monotherapy.

This comparison appears justified. In our systematic review and synthesis of the literature, we used mostly patient-level data or analyses weighted for study size [7]. We found the best final BCVA outcome was associated with treatment by intravitreal gas, TPA and anti-VEGF therapy (final mean Snellen equivalent 20/66; *n* = 58). The next best was anti-VEGF monotherapy (final BCVA 20/126; *n* = 109). The greatest BCVA gain, versus baseline, however, occurred with vitrectomy, subretinal TPA, gas and anti-VEGF therapy (improving from 20/1002 to 20/171; *n* = 59). There were important differences in the baseline characteristics; most notably, the surgical studies had worse presenting BCVA.

Based on our synthesis of the literature, vitrectomy, subretinal TPA, gas and anti-VEGF therapy not only provides the greatest gain in mean BCVA, but it can accommodate the greatest range of presenting BCVA and SMH size. This therefore constitutes the intervention arm for TIGER.

From a methodological perspective, a novel surgery would ideally be compared to standard of care, but practice surveys indicate a wide range of management approaches. The SOSU study reported that 21% of patients with SMH were managed by observation, 21% by anti-VEGF therapy alone, and 58% by combined vitrectomy, tissue plasminogen activator (TPA), anti-VEGF therapy, and intravitreal gas [2]. It could be argued the standard of care is observation, as patients with SMH were usually excluded from the anti-VEGF registration trials, and there is no licensed treatment for SMH per se. However, anti-VEGF monotherapy is licensed for exudative AMD and so withholding it might be considered unethical, more so as our synthesis of the literature suggests anti-VEGF monotherapy produces outcomes that are far better than observation.

Therefore, using intravitreal anti-VEGF monotherapy as the active-control group appears appropriate, considering the methodological desire to compare to a licensed and established standard of care for wet AMD, and an ethical imperative to avoid a control group with an anticipated poor outcome (natural history).

We selected 2.0 mg aflibercept (Eylea®, Bayer, Leverkusen, Germany) as it is the most commonly used licensed anti-VEGF therapy, with favourable safety and efficacy as a treatment of exudative AMD [9]. Bevacizumab (Avastin) is also available and widely used, but it is not licensed for exudative AMD, and from a regulatory and methodological perspective it is not ideal to compare one IMP with another (off-label TPA vs off-label bevacizumab).

Aflibercept is dosed monthly for 3 cycles then 2-monthly, in accordance with current marketing authorisation. Less intensive dosing regimens are possible, but we aim to maximise the chance of vision gain in both arms. Both arms are dosed identically, except that the first dose in the surgical arm is given at the end of surgery, as this is more comfortable for the participant, and it avoids removal of any intravitreal aflibercept that would occur if aflibercept was given preoperatively.

Ex vivo studies suggest TPA may potentially deactivate aflibercept [10]. However, in TIGER, the TPA is given directly into the SMH, where it works almost immediately, whereas the aflibercept is given later in surgery and in a different compartment (the vitreous cavity versus subretinal space). TPA has an extremely short half-life (4–5 min in plasma) [11] such that when aflibercept diffuses into the subretinal space the TPA is likely to be deactivated, and its purpose already served. Even if TPA did alter the efficacy of aflibercept, this would only affect the first of eight doses.

In the absence of RCT evidence comparing surgery plus anti-VEGF therapy with anti-VEGF monotherapy, we are in equipoise. Case series suggest surgery produces the greatest vision gain, but vision also improves following anti-VEGF monotherapy, and without the risks, inconvenience, discomfort, recovery, post-operative posturing and expense of surgery.^7^

### Intervention description {11a}

As blood is rapidly toxic to photoreceptors excessive delay may mean surgery is less effective, and the surgical risks may start to outweigh the potential benefits. Therefore, it is very important that both screening and surgery are expedited. Ideally, screening is completed in 1 day and surgery scheduled within 3 days of confirmed eligibility.

In cases where the SMH onset is known, it should not have been present for more than 15 days at the point in time when eligibility is confirmed. The maximum time between known SMH onset and surgery is 18 days, for example screening on days 13 and 14 after SMH onset, and surgery 4 days after that. If the onset of SMH is not known, then total time between the start of screening and surgery should be no more than 7 days (if the clinical features suggest SMH has been present > 15 days patients are ineligible, even if this cannot be confirmed by history or pre-trial documentation).

These allowances should not be used to delay surgery, which remains urgent in all cases.

#### Investigational medical product: tissue plasminogen activator

TPA is a 70-kDa glycoprotein enzyme that activates plasminogen to plasmin, which in turn breaks down fibrin clots. Alteplase (Actilyse®, Boehringer-Ingelheim) is a commercially produced TPA manufactured using a recombinant DNA technique and a Chinese hamster ovary cell line. Alteplase is licensed for the treatment of myocardial infarction, acute ischaemic stroke and pulmonary embolism (https://www.medicines.org.uk/emc/product/898/smpc). Alteplase is not licensed for the treatment of submacular clots. TIGER will use vials containing 10 mg of alteplase in powdered form, packaged with a diluent (10 ml of water for injection).

TIGER will select clinical sites that already have access to alteplase for use within its marketing authorisation. Stock alteplase will be relabelled by the site’s Trials Pharmacy according to Annex 13 of Good Manufacturing Practice. Sites will be provided with an Annex 13-compliant template label, which can be adapted in accordance with local and national requirements. Accordingly, alteplase will not need to be shipped to sites.

#### TPA dose

The *maximum dose* of alteplase TPA (Actilyse®) to be used in TIGER is 25 μg, delivered by subretinal injection.

The *concentration* of alteplase TPA (Actilyse®) to be used in TIGER is 100 μg in 1 ml. Using sterile technique, this can be pre-prepared by injecting the 10 ml of the *water for injection* diluent that comes with alteplase, into the 10 mg alteplase vial, then drawing up 1 ml of this solution and making up to 10 ml with *0.9% sodium chloride* for injection. This gives the desired concentration of 100 μg in 1 ml.

Larger dose vials are available, e.g. Actilyse® 50 mg, and can be used if necessary, but a 10-mg vial has more drug than is required for subretinal injection and so larger vials are wasteful and best avoided.

Surgeons can inject up to 0.25 ml of the 100 μg in 1 ml solution (up to a maximum dose of 25 μg) under the retina as a single dose during surgery. The volume required depends on the amount needed to cover the SMH. Typically, most haemorrhages will require no more than 0.1 ml (10 μg). The total volume injected will be recorded in the trial case report forms (CRFs) and electronic case report form (eCRF). TPA should not be injected under the RPE.

If there is a recurrent SMH, then the treatment can be repeated for those in the surgical arm, provided the participant still meets the eligibility criteria. Repeated treatments will be recorded in the TIGER CRFs and eCRF.

#### Required surgeon experience, surgical technique, TPA injection, posturing and post-operative eye drops

Vitrectomy, subretinal TPA injection and gas should be undertaken by a Consultant Vitreoretinal Surgeon who has performed the procedure before. Surgery can also be undertaken by a Senior Vitreoretinal Fellow provided he or she has performed at least 300 pars plana vitrectomies and has done the procedure before. If a Senior Vitreoretinal Fellow or Consultant Vitreoretinal Surgeon has not done the procedure before, they may treat a TIGER participant provided their first case is directly supervised by a Consultant Vitreoretinal Surgeon who has done the procedure before.

Surgery involves the following steps:

- Anaesthesia may be local, with or without sedation, or general anaesthesia, based on participant’s preference and local practice.
- A full 3-port pars plana vitrectomy should be undertaken using 20-, 25- or 27-gauge ports.
- The hyaloid face should be separated from the retina, if it is not already.
- Indented vitrectomy (vitreous base shaving) may be undertaken at the surgeon’s discretion.
- Phacoemulsification and intraocular lens implant may be performed based on the surgeon’s and participant’s preference, in accordance with local practice.
- Peeling of epiretinal membrane (ERM) ± internal limiting membrane (ILM) is allowed if epiretinal membrane and macular pucker are present but should not be done prophylactically. This manoeuvre will be recorded in the CRFs and eCRF.
- Up to 0.25 ml of the 100 μg in 1 ml solution of TPA will be injected sub-retinally (maximum dose 25 μg) as required, depending on the size of the SMH. Typically, most haemorrhages will require no more than 0.1 ml. The total volume injected will be recorded in the TIGER CRFs and eCRF.
- Surgeons should use a subretinal injection cannula of 38-gauge or less. Several companies make these including MedOne, DORC and Synergetics, with marketing authorisation to use as required in this study. The subretinal injection cannula will be connected to a fine bore flexible plastic connecting tube which will be then attached to a 1-ml syringe containing the TPA. The extension cannula and extension tube will be primed with the TPA solution prior to injecting into the subretinal space.
- The TPA can be manually injected by the surgical assistant or using an automated pneumatic injection system [e.g. MedOne MicroDose Injection Kit (https://www.medone.com/microdose-injection-kit)].
- A localised area of retinal detachment will be created with the TPA solution surrounding and enveloping the entire clot. The injection point(s) will be done away from the foveal centre, but in an area of subretinal haemorrhage. The further from the fovea the lower the risk of macular hole formation. Areas of retinal pigment epithelial detachment will be avoided to reduce the risk of sub-RPE injection and RPE tear. Multiple injection points are allowed if clinically indicated.
- The peripheral retina will be then checked carefully for retinal tears and, if present, these will be treated with laser or cryotherapy as clinically indicated.
- Then a full fluid/air exchange will be undertaken and 0.05 ml of aflibercept (Eylea, Bayer) will be injected.
- The above will be followed by an air/gas exchange of 20% sulfahexafluoride (SF_6_) using at least 30 ml of gas. Surgeons can use their local supplier of SF_6_. Since SF_6_ is a commonly used surgical device licensed for intravitreal gas tamponade, it does not require trial labelling.
- Participants will be instructed on how to posture after surgery (see next section).

Post-operative eye drops will be prescribed as detailed below.

The following advice will be given to participants with regard to post-operative head positioning:

- Participants undergoing surgery will be asked to remain on their back for 15 min after the TPA injection, to allow clot liquefaction. This includes any time lying supine during surgery (after the TPA injection).
- After that, participants will be asked to sit up and lean face forward 45 degrees during the day, 50 min out of every hour, for 5 days. During any break from posturing, the participant’s head should be upright. They should be mobile during some of their break time, to avoid deep vein thrombosis and to minimise stiffness.
- Participants will be advised to sleep on their side with the operated eye dependent (e.g. if the right eye underwent surgery, they will be advised to sleep right cheek to pillow). Night-time posture continues for 10 days after surgery. Having the head raised at night by two to three pillows (whilst also maintaining the correct cheek to pillow) may further aid downward displacement and so participants will be advised also to do this.
- It will be recommended to participants to avoid lying on their backs or leaning backwards for 10 days after surgery, to prevent inadvertent subfoveal displacement of the dissolved submacular clot.
- TIGER aims to be a pragmatic trial so inability to completely adhere to the above instructions will not be considered an exclusion criterion and poor compliance will not be considered a protocol deviation. However, participants will be firmly encouraged to posture as best they can. Participants will be asked about their compliance with posturing at each visit for 10 days after surgery, and failure to comply with instructions will be noted in the trial CRFs and eCRFs.

#### Post-operative eye drops

Broad-spectrum antibiotic eye drops will be prescribed for at least 1 week after surgery, and topical steroid eye drops for at least 4 weeks post-operatively. Mydriatics are allowed at the surgeon’s discretion, for approximately 1–2 weeks. The choice of steroid, antibiotic and mydriatic is at the surgeon’s preference, considering also any local policy and the particulars of each participant.

#### Aflibercept

Aflibercept (Eylea®, Bayer) will be used for the treatment of the underlying exudative AMD, reflecting standard of care. Aflibercept use within TIGER is considered to be a non-IMP as it is a background treatment for wet AMD, whereas the object of the trial is to assess surgery (with TPA) against no surgery for SMH. The aflibercept is used fully within its marketing authorisation and given to both arms of the study. Patients with wet AMD would typically be receiving an anti-VEGF therapy such as aflibercept irrespective of their enrolment in TIGER. Both arms of the study will be dosed with a 0.05 ml intravitreal injection of 2 mg aflibercept monthly for three doses, then 2-monthly out to month 12.

Bayer will provide aflibercept (Eylea®) vials free of charge or sites may elect to use their own supplies. Storage, contraindications, precautions, undesirable effects, pharmacological properties and instructions for use, etc., are detailed in the Summary of Product Characteristics (SmPC) (https://www.medicines.org.uk/emc/product/2879/smpc). For participants in the surgical arm, the first injection of aflibercept occurs towards the end of surgery, after fluid-air exchange. Sites may elect to label the aflibercept for trial use, or use standard hospital prescriptions and dispensing, in accordance with local preference/policy and the terms and conditions of Bayer’s provision of free aflibercept to each site (if the site has elected to use Bayer’s free supply).

The manufacturer provides a guide to Eylea® for patients with wet AMD (https://www.medicines.org.uk/emc/rmm/617/Document). Sites are encouraged to provide this or their own information on Eylea® to participants, but it should be explained that the co-existence of SMH will adversely affect prognosis.

Patients currently receiving another anti-VEGF drug will need to swap to aflibercept for the duration of the study, and this should be discussed with them. Likewise, arrangements for continuation of aflibercept at the end of the trial, or change to another anti-VEGF agent, should be discussed with the participant, so that they are aware of the locally available anti-VEGF agents.

### Criteria for discontinuing or modifying allocated interventions {11b}

#### Positioning

Occasionally, the post-operative participant position might need to be altered due to the location of the submacular blood following TPA. For example, if the blood is predominantly nasal to the fovea the participant might be advised to sleep on the opposite cheek. Any atypical positioning should be documented.

#### TPA

Since TPA is given as a single dose during surgery, there are no treatment stopping rules. Repeat surgery and TPA are allowed for recurrent SMH, but only in the surgical arm and only if the eligibility criteria are still met.

#### Aflibercept

Aflibercept (Eylea®) treatment should be discontinued as described in the current SmPC (https://www.medicines.org.uk/emc/product/2879/smpc). Presently, the SmPC states: ‘If visual and anatomic outcomes indicate that the patient is not benefiting from continued treatment, Eylea should be discontinued’. It is important to note that SMH may make it difficult to determine if the participant is responding to aflibercept. In this setting, Investigators should give participants the ‘benefit of the doubt’ and continue treatment unless they are absolutely certain that treatment is not helping. The reason is that apparently unresponsive vision may yet improve when the SMH resolves, and meanwhile it is important to control the underlying disease to maximise the final visual recovery. Aflibercept may also promote SMH clearance and reduce the risk of additional haemorrhage. Hence, investigators are strongly advised that all participants receive all scheduled doses of aflibercept, namely monthly for 3 doses then 2-monthy until month 12 inclusive.

### Strategies to improve adherence to interventions {11c}

Investigators are required to explain the post-operative positioning regimen to participants and provide them with a standardised trial document detailing how to posture after surgery. In general, compliance with anti-VEGF therapy is satisfactory within clinical trials, but sites will be encouraged to explain the importance of maintaining aflibercept therapy in both arms.

### Relevant concomitant care permitted or prohibited during the trial {11d}

Treatment for AMD, IPCV, RAP and cataract should be limited to the following, but treatment of fellow eye or systemic diseases should be as clinically indicated:

#### Photodynamic therapy for idiopathic polypoidal choroidal vasculopathy

Polyps can be treated with photodynamic therapy (PDT) if that is the site’s usual practice. Aflibercept therapy should continue. The reading centre’s determination of whether or not polyps are present is not to be communicated to sites. This is so that a given site’s ability to detect and treat polyps with PDT will reflect real-world decisions making the results of TIGER more generalisable. Therefore, the site should make their own determination as to whether or not polyps are present, and if polyps are found, they should likewise make their own decision whether or not to offer their participant PDT.

#### Reoperation for recurrent submacular haemorrhage

The SOSU study estimated that 20% of cases have a rebleed within 6 months at a mean time of 96 days [2]. For those who do, repeat surgery (vitrectomy, subretinal TPA and SF_6_) is permitted provided the TIGER eligibility criteria are still met, and they were originally randomised to the surgical arm. More than one repeat surgery is permitted if eligible SMH recurs more than once. Participants originally allocated to the control arm should continue on aflibercept monotherapy.

#### Cataract surgery (including phakovitrectomy)

At the first TIGER study, group meeting some surgeons advocated phakovitrectomy to avoid the cost and inconvenience of two operations; others preferred sequential cataract surgery as biometry is more reliable after the SMH resolves. For TIGER, either approach is permitted, to reflect local service provision. Regular lens grading will be undertaken during follow-up visits and recorded in the trial CRFs and eCRF, for both arms of the study. Investigators are encouraged to promptly treat emerging cataract in either arm, at least 8 weeks prior to the month 12 visit.

It is recognised that removal of pre-existing subclinical lens opacity, and conversely failure to remove developing post-vitrectomy cataract, may both alter BCVA. However, it is not desirable to exclude phakic patients or cataract surgery as that would reduce recruitment and trial generalisability. It is understood that any vision gain in the surgical group may come partly from cataract surgery, but the trial considers the surgical pathway under investigation to include cataract surgery as needed. A subgroup analysis in pseudophakes will be undertaken and aims to help understand the mechanism of any vision gain.

### Provisions for post-trial care {30}

After exiting, the trial participants will revert to standard care. The Sponsor has insurance to cover negligent harm to trial participants outside of the UK; negligent harm within the UK is covered by the National Health Service Clinical Negligence Scheme for Trusts.

### Outcomes {12}

#### Primary efficacy outcome

The primary efficacy outcome is the proportion of participants with a BCVA gain ≥ 10 ETDRS letters from baseline in the study eye at the 12-month visit. This efficacy outcome enables future patients, post-trial, to weigh the likelihood of a meaningful vision gain versus the downsides of surgery, as advocated by patients at an advisory focus group meeting organised to seek patient input on trial design. The European Medicines Agency reports that 10 letters is the minimum clinically relevant amount perceived by patients, [12] and is associated with a clinically significant gain in the National Eye Institute (NEI) Visual Function Questionnaire (VFQ-25) composite score in patients with macular disease [13]. It is greater than the 6.5-letter ETDRS test-retest variability in eyes with AMD [14].

A 12-month endpoint allows time for SMH to resolve, and emerging cataract to be treated.

#### Secondary efficacy outcomes

The following secondary efficacy outcomes will be reported, with respect to the study eye:

- Proportion of participants with BCVA gain ≥ 10 ETDRS letters from baseline (at 6 months).
- Mean BCVA measured by ETDRS letters (at 6 and 12 months).
- Maximum reading speed (words per minute) assessed by Radner reading acuity (at 6 and 12 months).
- Area of central visual scotoma size using Humphrey Field Analyser 10-2 or equivalent (at 6 and 12 months).
- National Eye Institute Visual Function Questionnaire-25 (NEI VFQ-25) composite score (at 6 and 12 months).
- EQ-5D-5L questionnaire with vision bolt-on proportion of categorical dimension responses and mean visual analogue scale (at 6 and 12 months).
- Presence or absence of subfoveal fibrosis and/or atrophy and area of fovea-involving fibrosis/atrophy assessed using multimodal imaging by an independent reading centre, combining spectral domain optical coherence tomography (SD-OCT), fundus autofluorescence (FAF) and stereo fundus photographs (at 12 months).

Unless otherwise stated above, continuous outcome measures (per treatment arm) will be reported at the specified time points as mean and standard deviation, or median and interquartile range where there is extreme skewness. Categorical outcome measures (per treatment arm) will be summarised at the specified time points as count and percentage. Safety outcomes are detailed below, in the section ‘Adverse event reporting and harms {22}’

### Participant timeline {13}

**Fig. 1:**
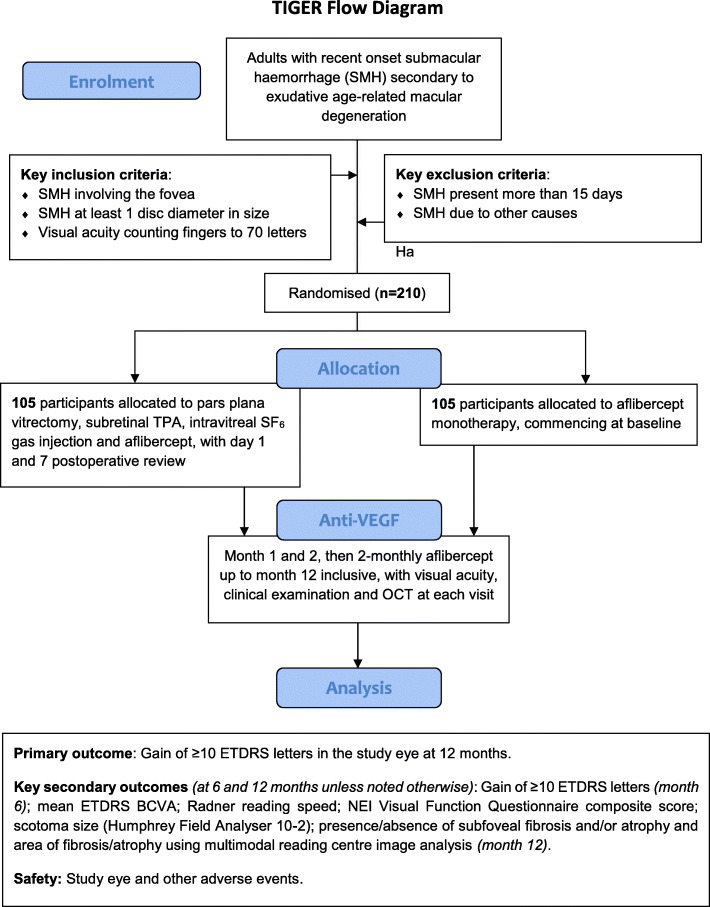
Trial Participant Flow Diagram

Following successful screening, participants are randomised to surgery or standard care. Those undergoing surgery are seen at day 1 and week 1 post-operatively. All participants are then seen monthly for 2 months, then 2-monthly until month 12 inclusive. The schedule of procedures is shown in Table 1. A participant flow diagram is shown in Fig. 1.

**Table 1:**
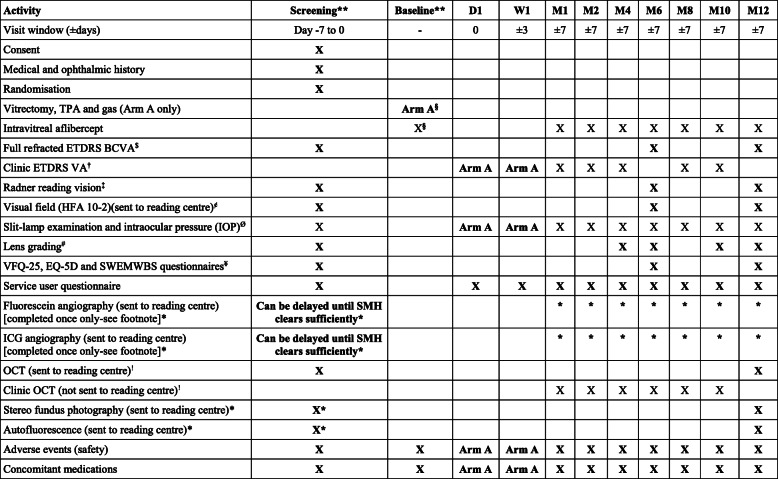
Schedule of Activities

**As blood is rapidly toxic to photoreceptors excessive delay may mean surgery is less effective, and the surgical risks may start to outweigh the potential benefits.

Therefore, it is very important that both screening and surgery are expedited. Ideally, screening is completed in 1 day and surgery scheduled within 3 days of confirmed eligibility. Screening and surgery can occur on the same day, to avoid delay. In cases where the SMH onset is known, it should not have been present for more than 15 days at the point eligibility is confirmed. The maximum time between known SMH onset and surgery is 18 days, for example screening on days 13 and 14 after SMH onset, and surgery 4 days after that. If SMH onset is unknown, then the total time between the start of screening and surgery should be no more than 7 days (if the clinical features suggest SMH has been present >15 days patients are ineligible, even if this cannot be confirmed by history or pre-trial documentation). These allowances should not be used to delay surgery, which remains urgent in all cases.

$ Full refracted ETDRS VA should be undertaken in both eyes separately at baseline and month 12 and study eye only at month 6. Details in Appendix 1.

† Clinic ETDRS VA should be undertaken in the study eye using an ETDRS chart and distance spectacle correction if worn, with and without pinhole, but otherwise according to the site’s usual technique.

‡ Radner reading vision should be measured in the study eye only at screening and month 6, but both eyes at month 12. Details in Appendix 1.

¢ Visual field tests should be completed in study eye only, scanned, and sent to the Reading Centre, as detailed in Appendix 2.

Ø Slit lamp examination and IOP should be undertaken in the both eyes at screening, month 6 and month 12, and study eye only at other visits.

# Age-related Eye Disease Study (AREDS) lens grading is in both eyes at screening and month 12; study eye only at months 4, 6 and 10.

¥ The VFQ-25 questionnaire should also include the ‘optional’ questions listed at the end in the appendix and the EuroQol questionnaire includes the 5-item vision bolton (EQ-5D-5L). SWEMWBS=Short Warwick-Edinburgh Mental Well-being Scale.

§ The initial intravitreal aflibercept injection can be administered on the day of screening, after eligibility is confirmed, in those randomised to the non-surgical group. In those randomised to surgery (Arm A), aflibercept is injected towards the end of surgery, straight after fluid-air exchange.

! At screening and month 12 ‘per protocol’ OCT images should be obtained in both eyes using certified staff and equipment, as per the Reading Centre’s instructions.

These images are sent to the Reading Centre. At other visits a ‘Clinic OCT’ should be acquired in the study eye only, using standard staff and methodology. ‘Clinic OCT’ images are not sent to the reading centre. The attending clinical investigator should review all OCTs to monitor progress, watch for any emergent adverse events, and measure subretinal haemorrhage height. The same OCT machine should be used on a given participant throughout the study.

*Fluorescein and ICG angiography should be acquired once only. Either or both should be delayed if needed, to allow the SMH to clear sufficiently to enable visualisation of choroidal neovascularisation and/or polyps. The screening stereo fundus photography and fundus autofluorescence (FAF) should be repeated with delayed angiography, to help interpretation by the Reading Centre. Clinical investigators should review imaging to detect any emergent adverse events

### Sample size {14}

To calculate our sample size, we undertook a bracketing exercise with our patient focus group as detailed below in the Public and Patient Involvement section. We asked them what improvement in ‘treatment success’, defined as meaningful gain in vision, they would require to undergo eye surgery, with the attendant downsides such as discomfort, head positioning, complications, recovery and possible cataract surgery, and assuming a 1 in 4 chance of success with anti-VEGF injections alone [7]. We defined treatment success as a 10 letter gain in the ETDRS letter score (for justification see the section ‘Primary efficacy outcome’).

The findings ranged from one patient who would not want surgery ‘at my age’, regardless of outcome, to another who would have surgery even if it improved his success from only 25 to 26%. The most common response was that patients wanted at least a 50% chance of success to consider vitrectomy.

What also emerged in this elderly patient focus group was a consensus that they wanted the doctor to make the best decision on their behalf. Paradoxically then, our patient focus group exercise led us to ask 10 ophthalmologists, from junior to senior, across a range of subspecialities, what they would do, ‘if it was their eye’. We asked them to assume they were older adults with ≈25% success with anti-VEGF therapy. The median ‘success rate’ needed to consider surgery differed if it was their potentially better or worse seeing eye (47.5% vs 55% respectively), but overall the average, median, and mode were 49%, 50% and 50% respectively. Thus, we used 50% as the minimum success rate needed to justify surgery.

Our synthesis of the literature [7] found that 27% of patients receiving anti-VEGF monotherapy for AMD-related SMH gained 2 Snellen lines (≈10 letters).

A two-group *χ*^2^ test with a 5% two-sided significance level has 90.62% power to detect a difference between a Group 1 proportion, π1, of 0.27 and a Group 2 proportion, π2 of 0.5 (odds ratio 2.704) when the sample size in each group is 94 (NQuery Advanced software v 8.2.1). With ≈12% attrition, the sample size inflates to 210 participants.

### Recruitment {15}

**Fig. 2:**
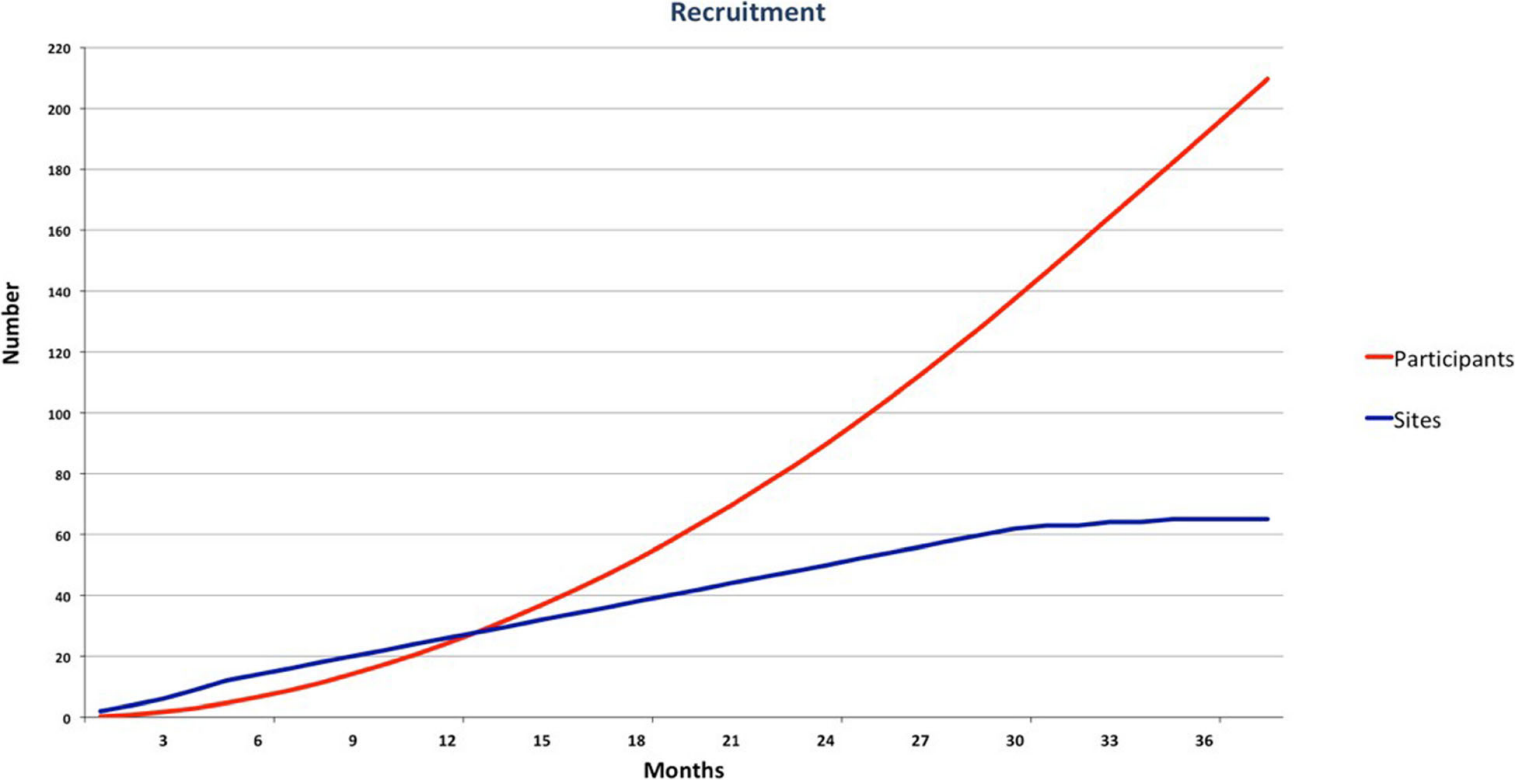
Recruitment Projection Diagram

The estimated annual incidence of large SMH meets the criteria of a rare disease [4] and recruitment estimates need to be realistic. At an initial TIGER study, group meeting sites’ recruitment estimate averaged 0.83 participants per month. However, sites may fail to deliver as predicted. Our TAPAS pilot (ClinicalTrials.gov Identifier NCT01835067) averaged 0.28 participants/site/month, but with 65 sites, TIGER cannot choose sites as selectively, so we estimate half this, at 0.14 participants/site/month. This matches the recruitment numbers of the STAR study (ClinicalTrials.gov Identifier: NCT02557451; personal communication, Professor Catherine Creuzot, Chef du Service d’Ophtalmologie, CHU, Dijon). Accordingly, recruitment of 210 participants is expected to take approximately 38 months, as shown in Fig. 2.

Recruitment tools include:

- Shared academic outputs
- Completed study group meeting to design a workable protocol
- Pragmatic eligibility criteria
- Working relationship with multiple sites from prior vitreoretinal RCTs [15–18]
- Monthly recruitment letter with league table
- Posters for patients, doctors and imaging technicians
- Slide deck for Principal Investigators (PIs)
- Regular coordinator and investigator meetings, ‘operations’ calls and CI-PI contact
- Co-PI network of junior clinicians
- Performance monitoring using eight established core metrics [19]
- Recruitment tools checklist verified alongside monitoring visits
- Publicity via Euretina, BEAVRS, EVRS, eye charities, Twitter, YouTube, Google+ and study website
- Investigators’ WhatsApp group
- Collaboration with recruitment expert (Professor Treweek; https://www.trialforge.org) [20]

## Assignment of interventions: allocation

### Sequence generation {16a}

Randomisation is at the participant level via Elsevier MACRO electronic online data capture system (http://www.ctu.co.uk/). Randomisation is 1:1 (surgery:control) using the method of minimisation, stratified by the following factors:

1. Study site
2. Lens status: phakic or pseudophakic
3. SMH size: fully within the retinal vascular arcades, or not (i.e. extending beyond the vascular arcades)
4. BCVA: ≥ 35 letters or not, equivalent to 6/60 and near to the median presenting BCVA in the TAPAS study of SMH secondary to exudative AMD (ClinicalTrials.gov identifier: NCT01835067)

To reduce predictability of the random sequence, 10% random component was implemented in the minimisation procedure.

### Concealment mechanism {16b}

After registration on the system, randomisation and allocation will occur centrally using MACRO’s online randomisation servers, and an email with the allocated study treatment will be sent to the individual site investigator.

### Implementation {16c}

The trial ophthalmologist (the PI or delegated SI) will consent and enrol participants onto TIGER, and randomisation and allocation will occur centrally immediately following enrolment using MACRO’s online servers. Blood is rapidly toxic so that those randomised to surgery should be scheduled for vitrectomy, TPA, gas and aflibercept as a matter of urgency. Timelines are described in the footnote to Table 1.

## Assignment of interventions: blinding (masking)

### Who will be blinded (masked) {17a}

It is not possible to mask participants to their allocation because there is no sham for vitrectomy, and intravitreal gas is easily visible to participants. However, the primary efficacy outcome (proportion of participants with BCVA gain ≥ 10 ETDRS letters from baseline at 12 months) will be assessed by masked observers using an established protocol, encouraging participants to ‘try their hardest’, to minimise any differences in decision criterion and measurement bias. Secondary BCVA assessments will also be undertaken by masked observers. Scotoma size will be measured by a device that uses an automated algorithm.

Image analysis (providing the structural secondary efficacy outcomes) will be conducted by certified, masked graders at an independent reading centre (The Network of Ophthalmic Reading Centres UK, NetwORC UK).

Patient-reported outcomes (NEI VFQ, EQ-5D-5L and SWEMWBS) will potentially be the outcomes at greatest risk of bias due to the participant’s knowledge of treatment allocation, but this will be minimised by trial information promoting equipoise between the two interventions.

The aflibercept dosing regimen is mandated, and so does not rely on physician or patient decisions. A junior statistician will, if requested, provide unmasked data to the Data Monitoring and Ethics Committee. The lead trial statistician will be masked with respect to his analysis of the primary and secondary efficacy outcomes.

### Procedure for unblinding if needed {17b}

Not applicable.

## Data collection and management

### Plans for assessment and collection of outcomes {18a}

ETDRS is generally the most accepted BCVA test and is used for most AMD registration studies. It will be determined after full refraction by masked, certified examiners using a certified room and certified equipment, at baseline (both eyes), month 6 (study eye) and month 12 (both eyes), commencing at 4 m. The protocol for ETDRS testing is well established [21]. Details of the testing routine are provided in Appendix 1.

Because SMH may lead to a central scotoma, TIGER measures central visual field using the automated Zeiss Humphrey Field Analyser (HFA3, Carl Zeiss Meditec, Dublin, USA). The Humphrey field analyser is probably the most established visual field testing device. Details of the testing routine are provided in Appendix 2.

A central scotoma may affect a patient’s ability to read, so TIGER measures reading vision using the Radner Reading Charts. The Radner Reading Charts were developed around the concept of ‘sentence optotypes’ for the examination of reading acuity and speed [22]. Print sizes are geometrically (logarithmically) scaled. Reading acuity is given in logRAD (logReading-Acuity-Determination) to permit statistical analysis, and the results obtained can be compared to other logarithmically scaled vision systems (e.g. logMAR) [23]. Reading speed is analysed in words per minute (wpm). To guarantee accurate, reproducible and standardised measurements of reading speed and reading acuity, ‘sentence optotypes’ have been created to minimise the variations between the test items. Through interdisciplinary cooperation, a series of test sentences were developed that are highly comparable in terms of the number of words (14 words), as well as the word length, number of syllables, position of words, lexical difficulty and syntactical complexity. The most similar sentences were statistically selected for the Radner Reading Charts. The Radner Reading Charts have then been statistically evaluated in terms of test-retest reliability, inter-chart reliability and a variance component analysis [22]. Testing will be undertaken by trained, certified, masked assessors following full refraction with near correction, in the study eye at baseline and month 6, and in both eyes at month 12.

The Radner maximum reading speed is an important secondary efficacy outcome, but two additional measurements will be acquired alongside this:

- Reading acuity (unit, logRAD, in 0.1 log steps): Equivalent to the logarithm of the minimum angle of resolution (logMAR) distance acuity. This corresponds to the smallest sentence optotype read accurately in less than 30 s.
- Maximum reading speed (words per minute): fastest, accurate reading speed achievable at any sentence optotype.
- Mean reading speed (words per minute): average of the fastest 3 sentences optotypes read accurately, with optotype size from 0.9 logRAD to 0.3 logRAD. Omit if fewer than 2 sentence optotypes were read accurately.

The NEI VFQ-25 visual function questionnaire is a well-established, validated, patient-reported outcome measure (PROM) that has been used extensively in AMD studies [24].

To enable determination of quality-adjusted life years (QALYs), participants will complete the EQ-5D-5L with vision bolt-on questions. The EQ-5D-5L is a validated generic, health-related, preference-based measure comprising five domains: mobility; self-care; usual activities; pain and discomfort; anxiety and depression. Each domain has five levels. The questions are complemented by a visual analogue scale, with 0 representing the worst imaginable health and 100 representing the best imaginable health. We chose this version with 5 levels anticipating that it may be more sensitive than the 3 L version in this population, potentially avoiding ceiling effects.

It is possible that loss of vision from SMH has a particular impact on mental health. We therefore include the 7-item Warwick-Edinburgh Mental Wellbeing scale short form (SWEMWBS) as an exploratory outcome measure. The SWEMWBS questionnaire is widely used and well validated (https://warwick.ac.uk/fac/sci/med/research/platform/wemwbs/. PROMs are collected at baseline, month 6 and month 12.

Paper case report forms (CRFs) for data collection will be made available to sites with the site investigator file (ISF) as well as to download via a password-protected section of the study website (www.tigerstudy.org.uk). The password will only be made available to study investigators and named members of the research teams at individual sites, coordinated centrally.

### Plans to promote participant retention and complete follow-up {18b}

Retention in wet AMD trials is usually good, and given the acute and vision-threatening nature of SMH and the need for regular anti-VEGF therapy, we anticipate satisfactory retention. Nonetheless, the protocol, site initiation visits, investigator meetings and regular newsletters will all aim to impress on sites the importance of retention. Study activity feedback will be periodically collected from recruited participants to identify any arising or recurring issues with the consent and recruitment, screening, surgery or follow-up activities. Participants who choose to discontinue will be asked to complete an early withdrawal visit (which will include the same datapoints as for the study month 12 exit visit) and the reason for withdrawal will be documented.

### Data management {19}

Sites will be provided with paper CRFs, for use alongside clinical visits. There will be a corresponding, online, secure, electronic case report form (eCRF). Sites will transfer data from the trial paper CRFs to the eCRF. The data will reside on an online, secure, trial database; Elsevier MACRO electronic data capture system (http://www.ctu.co.uk/). MACRO is designed specifically for clinical research, provides full audit capacity, and is compliant with relevant UK and European Union data protection legislation, and Good Clinical Practice.

A web-based electronic data capture (EDC) system will be designed, using the InferMed MACRO 4 system. The EDC will be created in collaboration with the trial analysts and the CI and maintained by the King’s Clinical Trials Unit (KCTU) for the duration of the project. It will be hosted on a dedicated server within King’s College London (KCL). In addition to the main trial database, a secondary MACRO database will be created to host the data from the Independent Reading Centre (IRC). The IRC staff will have access to this secondary database to enter their reading data, but only a limited number of unmasked IRC staff will have access to the main database, to avoid unmasking, but to help the IRC track participant withdrawals and image collection.

At the database design stage, validations will be programmed into the system to minimise data entry errors by querying the data entered in real time with sites.

The CI or delegate will request usernames and passwords from the KCTU. Database access will be strictly restricted through user-specific passwords to the authorised research team members. It is a legal requirement that passwords to the EDC are not shared and that only those authorised to access the system are allowed to do so. If new staff members join the study, a user-specific username and password will be requested via the CI or delegate (e.g. Trial Manager) from the KCTU team and a request for access to be revoked will be similarly requested when staff members leave the project. Study site staff experiencing issues with system access or functionality will contact the CI or delegate (e.g. Trial Manager) in the first instance.

At the end of the trial, the site PI will review all data for each participant and provide electronic sign-off to verify that all the data are complete and correct.

Upon request, KCTU will provide a copy of the final exported dataset to the CI in .csv format. A copy of the full raw dataset is to be stored in the TMF.

Data monitoring will be undertaken by King’s Health Partner’s Clinical Trial’s Office (KHP-CTO) according to established Standard Operating Procedures, available at in Appendix 3.

### Confidentiality {27}

All data leaving recruiting sites will be anonymised and stored on a secure, password-protected database (MACRO). On site, data will be stored in secure password-protected computers or paper CRFs stored in locked research facilities with restricted access.

### Plans for collection, laboratory evaluation and storage of biological specimens for genetic or molecular analysis in this trial/future use {33}

Not applicable.

## Statistical methods

### Statistical methods for primary and secondary outcomes {20a}

The primary analysis will be conducted by the lead trial statistician, following the intent-to-treat principle where all randomised participants are analysed in their allocated group, whether or not they receive the treatment they were allocated to following randomisation. Baseline characteristics will be summarised for the two treatment groups. Continuous data will be summarised using means and standard deviations for data that follow a normal distribution or medians and interquartile ranges. Binary data will be reported as frequencies and percentages.

The primary efficacy outcome is whether or not the participant gains at least 10 ETDRS letters in their study eye at the month 12 visit. This will be compared between treatment groups using logistic regression, which will provide an effect estimate (odds ratio) and compare the proportions gaining at least 10 letters after adjustment for randomisation stratifiers. We will also report the difference in proportions of participants with an improvement in BCVA score of 10 letters or more with a two-sided confidence interval.

Secondary continuous efficacy outcomes measured at randomisation and on more than one occasion during follow-up will be analysed using a linear mixed effect model. The value at baseline, treatment group, follow-up time and the stratifying variables will be included as fixed effects. Model assumptions will be assessed, and a logarithmic transformation used if this improves normality of residuals. Secondary dichotomous outcomes will be examined using the same techniques as the primary analysis.

All statistical tests will use a 2-sided *P* value of 0.05 unless otherwise specified. All confidence intervals will be two-sided and 95%. A detailed Statistical Analysis Plan will be finalised prior to or near the start of recruitment, which will be circulated for comment to the Trial Steering Committee (TSC) and Data Monitoring and Ethics Committee (DMEC) for comment.

### Interim analyses {21b}

No formal interim analysis or pre-specified trial stopping rules are planned, but reports concerning participant safety and key outcomes will be reviewed at least yearly by the DMEC. If safety concerns are identified, the DMEC may choose to meet more frequently, and on an ad hoc urgent basis. The DMEC can request unmasked data if required. If necessary, for urgent safety reasons, the Sponsor may stop or pause the trial immediately, without DMEC review.

### Methods for additional analyses (e.g. subgroup analyses) {20b}

A pre-specified subgroup analyses will assess the possible interactions between treatment and each of the following parameters:

- *Lens status (phakic vs pseudophakic)*: Removal of pre-existing cataract may improve vision, whereas post-vitrectomy lens opacity that does not trigger cataract surgery in the 12 months’ follow-up period may reduce vision. This may impact on our analysis of the effect of SMH surgery. It is much less problematic if we aim for a pragmatic trial design, wherein routine cataract management forms part of the ‘real-world’ patient pathway. However, we realise some clinicians and reviewers will want to isolate the effects of vitrectomy from lens events, and for them an analysis of pseudophakic eyes will provide a useful mechanistic insight.
- *SMH size (fully within the retinal vascular arcades, or not)*: Many clinicians believe that large SMHs do better with surgery, as vitrectomy, TPA and gas is expected to provide more complete and rapid removal of blood from the fovea. We have chosen this size as it is a pragmatic and clinically useful size differentiator. Outcome has previously been shown to be closely related to the size of the haemorrhage. A SMH of less than 30 mm^2^ (approximately just up to the arcades) has been shown to be predictive of an outcome of 6/60 or better in cases treated with vitrectomy and subretinal TPA [25]. The determination of SMH size will be made by the clinical investigator at screening, so that results are generalisable to post-trial care, without reliance on a reading centre.
- *Lesion type (choroidal neovascularisation vs idiopathic polypoidal choroidal vasculopathy)*: Some clinicians consider IPCV as part of the AMD spectrum, others consider it to be a distinct entity. Therefore, we cannot assume the effects of surgery will be the same in those with/without IPCV. IPCV is an important cause of SMH and is over-represented in case series of AMD with SMH. It is more common in Black and Asian patients. Retrospective studies report that SMH due to CNV has a worse outcome than SMH due to IPCV [26]. IPCV is often associated with blood below the RPE, and this space is not accessed via a sub-neuroretinal injection. Therefore, it is possible that surgery may be less effective for IPCV than CNV.
- *Duration of SMH (≤ 7 days vs > 7 days)*: It is possible that the benefits of surgery may diminish with a longer duration SMH, if the sustained toxic effects of blood reduce the impact of blood removal, yet the surgical risks remain constant. We chose 7 days as numerous studies have suggested that SMH duration of less than 7 days is associated with an improved visual outcome in surgically treated cases. These studies have typically been retrospective, and the time point chosen arbitrarily based on previous publications and animal studies. Regardless, it is an easy timepoint for clinicians to use and remember if it is found to be important.

These factors will be explored by adding interaction terms to the regression model for the primary outcome.

### Methods in analysis to handle protocol non-adherence and any statistical methods to handle missing data {20c}

Every effort will be made to avoid missing data; however, we acknowledge that some is inevitable. How it is dealt with will depend on what is missing. We will report missingness wherever present. Reasons for missingness may be important and these will be investigated using logistic regression of covariates on an indicator of missingness.

Sensitivity analysis will investigate the validity of the missing data completely at random assumption and will explore imputation for missing data. In relation to the primary outcome variable, we will conduct an available case analysis but will then conduct a worst-case best-case analysis to examine the impact of missing data. The sensitivity analysis will consider participants in the surgery group with missing outcomes having a meaningful change in BCVA and participants in the anti-VEGF monotherapy control group with missing data not having a meaningful change in BCVA, and then the opposite. Our missing data analysis is complete if the results show that they are consistent with the available case analysis. If not, a range of more plausible assumptions will be explored following principles laid out in Carpenter & Kenwood [27].

We will examine patterns of missingness and the reasons which caused the data to be missing. This will be achieved by examining the observed data and reasons for withdrawal in discussion with the clinical investigators. We will use this information to derive a series of missing data models. We will use these models to impute values in order to undertake a sensitivity analysis of the treatment effect estimate. If data are thought to be missing at random (MAR), conditional on additional variables not included in the primary analysis model, then the treatment effect will be estimated conditioning on the identified variable for example: conditioned on BCVA or size of haemorrhage at baseline. The scenario of missing not at random scenarios (MNAR) will be explored using a range of plausible assumptions and viewed graphically using a mean score approach via the rctmiss procedure in stata [http://www.mrc-bsu.cam.ac.uk/software/stata-software]. The impact of missing data will be mitigated against by incorporating information from earlier timepoints using the mixed model approach.

### Plans to give access to the full protocol, participant-level data and statistical code {31c}

Following publication of the main results, and subject to participant consent and Research Ethics Committee (REC) approval in each area, we aim to make anonymised data available for secondary research provided the proposal is compliant with the General Data Protection Regulation (2016/679, or relevant subsequent legislation) and the UK’s Medical Research Council Policy on Data Sharing, including safeguards of scientific quality, ethical approval, a publicly available pre-specified protocol and acknowledgments of the study group and funders. The full protocol is available in Appendix 4. The statistical coding will be made available.

## Oversight and monitoring

### Composition of the coordinating centre and trial steering committee {5d}

A Trial Steering Committee (TSC) includes co-applicants, trial managers, statisticians, Chief Investigator (CI), lay representative (from the Macular Society) and clinicians. There is an independent Chair, an independent statistician and an independent voting majority, and the TSC will meet at or near trial commencement, and then approximately 6–12 monthly. A TSC charter details the membership, roles and responsibilities of the committee and is available in Appendix 5. The coordinating centre (King’s College Hospital) will hold the trial master file (TMF) and liaise with all trial sites to ensure administrative requirements for effective trial conduct are delivered—including running site initiation visits (SIV), addressing site queries and monitoring and coordinating data collection. The coordinating centre is comprised of the CI, trial managers and research associates.

**Fig. 3:**
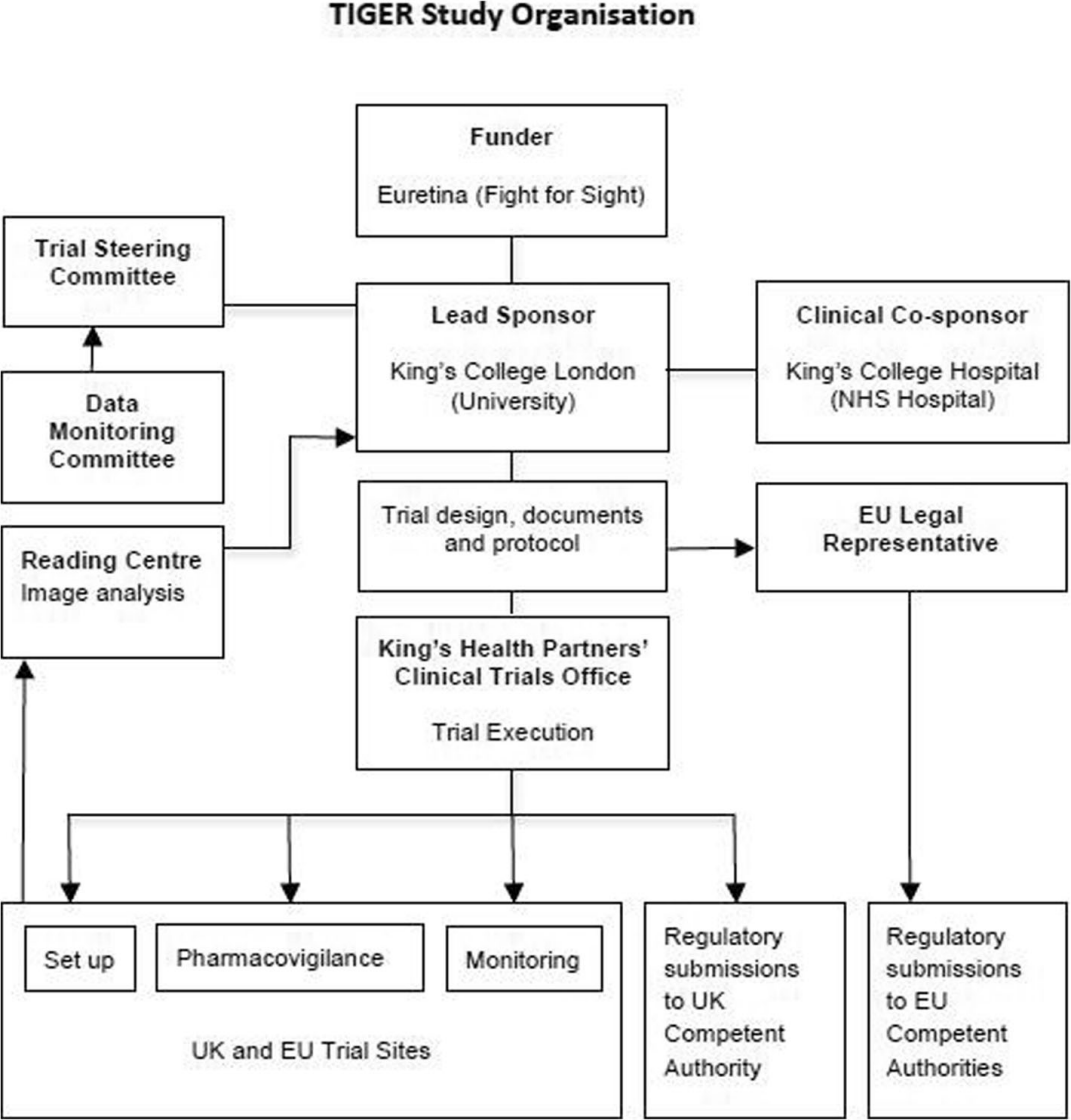
TIGER Study Organisation

An overview of trial organisation is shown in Fig. 3.

### Composition of the data monitoring committee, its role and reporting structure {21a}

A Data Monitoring and Ethics Committee (DMEC) comprises of 2 independent clinicians and an independent statistician chair. The DMEC will regularly review safety data approximately every 6–12 months and make recommendations on whether the trial should continue, stop or be modified based on their findings to the TSC and Sponsor via the DMEC chair. A DMEC charter details the membership, roles and responsibilities of the DMEC and is provided in Appendix 6.

### Adverse event reporting and harms {22}

Safety assessments include regular measurement of BCVA, clinical examination, IOP and OCT imaging. The final visit includes masked multimodal imaging read by an independent reading centre, to look for and quantify any structural damage.

Participants will be reviewed at 1–2-month intervals throughout most of the study, with shorter intervals following surgery, and investigators will ask specifically if participants have experienced any new symptoms or AEs at each visit.

All AEs, SAEs and important medical events (IMEs) will be recorded on the paper CRFs and uploaded onto the eCRF by investigators.

AEs and SAEs will be reported by study group, coded using the Medical Dictionary for Regulatory Activities (MedDRA) Preferred Terms. Study eye AEs and SAEs will be reported separately. The site’s Principal Investigator will determine causation in relation to surgery, gas, TPA, aflibercept and intravitreal injections, classified as unrelated, remote, possible, probable and definite, in discussion with the CI if necessary. Study eye SAEs will be reported by severity (mild, moderate or severe).

Reference safety information is available in section 4.8 of the Actilyse® (Alteplase) Summary of Product Characteristics dated May 2019 as approved [11]. AE, SAE, IME and other definitions and reporting details are specified in the full protocol (Appendix 4).

Certain events do not require reporting as AEs. Loss of vision as a result of disease progression and other events that are primary or secondary outcome measures are not considered to be SAEs and should be reported in the normal way, on CRFs and corresponding eCRF.

Adverse events occurring after successful enrolment should be reported, except for loss of vision, as that will be captured in the efficacy analysis. Events that are commensurate with, and typical of, exudative AMD (CNV, IPCV or RAP) need not be reported, such as macular fluid leakage, exudates, pigment epithelial detachment, macular hypo- or hyperpigmentation, geographic atrophy, small amounts of retinal haemorrhage and RPE rips. However, if these changes are not thought to be explained by the underlying disease, then they should be reported. Breakthrough vitreous haemorrhage is well described with SMH, but since it could also potentially be aggravated by TPA it should be reported as an AE or SAE, as appropriate.

### Frequency and plans for auditing trial conduct {23}

Monitoring of the CTIMPs at King’s Health Partners is delegated to the KHP-CTO, who monitor independently of the trial team, according to established SOPs (Appendix 3).

### Plans for communicating important protocol amendments to relevant parties (e.g. trial participants, ethical committees) {25}

Substantial amendments will be notified to the relevant Competent Authority by the KHP-CTO, or by the Sponsor’s legal representative in the European Union. REC amendments will be submitted by the central trial team, or their country or site delegates as appropriate, depending on local and national guidelines. The TSC will be appraised of all substantial amendments and asked to approve them at the next meeting (or more urgently if required).

### Dissemination plans {31a}

We aim to publish the pre-specified efficacy and safety outcomes paper in a high-quality peer-reviewed publication. The planned journal outputs offer a Creative Commons Attribution (CCBY) license with open access (included in the budget). We will present the results at Euretina and provide a slide deck for Investigators to present results locally. We will issue a press release via our Communications Office. The Macular Society will help publicise results via their newsletter, website and meetings and Euretina plan to publicise the results via their website. We will write to participants outlining the main results and post a lay summary on the planned study website. The patient focus group and Macular Society will review key communications. Participants can choose a large font letter, electronic or audio material.

#### Public and Patient Involvement (PPI)

We convened a focus group meeting of patients with SMH secondary to wet AMD during the design phase of the trial, to incorporate their perspective into the TIGER study proposal and protocol. Three were on our pilot study of intravitreal gas/TPA/anti-VEGF and two had the same treatment outside of TAPAS (ClinicalTrials.gov Identifier: NCT01835067).

All patients agreed visual outcomes were those that matter the most to them. We explored comparing average vision across treatment groups, but their preferred outcome was the chance of a meaningful vision gain. People can thereby balance any improved chance of vision gain with the downsides of eye surgery.

We undertook a bracketing exercise to determine what improvement in the chance of meaningful vision gain is needed to consider undergoing surgery. This determined the study size (details in ‘Statistical methods’ section). The TIGER visits/procedures are similar to TAPAS, which they found acceptable. PROMS include validated tests of visual function, quality of life and mental wellbeing.

INVOLVE provided costings from successful bids to benchmark our own, alongside their Involvement Cost Calculator and Budgeting for Involvement guide. A PPI expert critiqued our bid.

The patient focus group will review the Patient Information Sheets, serve as a ‘sounding board’ for PPI issues, comment on how best to communicate and disseminate the results, and help us reflect on the PPI process itself.

The Macular Society reviewed the bid, will review the patient literature, raise awareness amongst potential recruits and disseminate results through their website and newsletter, and sit on the TSC.

A study website will provide lay information for participants and their family members. 

#### Health economic analysis

Funding has been secured to undertake a health economic analysis in the UK. Further country-level analysis can be conducted subjected to additional local funding and approval by the study team. Data are collected to enable an analysis in each participating country, subject to additional funding. To ensure consistency of approach, the study health economist will, in addition, be available to advise and support at country level any local analysis of data on cost-effectiveness or budget impact, depending on availability of local funding that can be mustered for such analysis. In this way, we will at a TIGER study level, take into account differences at a country level in prices, medical practice and health care system arrangements across Europe Relevant health economic data collection includes VFQ-25, EQ-5D-5L and SWEMWBS questionnaires as shown in Table 1, and resource utilisation at each follow-up visit except day 1 and week 1 after surgery (this information will be collected in both arms at month 1). Primary economic evaluation will be conducted in a UK context using cost and outcome data for UK participants in the TIGER study. The UK analysis asks, ‘How cost effective is vitrectomy, subretinal TPA and intravitreal gas for SMH secondary to exudative AMD as compared with standard care (intravitreal 2 mg aflibercept monthly for three doses, then 2-monthly until month 12, given to both the surgical and control arms)?’

Untreated, SMH typically has a very severe impact on vision, but it is anticipated that surgery plus aflibercept will improve the outcome. However, it is not known if surgery plus aflibercept will improve vision more than aflibercept alone, and if they do, if the added costs and complications of surgery will offset that added benefit.

The primary outcome of this trial is a gain of at least 10 ETDRS letters of BCVA. Visual acuity is a clinically accepted outcome measure that influences quality of life, and 10 letters exceed the minimum clinically important difference. For these reasons, the primary economic analysis is the incremental cost of surgery plus aflibercept achieving a 10-letter gain in BCVA, as compared with aflibercept monotherapy.

Firstly, from an NHS perspective, we will compare treatment costs and service use with BCVA at 12 months, accounting for the need of further intervention. We will calculate QALYs at 12 months. We will use STATA to undertake a cost-utility analysis. We will undertake a complete case analysis for the in-trial period of 12 months. In addition, we will use multiple imputation using chain equation methods for missing health utility data in a secondary analysis.

##### Measuring intervention costs

We will use national unit costs 2023 and make use of any relevant costing analysis in the literature to cost treatment paths of the two trial groups. Service use information will be collected as part of the eCRF at each data collection point. We will make use of the DIRUM database in the design of this instrument.

##### EQ-5D-5L with vision bolt-on question

To calculate QALYs, participants will complete the EQ-5D-5L with vision bolt-on question at baseline, month 6 and month 12. We chose this version with 5 levels anticipating that it may be more sensitive than the 3 L version in this population, potentially avoiding ceiling effects. EQ-5D-5L is a validated generic, health-related, preference-based measure comprising five domains: mobility; self-care; usual activities; pain and discomfort; anxiety and depression. Each domain has five levels. The questions are complemented by a visual analogue scale, with 0 representing the worst imaginable health and 100 representing the best imaginable health. A cost-utility analysis using EQ-5D-5L (with the vision bolt-on) questionnaire will generate mean cost per QALY estimates, using the area under the curve method, and bootstrapped confidence intervals (5000 replications). We will produce cost-effectiveness planes and cost-effectiveness acceptability curves. We will undertake sensitivity analyses to explore how sensitive results are to any assumptions in our analysis.

We have also included the Warwick-Edinburgh Mental Wellbeing scale short form 7-item questionnaire to assess the well-being of patients.

We will adhere to CHEERS standards for the reporting of economic evaluation studies.

##### Economic budget impact model

An economic budget impact model will be developed to determine the budget implications and any expected cost savings. Guided by good practice recommendations (ISPOR 2012), usually used with the introduction of new pharmaceutical products, these guidelines are equally useful for the development of a budget impact model for the use of surgery to treat SMH. We will model the budget impact. We will undertake sensitivity analysis, varying relevant assumptions, i.e. alternative scenarios. We will validate the model in terms of face validity with ophthalmic surgeons. We will populate the model with data from TIGER and the literature. We will use a budget impact cost calculator approach (ISPOR 2012).

#### Image analysis

The Network of Ophthalmic Reading Centres UK (NetwORC UK), a network of three Ophthalmic Image Reading Centres in the United Kingdom (Belfast, Moorfields Eye Hospital in London, and Liverpool), will be responsible for image analysis (https://www.networcuk.com/). NetwORC UK will provide all training materials for image acquisition and will support sites throughout the trial. Image submission will be via a safe online submission system. All graders involved in TIGER are trained and certified for grading AMD at clinical trials level and will have passed their study-specific certification for TIGER before grading commences.

For TIGER, there will be multimodal grading performed using all imaging modalities to enable the grader to provide a grade for relevant AMD-related abnormalities such as haemorrhage, atrophy and fibrosis in the study eye, and an overall AMD-phenotype decision in the study and fellow eye. All data will be entered into the TIGER database by certified NetwORC UK personnel. Quality assurance and quality control will be conducted according to NetwORC UK protocols and will be reported on to the study team and in the final report.

## Discussion

Several treatments of SMH secondary to wet AMD have been explored, and choosing the most appropriate treatments to compare is a complex decision [7].

TIGER compares vitrectomy, TPA, gas and anti-VEGF therapy anti-VEGF monotherapy. As detailed in the ‘Explanation for the choice of comparators {6b}’ section above, this approach allows us to compare the treatment found to produce the greatest gain in BCVA (surgery), with one that is more easily and speedily delivered, and produced a better mean final BCVA, albeit in patients who probably had less severe presenting disease.

An alternative option would be to compare the delivery of TPA and gas via surgery versus intravitreal injection routes; however, these treatment approaches might be anticipated to produce fairly similar results. If results are relatively similar, then it will be hard to recruit to a large and sufficiently powered study, given that SMH is rare. Such a trial may yet be possible depending on the results of the STAR study. STAR compared vitrectomy, air, TPA and anti-VEGF therapy versus an intravitreal injection of gas, TPA and anti-VEGF therapy (ClinicalTrials.gov Identifier: NCT02557451). When the magnitude of any difference between groups is known, the size of the required trial will be easier to predict.

Another key question relates to the inclusion, or not, of phakic eyes. As discussed above in ‘Relevant concomitant care permitted or prohibited during the trial {11d}’ section above, vitrectomy is known to cause cataract and that may reduce the BCVA primary outcome. Conversely, removal of pre-existing lens opacity may lift BCVA. TIGER aims to produce generalisable results, and accordingly it allows sites to perform cataract surgery alongside vitrectomy, or subsequently, based on patient and clinician preference, the clinical particulars or each participant, and local policy. A more mechanistic approach would be to only enrol pseudophakic eyes, wherein the effect of lens opacity will not confound the analysis of vitrectomy. However, we anticipate that about half of participants will be phakic and excluding such a large group will dent recruitment and mean the results will not be generalisable to a large proportion of patients. Instead, we aim for a more pragmatic trial design, wherein we consider the treatment pathway to include cataract surgery if and as necessary. This is more likely to reflect any post-trial adoption and enhance the utility of the results.

Strengths of this study include a large number of sites across several countries, an RCT design and masked observers for the primary and most secondary outcomes. Weaknesses include an absence of masking in participants, but this is inherent to studies of vitrectomy, as there is no suitable masking. The study size is suitably powered to detect a difference of treatment success of 27% vs 50%, which our PPI exercise indicated was the difference needed to justify surgery, but 210 participants will not be sufficient to rule out a smaller magnitude of difference that some patients might deem sufficient to consider surgery. A much larger study may be difficult to achieve due to the rarity of SMH. The trial aims to start recruitment in April 2021 but the impact of the COVID pandemic is hard to predict and is likely to cause delay.

Assuming the study is sufficiently powered, the clinical impact will largely depend on the results of the study:

- ‘Positive result’ (surgery superior to anti-VEGF monotherapy at the defined target difference): A 210-participant, pan-European RCT will provide robust evidence to guide clinical practice. Patients who would otherwise receive only anti-VEGF monotherapy may be offered surgery instead, with a better visual outcome. The large number of recruiting sites and Key Opinion Leaders involved in this RCT should facilitate adoption, and through local and national guidance.

- ‘Negative result’ (surgery not superior to anti-VEGF monotherapy at the defined target difference): Patients may be spared the risk, discomfort and inconvenience of surgery, and healthcare providers may be spared the expense of surgery. Also, if TIGER demonstrates the safety and efficacy of anti-VEGF monotherapy in SMH, then this may alter any guidelines that currently deny patients with SMH access to anti-VEGF therapy. For example, some guidelines preclude the use of anti-VEGF therapy if BCVA is below 6/96, but this trial might show that is not appropriate for patients with SMH [28, 29].

Adoption would be relatively easy, as TPA is already widely available as a licensed treatment for stroke, myocardial infarction and pulmonary embolus. Vitrectomy, gas and aflibercept are standard eye interventions for a range of vitreoretinal diseases. Hence there are few barriers to adoption. Boehringer-Ingelheim (the TPA manufacturer) could seek a label expansion to include SMH, but meanwhile we expect vitreoretinal surgeons will anyway consider this intervention, as off-label treatments are already commonplace in retinal surgery.

## Trial status

Recruitment aims to commence in April 2021, and the trial is estimated to complete by December 2025, but delay is likely due to COVID pandemic.

## Supplementary Information

**Additional file 1.** Appendix 1: Visual Acuity and Reading Vision Protocol.

**Additional file 2.** Appendix 2: Visual Field Testing Protocol.

**Additional file 3.** Appendix 3: King’s Health Partner’s Clinical Trials Office Monitoring Standard Operating Procedures.

**Additional file 4.** Appendix 4: Study Master Protocol.

**Additional file 5.** Appendix 5: Trial Steering Committee Charter.

**Additional file 6.** Appendix 6: Data Monitoring and Ethics Committee Charter.

**Additional file 7.** Appendix 7: Patient Informed Consent Form.

**Additional file 8.** Appendix 8: Patient Information Sheet.

**Additional file 9.** Appendix 9: Ethical Approval Documentation.

**Additional file 10.** Appendix 10: Funding Letter of Support.

**Additional file 11.** Appendix 11: SPIRIT Checklist.

**Additional file 12.** Appendix 12: WHO Trial Registration Dataset Checklist.

## Abbreviations

AE: Adverse event
AMD: Age-related macular degeneration
BCVA: Best-corrected visual acuity
BEAVRS: British and Eire Association of Vitreoretinal Surgeons
CHEERS: Consolidated Health Economic Evaluation Reporting Standards
CI: Chief Investigator
CNV: Choroidal neovascularisation
CRF: Case report form
CTIMP: Clinical Trial of an Investigational Medicinal Product
DIRUM: Database of Instruments for Resource Use Measurement
DMEC: Data Monitoring and Ethics Committee
eCRF: Electronic case report form
EQ-5D-5L: EuroQol-5D questionnaire with 5-item vision bolt-on
ERM: Epiretinal membrane
ETDRS: Early Treatment Diabetic Retinopathy Study
EVRS: European vitreoretinal society
FAF: Fundus autofluorescence
ICG: Indocyanine green
ILM: Internal limiting membrane
IMP: Investigational Medicinal Product
INR: International normalised ratio
IPCV: Idiopathic polypoidal choroidal vasculopathy
IRB: Institutional Review Board
ISPOR: International Society for Pharmacoeconomics and Outcomes Research
KCL: King’s College London
KHP-CTO: King’s Health Partners’ Clinical Trials Office
logMAR: Logarithm of the minimum angle of resolution
logRAD: Log reading acuity determination
MAR: Missing at random
MedDRA: Medical Dictionary for Regulatory Activities
MNAR: Missing not at random
NEI: National Eye Institute (USA)
NetwORC UK: The Network of Ophthalmic Reading Centres UK
NHS: National Health Service (UK)
NIHR: National Institute for Health Research (UK)
nIMP: Non-Investigational Medicinal Product
OCT: Optical coherence tomography
PDT: Photodynamic therapy
PI: Principal Investigator
PPI: Patient and Public involvement
PROM: Patient-reported outcome measure
QALY: Quality-adjusted life year
RAP: Retinal angiomatous proliferation
RCT: Randomised controlled trial
REC: Research Ethics Committee
RPE: Retinal pigment epithelium
SAE: Serious adverse event
SD-OCT: Spectral domain optical coherence tomography
SF_6_: Sulfahexafluoride
SI: Sub-Investigator
SMH: Submacular haemorrhage
SmPC: Summary of Product Characteristics
SOP: Standard operating procedure
SOSU: Scottish Ophthalmological Surveillance Unit
SWEMWBS: Short Warwick-Edinburgh Mental Well-being Scale
TIGER: Vitrectomy, subretinal Tissue plasminogen activator and Intravitreal Gas for submacular haemorrhage secondary to Exudative Age-Related macular degeneration
TPA: Tissue plasminogen activator
TSC: Trial Steering Committee
UK: United Kingdom
VA: Visual acuity
VEGF: Vascular endothelial growth factor
VFQ-25: 25-item Visual Function Questionnaire
