# Supplementary material for: Vitrectomy, subretinal Tissue plasminogen activator and Intravitreal Gas for submacular haemorrhage secondary to Exudative Age-Related macular degeneration (TIGER): study protocol for a phase 3, pan-European, two-group, non-commercial, active-control, observer-masked, superiority, randomised controlled surgical trial
Source: Trials. 2022 Jan 31;23:99. doi: 10.1186/s13063-021-05966-3 (PMC8805308; doi:10.1186/s13063-021-05966-3)

# Appendix 1: Testing of Distance Visual Acuity and Radner Reading Speed

Study eye distance best-corrected visual acuity (BCVA) at baseline, month 6 and month 12 form the basis of the primary outcome and key secondary outcomes. Reading speed is an another important, patient-centred secondary outcome.

Participants require a fully refracted BCVA at baseline, month 6 and 12. Radner reading speed is also completed at baseline, month 6 and 12.

For the other visits a ‘clinic ETDRS VA’ is measured. This uses the participant’s distance spectacles, if they wear spectacles (or their previous trial refraction if their spectacles are not available and they require a spectacle correction), with and without pinhole, but is otherwise as per the site’s usual VA methodology.

The schedule of full refracted ETDRS BCVA and reading vision assessments is shown in the table below. Measurements are only required from the study eye, except for the fully refracted BCVA at baseline and month 12, and reading vision at month 12, when they should be measured in both eyes.

| **Visit** | Screening/  Baseline | D1 | W1 | M1 | M2 | M4 | M6 | M8 | M10 | M12 |
| --- | --- | --- | --- | --- | --- | --- | --- | --- | --- | --- |
| Clinic ETDRS VA in study eye |  | Arm A | Arm A | X | X | X |  | X | X |  |
| Full refracted ETDRS BCVA | Both eyes |  |  |  |  |  | Study eye only |  |  | Both eyes |
| Radner reading vision | Study eye only |  |  |  |  |  | Study eye only |  |  | Both eyes |

D1=day 1; W1=week 1; M2=month 2

Visual acuity testing and reading vision will be undertaken by **masked assessors**, who are unaware of the participant’s treatment arm. They should not have access to prior VA measurements but can review prior refractions. Staff involved in TIGER should instruct participants not to reveal which arm of the trial they are in to VA assessors.

## Distance Visual Acuity

Trial BCVA measurements (Baseline, Month 6 and Month 12) will be performed by trial certified vision examiners, in trial certified rooms, and with trial certified equipment. The **name** of the vision examiner should be documented in the participant’s **source document** at each visit. BCVA examiners are “masked” to trial assignment and previous BCVA testing results. Therefore, BCVA examiners should not have access to the participant’s chart or previous BCVA testing results. Only the previous refraction should be made available. Refraction should be rechecked, and updated if necessary, prior to VA testing at Baseline, Month 6 and Month 12.

For BCVA measurement at intervening visits, see notes above.

**Equipment**

Refraction equipment required includes:

1. Retroilluminated Light box and ETDRS 4 meter distance acuity chart set
2. Trial lens frames
3. Trial lens set with plus or minus cylinder lenses
4. Jackson cross-cylinders of 0.25, 0.50, and 1.00 diopters
5. Pinhole occluder
6. Tissues or eye pads and tape
7. A 1 meter rigid measuring stick

***Visual Acuity Charts***

Chart 1 is used for testing the VA of the RIGHT eye; Chart 2 for testing the LEFT eye; and Chart R (or 3) for refraction only. Patients should not be allowed to see any of the charts before the examination.

***Visual Acuity Lane and Visual Acuity Box***

A distance of **4 metres** is required between the patient’s eyes and the VA chart. With the box light off, not more than **15 foot-candles of light** (161.4 Lux) should fall on the centre of the chart. To measure the amount of light, the room is set up for VA testing, but with the box light off. The light metre is placed at the fourth line from the top of the chart, with its back against the chart and the reading is taken. If more than one lane is available for testing VA, the VA of an individual patient should be measured in the same lane at each visit, if possible. If different lanes are used to test VA, they must each meet the same standards.

Retro illuminated ETDRS charts are used in this trial. The illuminator box will be either wall-mounted or mounted on a stand. The light box should be mounted at a height such that top of third row letter is 49 ± 2 inches from floor.

The VA light box is equipped with two General Electric 20-watt fluorescent tubes (or equivalent lightbox housing 24 Watt fluorescent tubes) and ballast. Each tube is partly covered by a 12 or14-inch fenestrated sleeve, which is centered on the tube and open in the back. This serves as a “baffle” to produce even illumination over the testing chart. Because the illumination of fluorescent tubes diminishes by 5 percent during the first 100 hours and by another 5 percent during the next 2000 hours, new tubes should be kept on for a total time period of 4 days (96 hours) before use in the study, and should be replaced once a year. Luminance will be confirmed with a use of a light metre (Sekonik L-398A) at the outset of the trial to confirm a minimum luminance of 85 cd/m2 (80-160 cd/m2).

A **sticker** should be placed on the back of the light box, indicating the date on which the present tubes were installed. A spare set of burned in bulbs should be available on site.

***Beginning Approximate Refraction***

At the Baseline visit, the patient’s beginning refraction is determined by one of the following ways:

If the patient’s VA is 6/30 (20/100) or better and the patient does not require glasses for distance vision, then the beginning approximate refraction should be no lens correction or plano.

If the patient’s VA is 6/30 (20/100) or better and the patient requires glasses for distance viewing, the glasses should be measured using a focimeter, and these measurements are used for the beginning refraction

If the patient’s VA is less than 6/30 (20/100) with or without correction, then retinoscopy or autorefraction should be performed to determine the beginning approximate refraction.

If the patient wears contact lenses for refraction, a notation should be made that the refraction was over contact lenses. It is suggested that the patient wear the contact lenses for future examinations. If the patient is not a regular contact lens wearer and wore the lenses by mistake, they should be removed and you should wait at least 30 minutes before beginning the refraction. The patient should be reminded not to wear contact lenses at subsequent visits.

Refractions are performed with either plus or minus cylinder power. Whichever cylinder type is used at baseline (minus or plus) must be used for all subsequent visits. Best correction results should be recorded on the sponsor provided worksheet which will be included in the source documents. **At each follow-up visit, the results of the protocol refraction from the previous visit are used as the beginning approximate refraction**. If the previous refraction is not available for some reason, the procedure described immediately above should be used. Whilst previous refraction results are made available at subsequent visits, **previous VA results should not be visible to the examiners at subsequent testing, so that *assessment of VA is masked to prior visual function (and to treatment arm)*.**

The charts used for measuring distance VA must NOT be used for refraction. Refraction for each eye should be performed at 4 metres unless the patient’s VA measured at **4 metres** on the refraction chart (Chart R or Chart 3) **is worse than 6/48 (20/160). If VA is worse than 6/48 (20/160) the eye is refracted at 1.0 metre.** If during the refraction process at one metre, the patient is reading letters on the eighth line or lower line of the chart, the refraction should continue at 4 metres. Whenever a patient cannot read any letters on the top line of Chart R or Chart 3 at 1.0 metre the vision should be checked with a pinhole to see whether reduced vision is due, at least in part, to a larger refractive error, however, be aware that submacular haemorrhage may produce a central scotoma that negates the refractive benefit of a pinhole.

***Patient Refraction***

Patient refraction allows one to determine the best correction for a patient to perform the VA tests. The “push plus” approach is used. Add minus dioptre spherical corrections only when the patient is able to read at least one more letter on a line or a letter on a smaller line.

Procedure

1. Measure and record the distance vision of the eye being tested using Chart R while occluding the fellow eye. The fellow eye should be lightly patched with an eye pad or tissue and tape. Patients should be reminded to blink and encouraged to use eccentric fixation, or their side vision, when necessary.
2. All refraction and vision testing must be done at 4 metres or 1 metre. Distance for 4 metres is 13 feet and 1.5 inches or 157.5 inches. The 1 metre distance is 39 and 3/8 inches.
3. All patients should be seated for testing. A rigid measuring device should be used to measure the distance from the patient to the chart if testing is done at 1 metre. The distance is measured from the outer canthus to the center of the second letter (left eye) or fourth letter (right eye) of the third line of the chart. For 4 metre testing, clear and permanent floor markings should be used to mark the distance for consistency.
4. Place and adjust the trial frame on the patient’s face so that the lens cells are parallel to the anterior plane of the orbits and centered in front of the pupils. Adjust the lens cells for the proper distance from the cornea. Be sure the trial frame is comfortable on the patient’s face.
5. Occlude the left eye by lightly patching with an eye pad or tissue and tape. Place the spherical lens correction in the compartment closest to the eye. The cylindrical lens correction, if present, is placed in the compartment in front of the spherical correction. Adjust the axis.
6. Spherical Correction: To determine the highest plus or least minus sphere, refract the right eye. The following refraction steps are recommended for VAs of 6/3 (20/10) to 6/24 (20/80) with the beginning approximate refraction. For VAs less than 6/24 (20/80), refer to the refraction table for the appropriate sphere and cylinder powers and testing distance (see summary below) and follow a similar procedure. Note: Whenever VA is improved to a higher range, refraction should be performed with the smaller sphere and cylinder powers given for the better VA level (See table at end of appendix).
7. Hold a +0.50 sphere in front of the patient’s right eye. The patient should be looking at the smallest legible line on the VA chart. In these exact words, ask the patient, “Is this better, worse, or no change?”
8. If the patient responds that the vision is worse or blurred, remove the +0.50 sphere from in front of the trial frame and go to Step 6d.
9. If the patient responds better or no change, remove the +0.50 sphere from in front of the trial frame and replace the spherical lens in the trial frame with a spherical lens that is one-half dioptre more positive. Continue this procedure by returning to Step 6a and repeating this process until a +0.50 makes the vision worse or blurred and then go to Step 6d.
10. Hold a -0.50 sphere in front of the patient’s right eye. In these exact words, ask the patient, “Is this better, worse or no change?” If the patient replies “worse” or “no change”, go to Step 6f. If they reply “better” go to step 6e.
11. Hold the -0.50 sphere in front of the eye. If the patient responds that the vision is better, ask the patient to read the VA chart. Only when the VA is improved, by at least one letter, may you increase the minus by 0.50 (or decrease the plus) and repeat Step 6d. Whenever VA is not improved, go to Step 6f.
12. Remove the -0.50 sphere from in front of the eye and hold a +0.50 sphere in front of the right eye. In these exact words, ask the patient, “Is this better, worse, or no change?” If the patient responds that vision is better or unchanged, then return to Step 6c. Otherwise, go to Step 7. Spherical testing should always end with a plus lens.
13. Cylinder Axis: To determine and refine the cylinder axis for PLUS cylinder, proceed as follows; (If minus cylinders are used, the appropriate technique using minus cylinders must be employed and minus cylinder must be used throughout the trial.)
14. Have the patient look at a line which is either one or two lines larger than the smallest line the patient is able to read. Ask the patient to focus on a rounded letter such as “C”, “D”, or “O”. The patient should focus on this same letter throughout this procedure.
15. If a cylinder is present in the beginning approximate refraction, then go to Step 7c. Otherwise, follow the option listed below to determine if cylinder may be needed.

Testing for cylinder when there is none in the beginning approximate refraction:

Place a +0.50 dioptre cylinder with the positive axis first at 90°, then compare this to no cylinder; repeat this procedure for 180°, then 45°, and 135° always comparing to no cylinder after each axis position. If the patient says that vision is improved at any one of the four axis positions, place a +0.50 cylindrical lens in the trial frame at the preferred axis and go to step 7c. If the patient prefers no cylinder at all four axis positions, then go to Step 9.

1. Place the +0.25 dioptre hand held cross-cylinder (for VA 6/3 – 6/24; 20/10-20/80) first with the positive axis 45° to the right of the preferred cylinder axis (as determined above), and second with the positive axis 45° to the left of the preferred cylinder axis. Ask the patient, “Which do you like better, position one or position two?” Also, tell the patient that both positions may blur their vision. The patient must choose the least blurred position, either one or two. “Neither” is allowed only if both positions are equally blurred or equally good.
2. If “neither” position is better and this was the first test of axis position, move the axis of the cylinder in the trial frame 15° to the right or left and return to Step 7c. Otherwise, proceed to Step 7e.
3. When one position is preferred over another, move the cylinder to the preferred positive axis position in the step sizes noted below and return to Step 7c. If no single position is better than another than go to Step 8.

| **Cylinder Refinement: *suggested* axis step sizes** | | | |  |
| --- | --- | --- | --- | --- |
| Cylinder Power |  | Axis Step Sizes | |  |
| <1.00D | |  | 15^o^ | |
| 1.00 to <2.00D | |  | 10 ^o^ | |
| 2.00 to <3.00D | |  | 5 ^o^ | |
| 3.00 to<5.00D | |  | 3 ^o^ | |
| 5.00 to<8.00D | |  | 2 ^o^ | |

1. **Cylinder Power**: Cylinder power is refined by following the steps:
2. Ask the patient to look at the **smallest line** that can be read on the VA chart.
3. Test the cylinder power by placing the 0.25 dioptre cross-cylinder (for vision of 6/3 - 6/24; 20/10-20/80) first with the positive axis and second with the negative axis coincident with the cylinder axis. Ask the patient, “Which is better, position one or position two?” Do not give the patient the choice of neither.
4. If the patient prefers the minus axis coincident with the cylinder axis, the total power of the correcting plus cylinder is reduced by 0.25 dioptre. Repeat the process until the patient cannot choose one of the cross cylinder positions over the other. If the patient indicates a change that would introduce negative cylinder power, remove all cylinder power and continue testing for positive cylinder power at an axis 90°away from the previous axis. Otherwise go to Step 8d.
5. If the patient prefers the plus axis coincident with the cylinder axis, increase the power of the cylinder by 0.25 dioptres and return to Step 8b. Otherwise proceed to Step 8e.
6. When the patient feels that both positions are equally bad or good, and the cylinder power in the trial frame has changed by more than 0.50 dioptre, return to Step 7c and re-check the axis if necessary. Otherwise, proceed to Step 9.

*Note: If the cylinder is changed by more than 0.50 dioptre, the* ***spherical equivalent*** *should be maintained. (For each 0.50* ***plus*** *CX increase, add –0.25 to the sphere, for each 0.50* ***minus*** *CX increase, add +0.25 to the sphere).*

1. **Spherical Correction** ***Refinement****:* Recheck, or ***“refine”*** the power of the sphere by adding **+0.25 and -0.25** spheres and changing the spherical power by 0.25 dioptre increments of the appropriate sign until the patient cannot detect any improvement in vision. As a reminder, **minus sphere should only be added if the patient can read additional letters** and spherical testing should always begin and end with a plus lens.
2. Record the lens corrections obtained by patient refraction for the right eye on the examination form in the section for VA measurements as the corrections obtained by protocol refraction for the right eye.
3. Repeat the entire process (Steps 1-10) for the left eye and record the refraction result on the VAE worksheet.
4. As noted above, bilateral fully refracted BCVA is only required at baseline and month 12. At month 6 (and other visits) only the study eye needs to be tested. Refraction should be updated at baseline, and months 6 and 12. For intervening ETDRS BCVA measurements use the previously determined refraction.

***Best-Corrected Visual Acuity Measures***

As a reminder, Charts 1, 2, and R (or 3) are used for testing the right eye, left eye, and refraction, respectively. Patients should not see the charts until the test begins. The lens correction from the patient refraction should be in the trial frame worn by the patient. All eyes must be tested at 4 metres first, even if the refraction was performed at 1 metre.

The patient should be seated comfortably directly in front of the chart so that the eyes remain at the 4 metre distance. For bilateral testing, always begins with the right eye. The fellow should be occluded with a folded tissue or eye pad lightly taped over the eye behind the trial frame serves as an effective occluder that allows eccentric fixation without inadvertent use of the covered eye. After testing the right eye, occlusion of the right eye should be done BEFORE Chart 2 is put up for testing the left eye.

The patient is asked to read the letters slowly, approximately one letter per second. The patient should be told that only one chance is given to read each letter, but they may change their mind before moving to the next letter. If the patient is unsure about the identity of the letter, then the patient should be encouraged to guess.

The patient should begin by reading the top line of the chart and continue reading every letter on each smaller line, from left to right on each line. The patient should be encouraged to continue reading even if making mistakes. Each letter read is counted. The examiner circles every correct letter read and totals each line and the whole column (0 if no letters are correct) on the data collection form. An X is put through letters read incorrectly. Letters, for which no guess was attempted, are not marked. When a patient reaches a level where he/she cannot guess, the examiner may stop the test provided that the patient has made errors on previous guesses, which is a clear indication that the best VA has been obtained.

When a patient cannot read at least 20 letters on the chart at 4.0 metres, the patient is tested at 1.0 metre. The distance from the patient to the chart should be measured again using the rigid 1 metre stick. The distance is measured from the outer canthus to the center of the fourth letter (right eye) or the second letter (left eye) of the third line of the chart. The spherical correction in the trial frame should be changed by adding +0.75 to correct for the closer test distance. The patient may fixate eccentrically or turn or shake his/her head to improve VA. Particular care should be taken to make sure the patient does not move forward when testing at 1 metre. The patient should be reminded to blink.

The examiner should not tell the patient if a letter was identified correctly. The patient may be encouraged by neutral comments, such as “good”, “next”, and “OK”. The examiner should not stand close to the chart during testing. Attention should be focused on the patient and the data collection form. If the patient has difficulty locating the next line to read, the examiner may go up to the chart and point briefly to the next line to be read, but then must move away from the chart.

When 20 or more letters are read at 4 metres the VA score for that eye is recorded as the number of letters correct plus 30 (refer to the VA worksheet) The patient gets credit for the 30 1M letters even though they did not have to read them. Otherwise, the VA score is the number of letters read correctly at 1.0 metre plus the number, if any, read at 4M. If no letters are read correctly at either 4.0 metres or 1 metre, then the VA score is recorded as 0.

***Testing for Count Fingers Vision, Hand Motion Vision and Light Perception/No Light Perception (NLP) Vision***

If the patient’s VA is so poor that he/she cannot read any chart letters when tested at 1 metre then the patient’s ability to count fingers, detect hand motion, or have light perception should be evaluated.

***Testing for Count Fingers Vision***

In testing for count fingers vision, the examiner’s hand holding 1, 2, or 5 fingers is held steady at a distance of two feet directly in front of the eye being examined. The fellow eye is completely occluded with a patch on the face. A light should be shown directly on the hand from behind the patient. The examiner’s fingers should be presented in random order and repeated 5 times. Eccentric fixation, if present, should be encouraged. If the patient correctly identifies three of the five presentations, then count fingers vision is noted. If not, then the patient must be tested for hand motion vision.

***Testing for Hand Motion Vision***

The examiner’s hand with all fingers spread out should be extended two feet directly in front of the eye being examined. The fellow eye should be occluded with a patch on the patient’s face. A light should be shone directly on the examiner’s hand from behind the patient. The examiner’s hand should be moved in an up-and-down direction (vertically) or in a side-to-side direction (horizontally) at a constant speed of approximately one back and forth presentation per second. The patient is instructed that the examiner’s hand will be presented and they will have to respond to the question: “What am I doing with my hand?” This should be repeated five times. Three out of five correct responses indicate that hand motion vision is present. If the patient does not correctly identify three of five presentations, then you must test for light perception.

***Testing for Light Perception/No Light Perception Vision***

Light perception should be tested with an indirect ophthalmoscope in a darkened room. The fellow eye should be completely patched and also covered by the patient’s hand. The indirect ophthalmoscope light should be in focus at 1 metre with the rheostat set at maximum voltage. From that distance the beam should be directed in and out of the patient’s eye at least four times, and the patient should be asked to respond when he or she sees the light. If the examiner is convinced that the patient perceives the light, vision should be recorded as “light perception”, if not, vision should be recorded as “no light perception”.

| 4M Refraction Protocol Summary | | | | | | | |
| --- | --- | --- | --- | --- | --- | --- | --- |
| **Refraction Distance** | **Check Sphere First** | | **Check Cylinder Axis then Power** | | | **Sphere “Refinement”** | |
| **If VA on “R” chart is between:** | **Power**  **(a)** | **Increment** | **Axis**  **(b)** | **Power**  **(c)** | **Increment** | **Power**  **(d)** | **Increment** |
| **6/3 -6/24**  **20/10 - 20/80**  **(4 m)** | **+.50**  **-.50** | **+.50**  **-.50** | **.25**  **JCC** | **.25**  **JCC** | **+.25**  **-.25** | **+.25**  **-.25** | **+.25**  **-.25** |
| **6/30 – 6/48**  **20/100 - 20/160**  **(4 m)** | **+1.00**  **-1.00** | **+1.00**  **-1.00** | **.50JCC** | **.50**  **JCC** | **+.50**  **-.50** | **+.50**  **-.50** | **+.50**  **-.50** |
| **6/60 -6/120**  **20/200 - 20/400**  **(1 m)** | **+2.00**  **-2.00** | **+2.00**  **-2.00** | **1.00**  **JCC** | **1.00**  **JCC** | **+1.00**  **-1.00** | **+1.00**  **-1.00** | **+1.00**  **-1.00** |
| **<6/120**  **<20/400**   1. **m)**   sequence refraction a-d | **+2.00**  **-2.00** | **+2.00**  **-2.00** | **No cylinder test required** | | | **No refinement required** | |

## Radner Reading Chart

The "Radner Reading Charts" have been developed on the basis of the concept of "sentence optotypes" for the examination reading acuity and speed.^17^ Print sizes are geometrically (logarithmically) scaled. Reading acuity is given in logRAD (logReading-Acuity-Determination) to permit statistical analysis, and the results obtained can be compared to other logarithmically scaled vision systems (e.g. logMAR).^18^ Reading speed is analyzed in words per minute (wpm).

To guarantee accurate, reproducible and standardized measurements of reading speed and reading acuity, "sentence optotypes" have been created to minimize the variations between the test items. Through interdisciplinary cooperation, a series of test sentences were developed that are highly comparable in terms of the number of words (14 words), as well as the word length, number of syllables, position of words, lexical difficulty and syntactical complexity. The most similar sentences were statistically selected for the Radner Reading Charts. The Radner Reading Charts have then been statistically evaluated in terms of test-retest reliability, inter-chart reliability and a variance component analysis.^17^

**Aim**

In TIGER, the Radner maximum reading speed is an important secondary outcome, but two additional measurements will be acquired alongside this:

- Reading acuity (unit, logRAD, in 0.1 log steps): Equivalent to logMAR distance acuity. Corresponds to the smallest sentence optotype read accurately in less than 30 seconds.
- Maximum reading speed (words per minute): Fastest, accurate reading speed achievable at any sentence optotype.
- Mean reading speed (words per minute): Average of the fastest 3 sentences optotypes read accurately, with optotype size from 0.9 logRAD to 0.3 logRAD. Omit if fewer than 2 sentence optotypes were read accurately.

**Instructions**

Testing should be undertaken in the study eye only, except at the final visit, where both eyes are tested separately. When testing both eyes, the study eye should be tested first. Test reading vision after the measurement of ETDRS distance BCVA and before visual field testing. Record timings with a digital stopwatch.

**Near correction, test distance, and illumination**

The refraction used for testing ETDRS vision at 4 m should be altered for reading vision testing, which is performed at 25 cm, by providing a 4.0 Dioptre ADD. Verify the distance regularly throughout testing, with a ruler.

At baseline record the level of illumination measured immediately in front of the middle of the reading chart (acceptable range: 100 -110 cd/m2 or 100-110 Lux), and ensure the same level of illumination and testing conditions are used for testing at Month 6 and 12.

**Technique**

a) The reading chart is held by the patient; the sentences are covered with a piece of paper.

b) Instruct the patient to uncover the chart sentence by sentence and to read only one sentence per measurement. Instruct the patient to uncover the next sentence only when asked to do so - the examiner gives the command to uncover the next sentence.

c) "Please read the sentences aloud as quickly and accurately as possible. Read each sentence to the end, and do not correct reading errors."

d) "Please uncover the first sentence and start reading." Start the measurement with the stopwatch when the patient starts reading, and measure the reading time until the end of the sentence. If possible, aim to improve accuracy by starting the stopwatch with the initial pre-movements of the lips at the vocal onset (pre-phonetic strain).

e) Write the reading time on the scoring sheet next to the sentence read, and record any reading errors by marking them on the sheet (sum up the syllables of words read wrong). If patient’s accents alter the pronunciation of a word, but the meaning is correct (example: different => diff´rent), credit the patient with the full word, and the syllables are counted as given in the word books.

f) Stop criterion: reading time longer than 30 seconds for a sentence or severe errors.

**Calculating Reading Speed**

Reading speed in words per minute (wpm) can be calculated on the basis of the number of words in a sentence (=14) and the time (t = seconds) needed to read the sentence, or using the values in Table 1.

Reading speed (wpm): 14/time in seconds x60 = 840/time in seconds


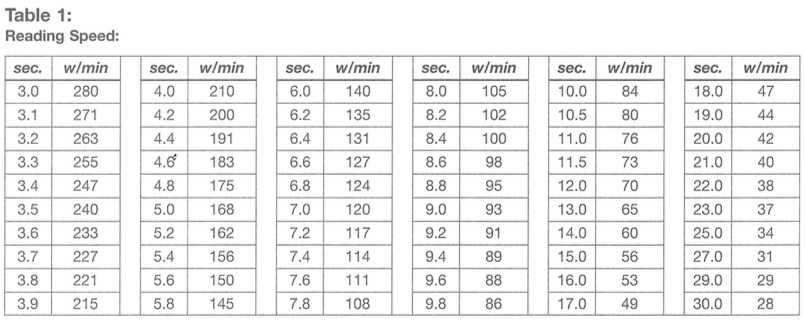

Supplement: Supplementary file 1 — Additional file 1. Appendix 1: Visual Acuity and Reading Vision Protocol. [file 13063_2021_5966_MOESM1_ESM.docx]
