# Supplementary material for: Vitrectomy, subretinal Tissue plasminogen activator and Intravitreal Gas for submacular haemorrhage secondary to Exudative Age-Related macular degeneration (TIGER): study protocol for a phase 3, pan-European, two-group, non-commercial, active-control, observer-masked, superiority, randomised controlled surgical trial
Source: Trials. 2022 Jan 31;23:99. doi: 10.1186/s13063-021-05966-3 (PMC8805308; doi:10.1186/s13063-021-05966-3)
Supplement: Supplementary file 2 — Additional file 2. Appendix 2: Visual Field Testing Protocol. [file 13063_2021_5966_MOESM2_ESM.docx]

# Appendix 2: Visual Field Testing

# Submacular haemorrhage (SMH) may lead to central macular scarring, and consequently a central scotoma in the affected eye. Hence, this study incorporates an assessment of the central visual field at screening, month 6 and month 12.

# Central visual field testing should be completed in the study eye only, by experienced operators, using the Swedish Interactive Threshold Algorithm (SITA) standard 10-2 program, test stimulus size III, and the Zeiss Humphrey Field Analyser 3 (HFA3, Carl Zeiss Meditec, Dublin, USA). If this device is not available please use an equivalent programme from another device, but contact the study team for advice on its suitability beforehand. The same machine should be used for a given participant for each of their visual field tests.

Visual field testing should occur after refraction and ETDRS visual acuity testing, to minimise the impact of any testing fatigue on the primary outcome (visual acuity). This will also provide a same-day distance refractive measurement, which can be used to determine the correct trial lens for visual field testing, as per the Humphrey Field Analyser manual (see sections B-1, B-2, 4-3 and 4-5), available [here](https://www.zeiss.fr/content/dam/Meditec/international/ifu/documents/hfa3/current/2660021166131_a_artwork.pdf), or at:

https://www.zeiss.fr/content/dam/Meditec/international/ifu/documents/hfa3/current/2660021166131_a_artwork.pdf

Reliable visual field testing (<15% false positives and <20% fixation losses) may not be possible in some participants due to poor visual acuity and the operator should use the eye tracker to carefully monitor eye movements and prompt the participant to maintain central fixation as required, throughout the test.

Visual field tests should be scanned and saved as .pdfs. The file name should include the participant’s study ID and visit (eg month 6), but not the participant’s name.

The visual field pdfs should be transferred to the reading centre, as per the colour fundus photographs and angiography images. The address and contact details for the NetwORC UK Reading Centre are available [here](https://www.networcuk.com/), or at:

https://www.networcuk.com/
