# Supplementary material for: Vitrectomy, subretinal Tissue plasminogen activator and Intravitreal Gas for submacular haemorrhage secondary to Exudative Age-Related macular degeneration (TIGER): study protocol for a phase 3, pan-European, two-group, non-commercial, active-control, observer-masked, superiority, randomised controlled surgical trial
Source: Trials. 2022 Jan 31;23:99. doi: 10.1186/s13063-021-05966-3 (PMC8805308; doi:10.1186/s13063-021-05966-3)
Supplement: Supplementary file 3 — Additional file 3. Appendix 3: King’s Health Partner’s Clinical Trials Office Monitoring Standard Operating Procedures. [file 13063_2021_5966_MOESM3_ESM.pdf]

Clinical Trial Monitoring

POLICY DETAILS	
Document Type	Standard Operating Procedure
Document name	KHP-CTO/CT/SOP3.0<br>Clinical Trial Monitoring
Version	Final v 6.1 01/10/2018
Effective from	01 Oct 2018
Review date	09 April 2021
Owner	King's Health Partners Clinical Trials Office
Prepared by	Helen Critchley, Quality Manager
Approved by	Jackie Pullen, Director KHP-CTO
Superseded document	Final v 6 04/04/18
Relevant regulations/legislation/guidelines	Statutory Instrument 2004 no 1031<br>Statutory Instrument 2006 no 1928 *(as amended from time to time)*

CHANGE HISTORY			
Date	Version Number	Change details	Approved by
09/Jun/2010	2.0 Final	Transfer to Kings Health Partner Livery, Update to 2.0 GLOSSARY, 3.0 SCOPE and replacement of "monitor" to CRA throughout text.	Jackie Powell
10/Nov/2010	3.0 Final	Clarification that email is an accepted method of communication.	Jackie Powell
22/Feb/2013	4.0 Final	Update to glossary and procedures to reflect current practice and change of branding from JCTO to KHPCTO.	Jackie Powell
03/Aug/2016	5.0 Final	Update of Glossary and Procedures to reflect current practice.	Jackie Pullen

04/Apr/2018	6.0 Final	Update to include review of the source data location list during routine monitoring visits. Update to glossary terms for REC, Regulations and Reference Safety Information.	Jackie Pullen
01/Oct/2018	6.1 Final	Minor amendment to include trials managed by KHP-CTO	Jackie Pullen

Table of Contents

1.0 GLOSSARY 4

2.0 BACKGROUND AND PURPOSE 6

3.0 SCOPE 7

4.0 PROCEDURE 7

4.1 Selection of Clinical Research Associates 7
4.2 Extent of Monitoring 7
4.3 CRA Responsibilities 8
4.4 Monitoring Report 10

5.0 RELATED TEMPLATES 10

5.1 Monitoring Visit Report Form template 10
5.2 Contact Comment Form template 10
5.3 Note to File template 10
5.4 Monitoring Plan 10

6.0 APPROVAL AND SIGNATURE 10

1.0 GLOSSARY

Adverse Event (AE) - Any untoward medical occurrence in a subject to whom a medicinal product has been administered, including occurrences which are not necessarily caused by or related to that product.

Case Record Form (CRF) - a printed, optical, or electronic document designed to record all of the protocol required information to be reported to the sponsor on each trial subject.

Clinical Trial - Any investigation in human subjects, other than a non-interventional trial intended to discover or verify the clinical, pharmacological or other pharmacodynamic effects of one or more medicinal product or to identify any adverse reactions to one or more such products and to study absorption, distribution metabolism and excretion in one of more such products with the object of ascertaining the safety or efficacy of those products.

Clinical Research Associates (CRAs) – Part of the KHP-CTO Quality Team. Ensure compliance with the Regulations, GCP and SOPs, by monitoring clinical trials.

Curriculum Vitae (CV) - A summary of a person's education, professional history and job qualifications.

Good Clinical Practice (GCP) - as defined in the Regulations.

Health Research Authority (HRA) – Provides single assessment and approval process for clinical research conducted in England.

Informed Consent Form (ICF) – The document which is signed by the participant/legal representative as well as the person who conducted the informed consent discussion confirming the volunteers willingness to participate in the particular trial after having been informed of all aspects of the trial that are relevant to their decision.

Investigator Site File (ISF) - a standard filing system which allows the effective storage and location of essential documents related to an individual trial site.

Investigational Medicinal Products (IMP) - means a pharmaceutical form of an active substance or placebo being tested, or used as a reference in a clinical trial. This includes a medicinal product which has a marketing authorisation but is, for the purposes of the trial -

(a) used or assembled (formulated or packaged) in a way different from the form of the product authorised under the authorisation,
(b) used for an indication not included in the summary of product characteristics under the authorisation for that product, or
(c) used to gain further information about the form of that product as authorised under the authorisation

KHP-CTO Standard Operating Procedures (SOPs) - "detailed, written instructions to achieve uniformity of the performance of a specific function," SOPs are the base on which Quality Systems and Processes are conducted and monitored against.

King's Health Partners Clinical Trials Office (KHP-CTO) – Established in 2006 by Kings College London, Guy's & St Thomas' NHS Foundation Trust and King's College Hospital NHS Foundation Trust to provide a streamlined approach for all aspects of trial administration.

KHP-CTO Quality Team - Comprises the Clinical Quality Manager, Clinical Research Associate(s), Clinical Trial Administrator(s), Systems Executive, Training Executive (s) and Training Assistant.

Monitoring Plan (MP) – A document detailing how all the monitoring activities for the trial will be carried out based upon the trial risk assessment.

MATTS – MedSciNet's Active Trial Tracking System. An electronic Clinical Trial Portfolio Management System.

Monitoring Visit Report (MVR) – A report written by the CRA to the Sponsor (or representative) after each site visit.

Participant Information Sheet (PIS) - explains all relevant trial information to assist the trial participant in understanding the expectations and requirements of participation in a clinical trial.

Quality Control (QC) - The operational techniques and activities undertaken within the quality assurance system to verify that the requirements for quality of the trial-related activities have been fulfilled.

Reference Safety Information (RSI) – a record of known serious adverse reactions to the IMP. Will be contained either within an Investigator Brochure, for non-licensed IMPs, or a Summary of Product Characteristics (SmPC), for IMPs with a marketing authorisation.

Research Ethics Committee (REC) – An independent committee, made up of medical, scientific and lay members, who must approve the intended procedures and documentation of any proposed study. Any study whose participants have been identified through a connection to NHS facilities or services will be allocated to one of 90 NHS RECs located throughout the country. The RECs make their decisions independently but are centrally administered by the HRA.

Research & Development Dept (R&D) – NHS department responsible for confirmation of capacity and capability for all clinical research.

Serious Adverse Event or Reaction (SAE/SAR) - A serious adverse event is defined as an adverse experience that results in any of the following outcomes:-

- death
- a life-threatening adverse experience (any adverse experience that places the patient or subject, in the view of the Investigator, at immediate risk of death from the reaction as it occurred, i.e., it does not include a reaction that, had it occurred in a more severe form, might have caused death)
- inpatient hospitalisation or prolongation of existing hospitalisation
- a persistent or significant disability/incapacity (a substantial disruption of a person's ability to conduct normal life functions)
- a congenital anomaly/birth defect.

Source Documentation - Original documents, data, and records (e.g., hospital records, clinical and office charts, laboratory notes, memoranda, subjects' diaries or evaluation checklists, pharmacy dispensing records, recorded data from automated instruments, copies or

transcriptions certified after verification as being accurate copies, microfiches, photographic negatives, microfilm or magnetic media, X-rays, subject files, and records kept at the pharmacy, at the laboratories and at medico-technical departments involved in the clinical trial).

Summary of Product Characteristics (SmPC) – this reference document is produced for health professionals and details of how to use a medicinal product safely and effectively.

The Regulations – The Medicines for Human Use (Clinical Trials) Regulations are the legal framework governing the conduct of CTIMP research in the UK. The relevant regulations are Statutory Instrument 2004-1031 and its amendments (to date, 2006-1928, 2006-2984, 2008-941, 2009-1164 and 2010-1882). Compliance with the Regulations is inspected and enforced by the MHRA.

Trial Master File (TMF) - a standard filing system which allows the effective storage and location of essential documents, that is the large volume of regulatory documents and approvals needed for clinical research. The filing system can be in the form of a single project file or a number of files/filing cabinets, depending on what is deemed most appropriate for a particular clinical trial given its size and complexity. The regulatory documents and approvals within the TMF will be maintained alongside case report forms and source documentation.

2.0 BACKGROUND AND PURPOSE

The purpose of this SOP is to describe monitoring procedures for clinical trials monitored by the KHP-CTO in order that Clinical Trials conducted within the partner institutions comply with the UK and European Law. These laws comprise: Statutory Instrument 2004/1031 – the Medicines for Human Use (Clinical Trials) Regulations 2004 which transposed the European Union Directive 2001/20/EC for Clinical Trials into UK law effective from the 1st May 2004. The original UK regulations were amended in August 2006 to incorporate the EU Good Clinical Practice Directive (2005/28/EC) as Statutory Instrument 2006/1928 and as amended at any time.

Monitoring is defined as the act of overseeing the progress of a clinical trial, and of ensuring that it is conducted, recorded, and reported in accordance with the trial protocol, SOPs, GCP, and the applicable regulatory requirement(s).

The purpose of monitoring is to verify that:

- The rights and well-being of the human subjects are protected
- The reported trial data are accurate, complete and verifiable from source documents
- The conduct of the trial is in compliance with the currently approved protocol/amendment(s), GCP and the applicable regulatory requirements.
- Monitoring has an integral role in the Quality Control of a clinical trial and is designed to verify the ongoing quality of the trial.

3.0 SCOPE

All clinical trials sponsored by one or more of King's Health Partner Institutions, or clinical trials where the sponsor responsibilities are managed by the KHP-CTO, will be monitored as described in this SOP. Trials sponsored by organisations other than the Partner Organisations may also be monitored according to this SOP from time to time.

Trials co-sponsored by a Partner Organisation and an External Organisation will be monitored according to this SOP if the Sponsor Responsibility for GCP compliance has been delegated to the King's Health Partner Organisation.

Monitoring will be conducted by the KHP-CTO CRA Team and overseen by the Quality Manager or delegate. From time to time as required, monitoring may be contracted out to external organisations/CRAs, but oversight will be retained by the KHP-CTO

4.0 PROCEDURE

For clinical trials sponsored or managed by the Partner Organisations, , or clinical trials where the sponsor responsibilities are managed by the KHP-CTO, the CRA Team from the KHP-CTO is the main line of communication between the KHP-CTO (on behalf of the Sponsor) and the Investigator. The KHP-CTO ensures that the Investigator conducts the clinical trial in compliance with the final protocol and subsequent protocol amendments if any, as well as GCP and applicable safety reporting and regulatory requirements.

4.1 Selection of Clinical Research Associates

CRAs will be appointed by the KHP-CTO as detailed in the Quality Policy and be appropriately trained. In exceptional circumstances a delegate monitor may be appointed, however a contact within the KHP-CTO will also be given to Investigators. The CRA/delegate will have the sufficient scientific and/or clinical knowledge needed to monitor the trial adequately. Training records, including relevant qualifications, will be kept by the CRA/delegate. Oversight will be maintained by the KHP-CTO on behalf of the Sponsor.

CRAs/delegate will be expected to acquire an appropriate level of knowledge of assigned trial IMP(s), the protocol, information sheet and consent form, as well as the KHP-CTO SOPs, GCP and other applicable regulatory requirements.

4.2 Extent of Monitoring

Monitoring will be proportional to the objective, purpose, design, size, complexity, blinding, endpoints and risks of the clinical trial. It will be the KHP-CTO Quality Team's and Sponsor's R&D Dept responsibility to determine the appropriate level and nature of monitoring required for their clinical trial by risk assessment. Further to the risk assessment, detailed monitoring

requirements will be documented in the trial specific monitoring plan, activities will vary from trial to trial however will include the following:

4.3 CRA Responsibilities

1. The CRA/delegate will act as the main line of communication between the KHP-CTO (on behalf of the Sponsor(s)) and the Investigator.
2. The CRA/delegate will ensure that the Investigator provides all the required reports, notifications, applications, and submissions and that these documents are accurate, complete, timely, and legible, version controlled, dated and identify the trial.
3. The CRA/delegate will ensure that all documents and trial supplies needed to conduct the trial properly, and to comply with the applicable regulatory requirements, are available.
4. The CRA/delegate will ensure that the Investigator holds a copy of the current Reference Safety Information (RSI). The current RSI is defined by the version included in the initial CTA submission or as detailed in the Development Safety Update Report.
5. The ISF/TMF will be verified to ensure that all required documents are available for review, including all essential documents.
6. The CRA/delegate will verify that the source data location list reflects current practice.
7. The CRA/delegate will verify that the Investigator has adequate qualifications, resources and facilities, including laboratories, equipment and appropriately trained staff, to safely and properly conduct the trial and that these remain adequate throughout the trial period.
8. The CRA/delegate will verify that trial functions are performed as designated and not delegated to unauthorised individuals.
9. The CRA/delegate will verify that informed consent was obtained and documented prior to subject participation in the trial and that only eligible subjects are enrolled as detailed in the trial risk assessment and monitoring plans.
10. The CRA/delegate will verify that the Investigator follows the approved protocol and any approved amendment(s), GCP, relevant regulatory requirements and is adequately informed about the conduct of the trial. Deviations will be communicated to the Investigator and appropriate action designed to prevent recurrence of the detected deviations taken.
11. The CRA/delegate will verify that source documents and other trial records are accurate, complete and up-to-date, and check the accuracy and completeness of the CRF entries. The CRA will examine a proportion of CRFs as specified in the risk assessments and monitoring plan. Trials that were ongoing prior to the KHP-CTO Quality Team set up may not have a monitoring plan in place however, a minimum data set to include (but not

limited to) consent, eligibility criteria, TMF and ISF, Pharmacovigilance and IMP management will be monitored.

12. Where required by the monitoring plan with respect to the CRFs the CRA/delegate will verify that :
    a. The data required by the protocol are reported accurately in the CRFs and are consistent with the source documents.
    b. Any dose and/or therapy modifications are well documented for each of the trial subjects.
    c. Adverse events, concomitant medications and concurrent illnesses are reported in accordance with the protocol in the CRFs.
    d. Visits that the subjects fail to make, tests that are not conducted, and examinations that are not performed are clearly reported as such in the CRFs.
    e. All withdrawals and dropouts of enrolled subjects from the trial are reported and explained on the CRFs.

13. The CRA/delegate will inform the Investigator of any CRF entry error, omission or illegibility and ensure that appropriate corrections, additions or deletions are made, dated, explained (if necessary) and initialled by the Investigator or an authorised individual. The monitor is not permitted to make such changes.

14. Verification and collection of subject data should be performed according to data protection laws and requirements.

15. The CRA/delegate will determine whether all SAEs are appropriately recorded in the source documents and have been reported within the time periods required by GCP, the protocol, the REC, the KHP-CTO and the applicable regulatory requirement(s).

16. Where required with respect to the IMP the CRA/delegate will ensure that:
    a. Storage times and conditions are acceptable and that supplies are sufficient.
    b. IMP is supplied only to subjects who are eligible, at the protocol specified dose(s) and according to randomised treatment allocation, if applicable.
    c. Subjects are provided with necessary instruction on properly using, handling, storing and returning IMP(s).
    d. The receipt, use and return of any IMP(s) at the trial sites are controlled and documented adequately.
    e. Disposal of unused IMP(s) complies with applicable regulatory requirement(s) and is in accordance with the sponsors SOP.

17. The CRA/delegate will verify that blinding has been maintained and ensure any code breaks are properly handled and documented according to the protocol and/or relevant code break SOP.

The CRA Team will immediately notify the Quality Manager or KHP-CTO Director in the event of any suspicion of scientific misconduct, fraud or breach of GCP. This will then be dealt with according to appropriate local organisational policy and the KHP-CTO Breach of GCP SOP.

4.4 Monitoring Report

Following a monitoring visit, the CRA will promptly submit a written report to the KHP-CTO (acting on behalf of the Sponsor). This will be done using the Monitoring Visit Report Form (see Section 5.1). Any other communication with the trial site which requires documentation will be recorded on a Contact Comment Form or email (See Section 5.2).

A Note to File (see Section 5.3) will be used to document any issues and these should be filed in the ISF and/or TMF with copies submitted to the KHP-CTO. Deviations from the protocol will be recorded on a deviation tracker.

The report or form will be reviewed promptly after the visit or communication and signed by an authorised individual within the KHP-CTO.

The Investigator will be informed in writing (NB: email is an acceptable form of communication) of any problem(s) that were identified during the monitoring visit and the required action points. If there is evidence of systematic failure to comply with GCP retraining will be given and trial management/Sponsor informed.

After each monitoring visit is completed and report written the CRA will update MATTS with the current subject recruitment status.

5.0 RELATED TEMPLATES

5.1 Monitoring Visit Report Form template

5.2 Contact Comment Form template

5.3 Note to File template

5.4 Monitoring Plan

6.0 APPROVAL AND SIGNATURE

[signature]

Jackie Pullen
Director, King's Health Partners Clinical Trials Office

1st October 2018
Date
