# Supplementary material for: Vitrectomy, subretinal Tissue plasminogen activator and Intravitreal Gas for submacular haemorrhage secondary to Exudative Age-Related macular degeneration (TIGER): study protocol for a phase 3, pan-European, two-group, non-commercial, active-control, observer-masked, superiority, randomised controlled surgical trial
Source: Trials. 2022 Jan 31;23:99. doi: 10.1186/s13063-021-05966-3 (PMC8805308; doi:10.1186/s13063-021-05966-3)
Supplement: Supplementary file 5 — Additional file 5. Appendix 5: Trial Steering Committee Charter. [file 13063_2021_5966_MOESM5_ESM.pdf]

9 Reporting

Prior to a TSC meeting a report will be prepared by the Trial Statistician with input from Trial Manager as required, and circulated to TSC members at least a week before the meeting.

On consideration of the information presented at these meetings, the TSC should provide recommendations of appropriate action in writing to the TMG who will be responsible for implementing any actions. The TSC may also provide feedback to the DMEC and where appropriate to the Sponsor/Funder.

Minutes of the meeting including key points and actions will be prepared by the Chair or Trial Manager. These minutes will describe the proceedings and include the recommendations of the TSC. All members of the TSC must agree the minutes and these will be signed off by the TSC Chair on behalf of all members. Minutes will be circulated to all TSC members, the TMG, the Sponsor and, the Trial Funder (Fight for Sight). Approved Minutes will be filed in the Trial Master File.

Decisions and recommendations by the TSC should be unanimous if not a vote may be taken. The role of the Chair is to summarise discussions and encourage consensus. Therefore it is best for the chair to give his/her own opinion last. It is important that the implications (ethical, statistical, practical and financial) for the trial be considered before any decision is made.

10 Contents of the TSC Reports

An outline of the contents of the TSC report is given below, but can be adapted based on the requirement of the TSC:

- Outline of the study
- Statistical consideration and design
- Major protocol amendments
- Patient screening
- Study accrual by month/total
- Baseline characteristics
- Safety reporting
- Follow up data available
- Any matters affecting the trial
- Compliance by patients to clinic visit

11 Conflicts of interest

TSC members should not have any apparent financial, scientific or intellectual conflict of interest that could prevent them from objectively reviewing the study protocol, interim and final data and giving advice to the TMG. TSC members should disclose to the Chair any other conflicts they consider relevant and provide the conflicts of interest form given in Annex 1. Any members who develop

6 Role of the TSC Chair

- Arrange the first meeting of the TSC with the assistance of the CI to agree contents of charter and set up schedule of meetings
- Establish clear reporting lines
- Become familiar with the role of the DMEC
- Provide an independent, experienced opinion if conflicts arise between the needs of the research team, the Funder, the Sponsor and/or any other agencies
- Leading the TSC to provide regular, impartial oversight of the trial, especially to identify and pre-empt problems
- Ensuring that changes to the protocol are debated and endorsed by other members of the TSC

7 TSC meetings

- The responsibility for calling and organising a TSC meeting lies with the CI/Trial Manager in association with the TSC Chair.
- Meetings may be in person or virtual
- All TSC members will be provided with study documents (e.g. protocol, proposed statistical analysis plan (SAP), Patient Information Sheets, etc) and the previous TSC report prior to the meeting.
- The first TSC meeting should discuss, revise and finalise the terms of reference, agree the content of the TSC charter and sign any declaration, and agree the frequency of the meetings.
- Meeting frequency will typically be 6 to 12-monthly and follow DMEC meetings.
- Meetings can also be held at any time at the request of the CI, Sponsor or TSC chair
- The final TSC meeting will be arranged when target recruitment is completed, all data collected and cleaned and the database is locked. This final meeting will be held to discuss final/completed data and interpretation, and publication timeless. If the study is terminated prematurely, no final study meeting is required.

8 Attendance and Quoracy

Every effort will be made to ensure that all TSC members can attend the meetings. The Trial Manager or delegate should try and find a date that enables this. The CI must try to attend all meetings, especially if major actions are expected.

For decisions to be made the meeting must be quorate, to include at least 2 independent members of the TSC (including the chair), the CI, independent statistician and a representative from the TMG. Non-quorate meetings can occur, but a quorum is required for any meeting that requires a vote or major action.

If the TSC is considering major actions the TSC Chair should communicate with absent members, including the CI if not present at the meeting, as soon after the meeting as possible to determine whether they all agree. If there is disagreement amongst absent members a further meeting should be arranged with the full TSC.

4 Agreements

TSC members should formally register their agreement to be a member of the committee as well as their agreement with the contents of the charter, trial confidentiality and should declare any potential conflicts of interest.

Independent members should complete and return a signed agreement and competing interests form provided at the end of this charter.

5 Responsibilities

The TSC on behalf of the Sponsor and Funder will have overall responsibility for the design and conduct of the trial and for safeguarding the rights, safety and well being of participants. Responsibilities of the TSC to include:

- Reviewing selection/recruitment/retention of participants and their management
- Finalising and reviewing study protocol and other study documentation.
- Determine if amendments to the protocol or changes to study conduct are required and deciding on changes to these and to study conduct in general. Any changes to trial documentation or conduct must be notified to the TSC.
- Reviewing adherence to the protocol by Investigators and participants
- Assessing the impact and relevance of external evidence
- Assessing integrity and completeness of data collected
- Monitoring the overall conduct of the trial, ensuring that it follows the standards set out in the guidelines of GCP, assessing the safety and efficacy of the interventions, recruitment figures and completion of trial assessments.
- Reviewing, commenting and making decisions on extension requests.
- Reviewing the recommendations of the DMEC (if applicable) and/or other study committees and suggesting appropriate action to the TMG
- Monitoring the progress of study/trial and deciding on appropriate action in order to maximise the chances of completing it within the agreed timelines.
- Considering new information relevant to the study e.g. results from other studies that may have a bearing to the conduct of the study and deciding on appropriate action.

The TSC may recommend early termination of the trial or modification of the study design in the event of a clear outcome derived from accumulating data or on the basis of information available from other sources or on safety grounds.

The TSC should be available to provide independent advice as required not just when meetings are scheduled.

In agreeing to join the TSC, members agree to maintain the confidentiality of all information received and not to discuss confidential issues from their involvement in the study, until the primary results have been published.

Prof. Noemi Lois	Non-voting Trial Clinician
Prof. David Steel	Non-voting Trial Clinician
Ms Cathy Yelf	PPI representative
Steven Smith	Fight for Sight<br>Representative of the Funder
Ingrid Brumarescu	CRA - KHPCTO<br>Representative of the Sponsor

The responsibility for calling and organising TSC meetings lies with the Chief Investigator (CI) in association with the Chair. The Chair assisted by the CI is responsible for facilitating the meetings and summarise discussions. The Chair will approve the appointment of the members of the TSC at the first meeting.

The TSC membership is for the duration of the trial. If any members leave the TSC, the TMG should provide replacements promptly for appointment by the Chair.

Interaction between TSC and other study committees

At each DMEC meeting, the DMEC will recommend whether the study should continue, stop, be suspended, or be modified, based on their findings.

The recommendations of the DMEC to the TSC may include:

- Discontinuation of the study.
- Permanently or temporarily halt enrollment into the study.
- Modification of the study protocol.
- Continue the study according to the protocol and any related amendments.

Upon receipt of the DMEC recommendations, the TSC will consider the DMEC recommendations, review the status of the trials, and determine a course of action. The TSC may accept, reject, or modify DMEC recommendations. If the DMEC recommends discontinuation of the study or halting enrolment, the TSC Chair will convene an urgent meeting of the TSC.

The TSC may identify expert individuals to review the DMEC reports. These individuals will not have the clinical, statistical, regulatory or other expertise needed to assist the TSC. The Sponsor, Chief Investigator, TSC and DMEC will assure that confidentiality of the data, and DMEC recommendations, are maintained.

- To ensure that the rights, safety and wellbeing of the trial participants are the most important considerations and should prevail over the interests of science and society.
- To consider the recommendations of Research Ethics committees, the trial/study Data Monitoring and Ethics Committee (DMEC).
- The TSC should inform the TMG if:
  - There are concerns about the safety of participants
  - Accrual is too low to provide meaningful results
  - It is evident that if the study continues it would fail to provide a clear benefit
- To recommend whether to continue or terminate the study or further adapt it based on safety and efficacy considerations.

3 Membership and Primary responsibilities of the TSC

The TIGER TSC is a multidisciplinary group comprising of the following members who jointly have responsibility for the design, conduct and evaluation of the clinical research project.

**Name**	**Role**
Dr Richard Wormald	An independent Chair
Prof Tim Jackson	Chief Investigator
Riti Desai	Trial Manager (Member of TMG)
Ana Gonzales	Ass.Trial Manager (Member of TMG)
Chan Ning Lee	Research fellow (Member of TMG)
Yanzong Wang	Study statistician
Hatem Wafa	Study statistician
Prof. Ramin Tadayoni	Independent clinician
Prof. Dr. Jan van Meurs	PI representative
Dr Gabriella Czanner	Independent statistician
Prof. Dr. Jost Hillenkamp	Clinician (Non-voting country lead)
Prof. Catherine Creuzot-Garcher	Clinician (Non-voting country lead)
Prof. Barney Reeves	Non-voting Trial Methodologist

TIGER
Trial Steering Committee Charter

Full Title: Vitrectomy, subretinal Tissue plasminogen activator and Intravitreal Gas for submacular haemorrhage secondary to Exudative age-Related macular degeneration (TIGER): a phase 3, pan-European, two-group, active-control, observer-masked, superiority, randomised controlled surgical trial.

Protocol short title: Vitrectomy and subretinal TPA for submacular haemorrhage secondary to wet AMD (TIGER).

IRAS ID: 276366

REC No: 20/EE/0293

EudraCT Number (if applicable): 2020-004917-10

ISRCTN Number: NA

ClinicalTrials.gov Identifier (if applicable): NCT04663750

Investigational Product: Tissue Plasminogen Activator (TPA, Alteplase, Actilyse)

Chief Investigator: Prof Tim Jackson

Sponsor: King's College London (lead sponsor) and King's College Hospital NHS Foundation Trust (clinical co-sponsor)

1 Introduction

The role of the Trial Steering Committee (TSC) is to provide overall supervision for TIGER trial on behalf of the Trial Sponsor and to ensure that the trial is conducted according to the guidelines for Good Clinical Practice (GCP), Research Governance Framework for Health and Social Care and all relevant regulations and local policies.

The background to this trial, its objectives, assessments, interventions, etc, are described in the trial protocol.

The purpose of this document is to define the roles and responsibilities of the trial TSC and to guide its activities, its relationship with other trial committees, its membership, and the format, purpose and timings of its meetings. The charter also describes the procedures for ensuring confidentiality and proper communication to and from the TSC and an outline of the content of the reports to be provided to the TSC.

2 Terms of reference

- To provide advice, through its Chair, to the Trial Management Group (TMG), the Sponsor and on all aspects of the trial.
- To monitor and supervise the progress of the trial towards its overall objectives, review accrual and results of the trial, adherence to the protocol, patient safety and the consideration of new information of relevance to the trial and the research question.

significant conflicts of interest during the course of the trial should resign from the TSC.

12 Publication

Manuscripts that arise from the trial will be shared with the TSC and members will be able to comment. The TSC members and their affiliations will be acknowledged in reports of the trial.

13 Protocol Amendments

The Sponsor retains the right to make amendments as it sees fit, but protocol amendments, other than administrative amendments, will be presented to the TSC for its approval at the next scheduled TSC meeting. If a proposed amendment affects the safety of trial participants, or the overall integrity of the trial, then the CI will inform and ideally seek approval of the TSC Chair before implementing change. The Chair may request a special meeting of the TSC to review proposed amendment.

14. Sponsor Acknowledgement

Signed on Behalf of King's College London and King's College Hospital NHS Foundation Trust.

Signature: Jasmine Palmer

Name & Job Title: JASMINE PALMER, RESEARCH GOVERNANCE SPECIALIST

Date: 23/08/2021

TIGER
Trial Steering Committee Charter

Annexe 1
Agreement and competing interests form for independent members of the TIGER Trial Steering Committee/Study Steering Committee.

Please complete the following document and return to:
Riti Desai
Ophthalmology Research Manager
TIGERSTUDY Email: kch-tr.tigerstudy@nhs.net

☐ I have read and understood the TSC charter <version number> dated <date>

☐ I agree to join the TSC for this study/trial as an independent member

☐ I agree to treat all sensitive trial data and discussions confidential

The avoidance of any perception that members of the TSC may be biased in some fashion is important for the credibility of the decisions made by the TSC and for the integrity of the trial.
Possible competing interest should be disclosed via the trials office. In many cases simple disclosure up front should be sufficient. Otherwise, the (potential) TSC member should remove the conflict or stop participating in the TSC. Table 1 lists potential competing interests:

Table 1: Potential competing interests for independent members

1. Stock ownership in any commercial companies involved.<br>2. Stock transaction in any commercial company involved (if previously holding stock)<br>3. Consulting arrangements with the Sponsor<br>4. Frequent speaking engagements on behalf of the intervention<br>5. Career tied up in a product or technique assessed by the trial<br>6. Hand-on participation in the trial<br>7. Involvement in the running of the trial<br>8. Emotional involvement in the trial<br>9. Intellectual conflict e.g. strong prior belief in the trial's experimental arm<br>10. Involvement in regulatory issues relevant to the trial procedures<br>11. Investment (financial or intellectual) in competing products<br>12. Involvement in the publication

☐ **NO,** I have no competing interests to declare

☐ **YES,** I have competing interests to declare (please detail below)

____

____

____

Name: ______________________

Signed: ______________________ Date: ______________
