# Supplementary material for: Vitrectomy, subretinal Tissue plasminogen activator and Intravitreal Gas for submacular haemorrhage secondary to Exudative Age-Related macular degeneration (TIGER): study protocol for a phase 3, pan-European, two-group, non-commercial, active-control, observer-masked, superiority, randomised controlled surgical trial
Source: Trials. 2022 Jan 31;23:99. doi: 10.1186/s13063-021-05966-3 (PMC8805308; doi:10.1186/s13063-021-05966-3)
Supplement: Supplementary file 6 — Additional file 6. Appendix 6: Data Monitoring and Ethics Committee Charter. [file 13063_2021_5966_MOESM6_ESM.pdf]

CHARTER FOR TIGER STUDY DATA MONITORING AND ETHICS COMMITTEE

**CONTENT**	
**1. Introduction**	
	Full Title: Vitrectomy, subretinal Tissue plasminogen activator and Intravitreal Gas for submacular haemorrhage secondary to Exudative age-Related macular degeneration (TIGER): a phase 3, pan-European, two-group, active-control, observer-masked, superiority, randomised controlled surgical trial.<br><br>Short title: Vitrectomy and subretinal TPA for submacular haemorrhage secondary to wet AMD (TIGER).<br><br>REC No: 20/EE/0293<br><br>EudraCT Number: 2020-004917-10<br><br>ISRCTN Number: NA<br><br>ClinicalTrials.gov Identifier: NCT04663750<br><br>Investigational Product: Tissue Plasminogen Activator (TPA, Alteplase, Actilyse)<br><br>Chief Investigator: Prof Tim Jackson<br><br>Sponsor: King's College London (lead sponsor) and King's College Hospital NHS Foundation Trust (clinical co-sponsor)
Objectives	Objective of trial: To assess the safety and efficacy of vitrectomy, subretinal tissue plasminogen activator (TPA), and gas tamponade as a treatment for SMH secondary to exudative AMD, versus standard of care.<br><br>Intervention:<br><br>Pars plana vitrectomy, subretinal injection of recombinant TPA (Alteplase, Actilyse, Boehringer Ingelheim) up to a maximum of 25 micrograms in 0.2 mls, intravitreal 20% sulfahexafluoride (SF_6) gas tamponade, and intravitreal 2 mg aflibercept (Eylea, Bayer).<br><br>After surgery participants will be advised to sit up and lean forward during the day for 5 days, and sleep with the operative cheek dependent for 10 days.<br><br>Intravitreal 2 mg aflibercept will be injected monthly for two further doses, then 2-monthly until month 12 (in both the treatment and control groups).
Trial Design and Flow Diagram<br><br>TIGER is a phase 3, multicentre, pan-European, non-commercial, randomised, two-group, active control, superiority, observer-masked, surgical trial. It is summarized in the following diagram:	

KCH Approved [signature] 23/03/2021

CONTENT

TIGER Flow Diagram

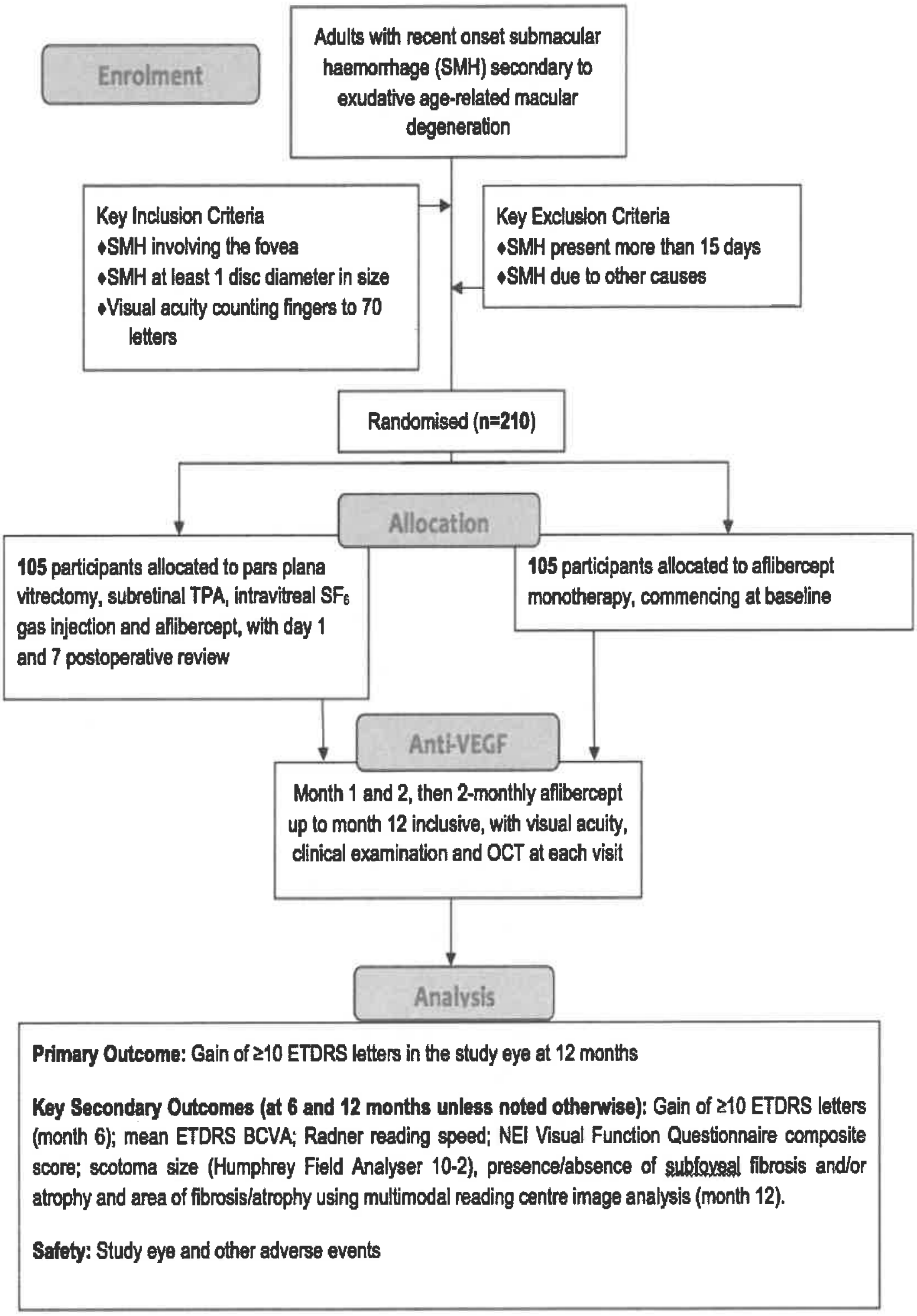

CONTENT	
	The purpose of this document is to describe the roles and responsibilities of the independent Data Monitoring and Ethics Committee (DMEC) for the TIGER trial, including the timing of meetings, methods of providing information to and from the DMEC, frequency and format of meetings, statistical issues and relationships with the Trial Steering Committee (TSC).
**2. Roles and responsibilities**	To protect and serve trial patients (especially regarding their safety) and to assist and advise the Chief Investigator (CI) so as to protect the validity and credibility of the trial.<br>All members of the DMEC agree to carefully read and consider the protocol in its entirety.<br>A copy of each DMEC member's curriculum vitae will be collected at the outset of their involvement, and retained by the Sponsor. This may be provided to any regulatory agency that requires a copy.<br>The DMEC should receive and review the progress and accruing data of this trial and advise on the conduct of the trial to the TSC.

**Roles of DMEC**	Ongoing and as required review of the trial's progress including data quality, and main outcomes and safety data.<br>• assess data quality, including completeness (and by so doing encourage collection of high-quality data)<br>• monitor losses to follow-up<br>• monitoring evidence for treatment differences in the main efficacy outcome measures<br>• monitor evidence for treatment harm (eg toxicity data, SAEs, deaths)<br>• decide whether to recommend that the trial continues to recruit participants or whether recruitment should be terminated either for everyone or for some treatment groups and/or some participant subgroups<br>• suggest additional data analyses<br>• advise on protocol modifications suggested by investigators or sponsors (eg to inclusion criteria, trial endpoints, or sample size)<br>• monitor planned sample size assumptions<br>• monitor continuing appropriateness of patient information<br>• monitor compliance with previous DMEC recommendations<br>• considering the ethical implications of its recommendations<br>• assess the impact and relevance of external evidence
**3. Before or early in the trial**	
	All potential DMEC members should have sight of the protocol/outline before agreeing to join the committee. Before recruitment begins the trial will have undergone review by the funder and sponsor, scrutiny by other trial committees and a research ethics committee. Therefore, if a potential DMEC member has major reservations about the trial (eg the protocol or the logistics) they should report these to the trial office and may decide not to accept the invitation to join. DMEC members should be independent and constructively critical of the ongoing trial, but also supportive of the aims and methods of the trial.

	The initial meeting of the DMEC will be organizational in nature. The meeting will further acquaint the DMEC members with the TIGER trial protocol and other pertinent information. The meeting will allow DMEC members to provide input on future interactions between the DMEC, the Sponsor, and the TSC. Invited attendees will include the DMEC members, the Chief Investigator, Trial Statistician, and usually also the Trial Manager. The format of data reports will be agreed between the DMEC and the Trial Statistician.<br><br>The Trial Statistician will be appointed by the Sponsor. The Trial Statistician will prepare reports for the DMEC meetings, at least seven days prior to each meeting. The Trial Statistician will not have access to the closed reports prepared by the DMEC.<br><br>Consideration should be given to an initial "dummy" report, including the use of shell (empty) tables, to familiarise the DMEC members with the format that will be used in the reports.<br><br>The DMEC should be aware of any regulatory implications of their recommendations.<br><br>Meeting frequency will be determined by the DMEC but will usually be 6 to 12-monthly. The CI, Sponsor, TSC and the DMEC may each request an unscheduled DMEC meeting.<br><br>In taking on a role on the DMEC, members confirm (1) that they agree to be on the DMEC and (2) that they agree with the contents of this Charter.
**4. Composition**	Membership will consist of approximately three members and will include at least one clinician experienced in the clinical area and at least one statistician.<br><br>The members should be independent of the trial (eg should not be involved with the trial in any other way or have some competing interest that could impact on the trial).<br><br>The members of the DMEC for this trial are:<br>(1) Prof Craig Ramsay -Statistician (Chair)<br>(2) Prof Heinrich Heimann- Consultant Ophthalmologist<br>(3) Prof Susanne Binder -Consultant Ophthalmologist<br><br>The Chair should have previous experience of serving on DMECs and experience of chairing meetings, and should be able to facilitate and summarise discussions. The Chair is expected to facilitate and summarise discussions.<br><br>The DMEC membership will serve for the duration of the TIGER trial. If a member cannot continue to serve on the DMEC, the reason must be indicated in writing to the DMEC Chair. If a member, including the Chair, leaves the DMEC, a replacement will be sought. If the TSC has concerns that a member of the DMEC is not fulfilling his or her role, they may, by majority vote, require that the member resigns.

	The trial statistician will produce (or oversee the production of) the report to the DMEC and will participate in DMEC meetings, guiding the DMEC through the report, participating in DMEC discussions and, on some occasions, taking notes.<br><br>The Chief Investigator (CI) and Trial Manager will only attend open sessions of the DMEC meeting. The other Trial Management Group (TMG) members will not usually be expected to attend but can attend open sessions when necessary (See Organisation of DMEC Meetings).
**5. RELATIONSHIPS**	The DMEC does not make decisions about the trial, but rather makes recommendations to the Sponsor and Trial Steering committee via its Chair.<br><br>Members should be reimbursed for reasonable travel and accommodation expenses. No other payment is planned.<br><br>DMEC members should not use interim results to inform trading in pharmaceutical shares, and careful consideration should be given to trading in stock of companies with competing products.<br><br>Competing interests should be disclosed. These are not restricted to financial matters – involvement in other trials or intellectual investment could be relevant. Although members may well be able to act objectively despite such connections, complete disclosure enhances credibility. (See Annex 1)<br><br>Members of the DMEC will not buy, sell, or hold stock or stock options Boehringer Ingelheim, or competing companies, until the trial is concluded and the final outcomes have been reported in the scientific literature. Each member agrees not to serve as a paid consultant to Boehringer Ingelheim, or a competing company, for the duration of the study. This guideline also applies to the member's spouse and dependents. Members of the DMEC will be responsible for advising the DMEC Chair, the TSC, and Sponsor of any changes in relation to their financial interests, or any other matter that creates a potential conflict of interest. The Sponsor will collect and retain the declarations of interest and if any potential conflict of interest arises, the Sponsor will inform the TSC. The TSC will be responsible for deciding whether a financial or other interest impacts on a member's objectivity, and may require a member to resign from the DMEC.
**6. ORGANISATION OF DMEC MEETINGS**	The exact frequency of meetings will depend upon any statistical plans specified, and otherwise on trial events. The wishes of the DMEC and needs of the trial team will be considered when planning each meeting. It is recommended that the DMEC meet at least yearly.<br><br>Meetings may be face-to-face or virtual.

	The meeting will comprise open and closed sessions. Closed and open sessions should be defined. The DMEC members will determine who to admit to the closed sessions. In open sessions, all those attending the closed session are joined by the CI and Trial Manager, and sometimes also representatives of the sponsor, funder, or regulator, as relevant.
**7. Trial documentation and procedures to ensure confidentiality and proper communication**	All members will treat as confidential the reports, meetings (virtual or face-to-face), discussions, emails and minutes pertaining to the TIGER trial.<br><br>Open sessions: Accumulating information relating to recruitment and data quality (eg data return rates, treatment compliance) will be presented. Adverse event details based on pooled data will be presented and total numbers of events for the primary outcome measure and other outcome measures may be presented, at the discretion of the DMEC.<br><br>Closed sessions: In addition to all the material available in the open session, the closed session material will include efficacy and safety data by treatment group, if the DMEC determine that unmasked data review is required.<br><br>DMEC members do **not** have the right to share confidential information with anyone outside the DMEC, including the CI.

	The DMEC will report its recommendations in writing to the TSC. This should be copied to the Trial Manager and if possible should be sent in time for consideration at the next TSC meeting. If the trial is to continue largely unchanged a template letter to the TSC is available, if desired, in Annex 2.<br><br>Where possible, the trial statistician will provide the DMEC with his/her report at least 1 week before any meetings.<br><br>The DMEC members should maintain minutes of their meetings and provide these to the TMG for filing only after the trial has reported.
**8. Decision making**	At each meeting, the DMEC will recommend whether the study should continue, stop, or be modified based on their findings. The DMEC will provide written recommendations about the trial to the TSC Chair and CI within 14 days of the meeting. A shorter timeline may be required if there are urgent findings.<br><br>Upon receipt of the DMEC recommendations, the TSC will consider the DMEC recommendations, review the status of the trials, and determine a timely course of action. The TSC may identify expert individuals to review the DMEC Reports. These individuals will have the clinical, statistical, regulatory or other expertise needed to assist the TSC. The TSC may seek input from regulatory agencies and then make a decision to accept or disregard the recommendation of the DMEC. The Sponsor, Chief Investigator, TSC and DMEC will assure that confidentiality of the data, and DMEC recommendations, are maintained.<br><br>If the DMEC recommends stopping the trial and the TSC agrees by majority vote, then the Sponsor will inform all regulatory agencies of the decision and notify all investigational centers.<br><br>Other possible DMEC recommendations could include:-<br>• Stopping recruitment within a subgroup<br>• Extending recruitment (based on actual control arm response rates being different to predicted rather than on emerging differences) or extending follow-up<br>• Sanctioning and/or proposing protocol changes<br><br>An interim analysis is not planned. Nonetheless, the DMEC will be at liberty to request an interim analysis and, if agreed by the Sponsor and TSC, they will work with the Trial Statistician to determine the nature of the analysis.<br><br>The Sponsor will be responsible for informing the DMEC of all substantial amendments to the protocol. The DMEC are not required to approve amendments, but the DMEC may submit recommendations to the Sponsor and TSC.

	• The process of decision making, including whether there will be voting or other formal methods of achieving consensus will be determined by the DMEC at their initial meeting. The method of deliberation should not be revealed to the overseeing committee as this may reveal information about the status of the trial's data.<br>• The Chair will summarise discussions and encourage consensus; it may be best for the Chair to give their own opinion last.<br>It is recommended that every effort should be made for the DMEC to reach a unanimous decision. If the DMEC cannot achieve this, a vote may be taken, although details of the vote should not be routinely included in the report to the TSC as these may inappropriately convey information about the state of the trial data.<br>It is important that the implications (eg ethical, statistical, practical, financial) for the trial be considered before any recommendation is made.
	A quorum of three DMEC members, including the Chair, is required to hold any meeting that requires voting, such as a recommendation to halt, suspend, and substantially alter the trial. A majority vote of members in attendance at the meeting passes a recommendation to the TSC or Sponsor. Non-quorate meetings may proceed if required, and minutes should be made as per quorate meetings, but substantial recommendations may not be voted on.<br><br>If a member does not attend a meeting, it should be ensured that the member is available for the next meeting. If a member does not attend a second meeting, they should be asked if they wish to remain part of the DMEC. If a member does not attend a third meeting, they should usually be replaced.

**9. Reporting**	
	The DMEC's decision will be communicated in writing to the Trial Steering Committee and copied to Trial Manager and CI. This should usually be within 3 weeks.<br>The DMEC Chair should sign off any minutes or notes.<br>If the DMEC has serious problems or concerns with the TSC decision a meeting of these groups should be held. The information to be shown would depend upon the action proposed and the DMEC's concerns. Depending on the reason for the disagreement confidential data will often have to be revealed to all those attending such a meeting. The meeting should be chaired by a senior member of the trials office staff or an external expert who is not directly involved with the trial.
**10. After the trial**	
	The DMEC will be invited to review the main trial publication, to comment on the data interpretation should they wish.<br><br>DMEC members should be named and their affiliations listed in the main report, unless they explicitly request otherwise.

Annex 1: Suggested competing interests form

Potential competing interests of Data Monitoring Committee members for *[TIGER trial (and sponsor's ID)]*

The avoidance of any perception that members of a DMEC may be biased in some fashion is important for the credibility of the decisions made by the DMEC and for the integrity of the trial.

Possible competing interest should be disclosed via the Trial Manager. In many cases simple disclosure up front should be sufficient. Otherwise, the (potential) DMEC member should remove the conflict or stop participating in the DMEC. Table 1 lists potential competing interests.

Table 1: Potential competing interests

- Stock ownership in any commercial companies involved
- Stock transaction in any commercial company involved (if previously holding stock)
- Consulting arrangements with the sponsor
- Frequent speaking engagements on behalf of the intervention
- Career tied up in a product or technique assessed by trial
- Hands-on participation in the trial
- Involvement in the running of the trial
- Emotional involvement in the trial
- Intellectual conflict eg strong prior belief in the trial's experimental arm
- Involvement in regulatory issues relevant to the trial procedures
- Investment (financial or intellectual) in competing products
- Involvement in the publication

Please complete the following section and return to the trials office.

☐ **No,** I have no competing interests to declare
☐ **Yes,** I have competing interests to declare (please detail below)

Please provide details of any competing interests: ____________________

____

____

Name: ____________________

Signed: ____________________ Date: ____________________

Annex 2: Suggested report from DMEC to TSC where no recommendations are being made

[Insert date]

To: Chair of Trial Steering Committee

Dear *[Chair of Trial Steering Committee]*

The Data Monitoring Committee (DMEC) for the *[insert trial name]* trial met on *[meeting date]* to review its progress and interim accumulating data. *[List members] attended* the meeting and reviewed the report.

We congratulate the trial organisers and collaborators on the progress and conduct of the trial and the presentation of the data. The trial question remains important and, on the basis of the data reviewed at this stage, we recommend continuation of the trial according to the current version of the protocol *[specify protocol version number and date]* with no changes.

We shall next review the progress and data *[provide approximate timing]*

Yours sincerely,

[Name of meeting Chair]
Chair of Data Monitoring Committee

On behalf of the DMEC (all members listed below)

DMEC members:
(1) [*Insert name and role*]
(2) [*Insert name and role*]
(3) [*Insert name and role*]
