# Supplementary material for: Vitrectomy, subretinal Tissue plasminogen activator and Intravitreal Gas for submacular haemorrhage secondary to Exudative Age-Related macular degeneration (TIGER): study protocol for a phase 3, pan-European, two-group, non-commercial, active-control, observer-masked, superiority, randomised controlled surgical trial
Source: Trials. 2022 Jan 31;23:99. doi: 10.1186/s13063-021-05966-3 (PMC8805308; doi:10.1186/s13063-021-05966-3)
Supplement: Supplementary file 7 — Additional file 7. Appendix 7: Patient Informed Consent Form. [file 13063_2021_5966_MOESM7_ESM.docx]

**CONSENT FORM**

**TIGER: Vitrectomy, subretinal Tissue plasminogen activator and Intravitreal Gas for submacular haemorrhage secondary to Exudative age-Related macular degeneration**

Lead investigator: **INSERT DETAILS**

**Please initial box**

1. **I confirm that I have read and understood the information sheet dated 15 February 2021, Version 1.2 for the above study and have had the opportunity to ask questions.**
2. **I understand that my participation is voluntary and that I am free to withdraw at any time, without giving any reason. Leaving the study will not stop me accessing whatever standard clinical care is available, or my legal rights.**
3. **I understand that my medical notes may be looked at by responsible individuals from the study organisers, other researchers involved in this study, and from regulatory authorities where it is relevant to my taking part in research. I give permission for these individuals to have access to my records.**
4. **I agree to my GP being informed of my participation in this study**
5. **I agree to take part in the above study.**

**When you have initialled all the boxes above, please sign below and add the date of signing yourself.**

_________________________ _______________ ______________

**Name of Patient** **Date** **Signature**

_________________________ _______________ ______________

**Name of Researcher Date Signature**

**taking consent**

**1 copy for patient; 1 for researcher; 1 to be kept with hospital notes.**
