# Supplementary material for: Vitrectomy, subretinal Tissue plasminogen activator and Intravitreal Gas for submacular haemorrhage secondary to Exudative Age-Related macular degeneration (TIGER): study protocol for a phase 3, pan-European, two-group, non-commercial, active-control, observer-masked, superiority, randomised controlled surgical trial
Source: Trials. 2022 Jan 31;23:99. doi: 10.1186/s13063-021-05966-3 (PMC8805308; doi:10.1186/s13063-021-05966-3)
Supplement: Supplementary file 8 — Additional file 8. Appendix 8: Patient Information Sheet. [file 13063_2021_5966_MOESM8_ESM.docx]

**TIGER study**

**Full Title: Vitrectomy, subretinal Tissue plasminogen activator and Intravitreal Gas for submacular haemorrhage secondary to Exudative age-Related macular degeneration (TIGER): a phase 3, pan-European, two-group, active-control, observer-masked, superiority, randomised controlled surgical trial.**

**IRAS ID: 276366**

**PATIENT INFORMATION SHEET**

***Principal Investigator:*** INSERT LOCAL PI

***Telephone number:*** INSERT LOCAL PI TELEPHONE

***Emergency Contact:*** INSERT CONTACT DETAILS

***Study website:*** TBC

Version number: Version 1.2 - 15 February 2021

**If you need the writing in this document to be larger, please tell the study doctor.**

**Purpose of this study**

The aim of this study is to find the best treatment for people with wet age-related macular degeneration (AMD) who have developed a submacular haemorrhage. You are invited to join the study because you have wet AMD and you have a submacular haemorrhage.

The study is called TIGER.

Before you decide to take part in this study, it is important that you understand what the study involves. Please take time to read the following information and, if you wish, discuss it with your doctor, friends and family.

**Summary**

- You are invited to join the TIGER study because you have blood in the back of your eye.
- The blood is in your macula, the area responsible for your central sight.
- The bleeding happened because you have age-related macular degeneration (AMD).
- Without treatment your sight is likely to be poor
- If you join TIGER you will get one of two treatments. The study aims to find out which is best. The two treatment are:

1. Eye injections to stop the bleeding

2. Eye injections to stop the bleeding plus eye surgery to clear the blood. The surgery puts a clot-busting drug and a gas bubble into your eye, to dissolve the clot and push the blood away from the macula

- Either of these treatments should be much better than doing nothing
- If you want to join the study let us know quickly, as the blood can damage sight within few days.
- We will then check if you are suitable (“eligible”) to join the study.
- If you successfully join the study you will have 7-9 clinic visits over 12 months so we can check how the treatment is working.

**What are the retina, the macula, wet AMD, and submacular haemorrhage?**

The retina is the layer that covers the inside of the eye. The macula is in the centre of the retina. The macula contains cells that give us our central vision that we use for reading and recognising faces etc.

Wet AMD is a disease where new, abnormal blood vessels grow through the macula and leak fluid. The macula should be dry to work well. If the macula gets wet then it does not work properly and the sight is affected. The treatment for


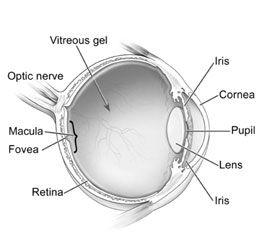


**Image Source: National Eye Institute**

wet AMD is with injections in the eye called *“*anti-VEGF” injections. Anti- VEGF injections *reduce* the leakage so that the macula can become dry again.

A submacular haemorrhage is a bleed under the macula that can cause a blood clot. This usually leads to severe and persistent loss of sight in the eye.

**What treatment is this study researching?**

TIGER will compare two treatments:

1. The current standard treatment of anti-VEGF injections
2. The current standard treatment plus surgery

TIGER will find out if surgery plus anti-VEGF injections gives better vision than anti-VEGF injections alone.

**Do I have to take part?**

It is up to you to decide whether to take part. If you decide to take part, you are free to stop participating at any time, without giving a reason.

Your doctor can tell you what other treatments are available outside of this study. The treatment options will depend on the details of your case, and what treatments are available at your hospital.

**What will it happen if I decide to take part?**

- **Consent**

You will be asked to sign a consent form.

- **Screening/baseline visit**

You will have a vision test, eye examination, and photographs of your macula. You will be asked to fill in a questionnaire about your vision.

Not all patients who wish to take part in the study are eligible. Once you have done all the tests above we will tell you if you are eligible and can join the study.

- **Treatment**

The treatment you get will be chosen through a process called “randomisation”, which means, it will be chosen by chance. Half of those joining the study will be randomised to the current standard treatment (anti-VEGF injections), and half will be randomised to the current standard treatment plus surgery.

If you are selected to get the standard current treatment with anti-VEGF eye injections, the first injection may be given to you at the screening/baseline visit, or otherwise within a few days. After that you will get another injection at month 1, another at month 2, and after that, you will get one injection every 2 months until you finish the study. The study will last 12 months. You will get anaesthetic eye drops to numb the eye before each injection. You may feel a slight pricking sensation with the injection.

If you have been selected to get anti-VEGF injections plus surgery, you will have your first anti-VEGF injection at the time of the surgery and then all other injections as explained above. During the surgery we remove the clear gel that fills the inside of the eye and then inject a ‘clot-busting’ drug through the retina and into the macular clot. This drug is called alteplase (or tissue plasminogen activator) and it breaks up the clot of blood you have under your macula. Alteplase is licensed to break up the blood clots that cause heart attack and stroke, but it is not currently licensed to break up clots in the eye. We then fill the inside of your eye with gas to help push the dissolved clot away from the macula.

For most patients a local anaesthetic will be used to numb the eye, and they will be awake during surgery. Alternatively, you can have a general anaesthetic and you will be sleeping during surgery. You could also have local anaesthetic and some sedation, so that you will not feel anxious. Your doctor will discuss these options with you.

Surgery can often be done as day case (patients go home the same day), but some hospitals or patients may prefer an overnight stay in hospital, for example, if you require a general anaesthetic, live far from the hospital, and surgery is late in the day.

If you have been selected to get anti-VEGF injections plus surgery, then after your operation you will need to rest with your head in specific positions during the day for the first five days. You will be given instructions on how to do this (see, Additional patient information: How to position your head after surgery). This is so that the gas bubble, which floats inside your eye, pushes the blood clot away from the macula. You should do this for 50 minutes out of every hour for five days. During the 10-minute breaks you should move around and be active. At night, you should try to sleep on the side of the surgery, with the operated eye lowest. For example, for left eye surgery you should sleep with your left cheek to the pillow. The night time position should be continued for 10 days in total after the operation. You will be given eye drops to reduce pain and the risk of infection, for about a month after surgery.

With the gas in your eye, you should not travel by plane or go up mountains, as at high altitude, the gas bubble will become bigger inside your eye and this will cause increased eye pressure, severe pain and loss of sight.

The gas will disappear from your eye in about 4-8 weeks after surgery (the time varies for each patient) so there is no need to get it removed by another surgery. Your vision will be very blurred until the gas leaves the eye.

If you have cataracts (an opacity of the natural lens in your eye), or are likely to develop cataracts in the near future, your doctor may offer you the option of having your cataract removed at the same surgery. Your doctor will discuss the risks and benefits of cataract surgery with you.

- **Follow up**

If you have surgery, you will return for an eye examination about 1 and 7 days afterwards. Most patients will have surgery only once, but if you had another submacular haemorrhage whilst in the study, and had been allocated to surgery the first time, surgery could be repeated if needed.

Whether you have anti-VEGF injections alone or anti-VEGF injections plus surgery, you will have a study visit at 6 and 12 months to test your vision, examine and photograph your eye, and complete some questionnaires about your vision and wellbeing.

You will also come regularly for ‘standard’ eye checks (these will probably be done even if you were not to join this TIGER study) and to get your anti-VEGF injections; every month initially for three visits and then every two months until you complete the study at month 12. The study staff will ask you about any problems you have had with your eye and general health, and if you needed to attend any other appointments.

**How long does the study last and what happens afterwards?**

The study will last 12 months. After that you will continue your care in the clinic outside the study. Your doctor will discuss this with you.

**What are the risks of anti-VEGF injections?**

The injection may leave a red spot on the white part of your eye, your eye may be a bit red and gritty, and you may have a few small floaters in your vision after the injection. These symptoms usually settle over a few days.

There are potential risks of the anti-VEGF injections. Occasionally, patients get an abrasion on the surface of their eye. This is painful, but gets better over a few days. The pressure in your eye can increase. If you have a cataract it may get worse; if you do not have a cataract you may get one. Cataract is treatable. If you have previously had cataract surgery this risk does not exist.

Rare complications are retinal detachment, where the retina comes off the back of the eye; your sight could be affected as a result, and surgery is usually required to treat it. You may develop an infection or inflammation inside your eye. Inflammation can be successfully treated. Infection can be severe and can cause loss of sight even if it is treated; occasionally it can lead to loss of the eye itself. Serious eye problems, however, occur only very rarely, in fewer than 1 in 900 eye injections.

*If your eye becomes painful or your sight gets worse after an injection then contact your eye doctor immediately,* as you may need to go to the eye clinic urgently. Go to casualty if you are unable to contact your eye doctor.

There are different anti-VEGF injections available, and in this study you will receive one called Eylea. Further information about Eylea is available from the drug manufacturer, at this web address: <https://www.medicines.org.uk/emc/files/pil.2879.pdf>

**What are the risks of the eye surgery?**

You may have pain because of the surgery. This can usually be controlled with painkillers.

You could lose sight despite having the surgery (the surgery may not work). This can also happen if you get the anti-VEGF injections alone.

You might get a cataract as a result of surgery. As noted above, cataract is an opacity of the natural lens in your eye. If you already have a cataract then it could get worse. Cataracts can be successfully treated with eye surgery. You will not get a cataract if you have previously had cataract surgery.

The eye pressure may go low or high after the surgery. Low pressure usually settles with time. High eye pressure can be treated with eye drops, and usually gets better after a few days or weeks.

After surgery, your retina could tear and detach (retinal detachment). Retinal detachment needs another operation to treat it.

Your eye could bleed at the time of the surgery or after the surgery. Bleedings can occur even if you have no treatment.

A small hole in the middle of your macula could form. This is rare. If a macular hole forms, it can be treated with surgery.

You could develop an infection in your eye. This is very rare. As with the infections that can occur after anti-VEGF injections, a bad infection could potentially lead to loss of sight and even your eye.

The drug used to dissolve the blood clot under your macula could increase the risk of bleeding in your eye. Very rarely, patients are allergic to the drug, causing inflammation or damage inside their eye.

Even if surgery and/or anti-VEGF injections improve your vision it will probably not be completely normal, as submacular haemorrhage is a serious eye problem.

The rare complications of surgery and anti-VEGF injections could damage your vision, but the risk of not having any treatment is probably far greater.

**What other risks are there?**

You will have an eye test called angiography, usually at or near the start of the study. Angiography is part of standard care, so you will probably have this test even if you do not join the study. The purpose of this test is to determine the cause of the submacular haemorrhage.

Two coloured dyes are injected into a vein in the arm or hand and they then travel in the blood stream to your eye. Using a special camera, the dye reveals the fine blood vessels in the macula.

The dye turns your skin yellow for a few hours. The dye leaves your body in the urine, which will turn green for up to 24 hours.

Occasionally, people feel sick immediately after the dye injection, or rarely they vomit. The nausea only lasts a few seconds. If the dye leaks out of your vein during the injection, some of the skin around the injection site may feel uncomfortable and be slightly discoloured. The discomfort usually lasts a few minutes, and the discolouration disappears in a few days.

Occasionally people develop an itchy skin rash that needs treatment with antihistamines tablets. The rash tends to settle in a day or two.

A very severe allergic reaction can occur in fewer than one in a million people. This can cause breathing and/or heart problems which can be life-threatening. You will be monitored for this side effects, and treatment will be immediately available in the rare event that it occurs.

The vast majority of patients tolerate angiography well, with no problems.

**What are the possible benefits of taking part?**

Studies suggest that people who take part in clinical trials may tend to do better than those who do not.

Not all eye centres offer surgery for submacular haemorrhage, and some centres do not offer anti-VEGF injections for submacular haemorrhage. If you participate in this study you will receive one or both. It is likely that both these treatments are much better than no treatment.

You will also know that you are helping to advance medical science. The results of this study will help other patients with AMD and submacular haemorrhage in the future.

**Should I continue my blood-thinning medications?**

Blood-thinning medicines are permitted in this study, but if you take drugs such as aspirin, clopidogrel, warfarin, apixaban, rivoroxiban and others, you need to consider if continuing them poses a risk to your eye, versus the risk to other parts of your body that could occur if you stop them. Please discuss this issue with the doctor who prescribed your blood-thinning medicines, your GP and your eye doctor. In general, you should continue any blood-thinning medicines unless advised to stop or alter them.

**What will happen to the results of the research study?**

The results of this study will be published in international medical journals and presented at medical conferences. They will also be presented at patient groups.

**What if I have any questions, concerns or complaints about the study?**

If you have any questions about this study, or concerns about the way in which it is being carried out, please ask to speak to the researchers at your Hospital.

If you are unhappy with the way you have been looked after and wish to complain formally, you can do this through your hospital’s complaints procedure.You can contact the hospital’s Patient Advise and Liaison Service (PALS):

Telephone:

INSERT LOCAL CONTACT DETAILS

Location:

INSERT LOCAL HOSPITAL LOCATION

Web address:

INSERT LOCAL HOSPITAL ADDRESS

The NHS provides a website with information about how clinical trials are designed, and things you might like to know about taking part: [www.nhs.uk/conditions/clinical-trials/pages/introduction.aspx](http://www.nhs.uk/conditions/clinical-trials/pages/introduction.aspx)

**What if something goes wrong?**

If something goes wrong and you are harmed due to someone’s negligence, then you may have grounds for legal action against your hospital, or the study organiser, but you may have to pay for your legal costs. The study organiser has insurance to cover potential legal liability caused by their design of the research.

**What happens if I want to leave the study?**

You can leave the study at any time without giving a reason. If you decide to leave the study, it will not stop you accessing whatever standard clinical care is available or your legal rights. However, you may or may not be able to continue with anti-VEGF treatment as not all hospitals provide this as standard treatment, and your check-ups may be less frequent. You should ask your doctor what treatments are available if you leave the study.

If you want to leave the study early it would help us if you could complete the eye tests that we usually do on the last day of the study, so we have a more complete set of eye measurements, but you do not need to complete this exit visit if you do not want to.

If you leave the study, we will keep and use the data we have collected until the day of your withdrawal.

**Data protection and confidentiality**

**How will we use information about you?**

We will need to use information from you and from your medical records for this research project. This information will include some of your personal details [initials/ NHS number/ name/ contact details].  People will use this information to do the research or to check your records to make sure that the research is being done properly. People who do not need to know who you are will not be able to see your name or contact details. Your data will have a code number instead. We will keep all information about you safe and secure.

Once we have finished the study, we will keep some of the data so we can check the results. We will write our reports in a way that no-one can work out that you took part in the study.

We may make trial data and images available to other researchers, so that they can investigate different clinical questions, but this information will be anonymised so that your personal details are not identifiable.

Data may also be shared with other clinical and scientific staff who help with the study, such as experts in statistics and experts who will analyse the eye images that are collected during the study. Your personal details will be removed prior to their analysis, but your initials and date of birth may need to be checked by these experts to verify the source of the data.

Images of the inside of your eye may be used by the trial team in clinical talks, presentations and medical publications, to illustrate the effect of treatment and AMD. These images will be anonymised, with no personal details provided.

King’s Health Partners Clinical Trials Office staff (<https://khpcto.co.uk/>) will have access to your research and medical records, to monitor the conduct of the study. If you consent to take part we will use your data to deliver this project as described in this Patient Information Sheet.  If you would like to find out more please read the supplementary leaflet provided, entitled **‘How we use your data**?’

Sites to ensure the GDPR supplementary sheet (How We Use Your Data) is attached to the PIS.

We will write to your GP to inform him or her about your involvement in this study.

In agreeing to take part in this study you acknowledge that your medical notes may be looked at by responsible individuals from the study organisers, other researchers involved in this study, and from regulatory authorities where it is relevant to you taking part in research.

**What are your choices about how your information is used?**

- You can stop being part of the study at any time, without giving a reason, but we will keep information about you that we already have.
- If you choose to stop taking part in the study, we would like to continue collecting information about your health from [central NHS records/ your hospital/ your GP]. If you do not want this to happen, tell us and we will stop.
- We need to manage your records in specific ways for the research to be reliable. This means that we won’t be able to let you see or change the data we hold about you.
- If you agree to take part in this study, you will have the option to take part in future research using your data saved from this study.

**Where can you find out more about how your information is used?**

You can find out more about how we use your information

- at [www.hra.nhs.uk/information-about-patients/](https://www.hra.nhs.uk/information-about-patients/)
- Our Data Protection Officer - [kch-tr.information-governance-queries@nhs.net](mailto:kch-tr.information-governance-queries@nhs.net) (INSERT LOCAL CONTACT)
- by asking one of the research team
- by sending an email to TIGERSTUDY - kch-tr.tigerstudy@nhs.net

**Who has reviewed the study?**

The study has been reviewed by the Cambridge East Research Ethics Committee.

**Who is organising and funding this study?**

The organiser of the study (the Sponsor) is King’s College London/King’s College Hospital. The study is funded by Fight for Sight a leading UK charity.

**Further questions?**

Do not hesitate to ask questions if there is anything you do not fully understand, or if you would like more information:

**Principal Investigator: .................................................................**

**Tel: ................................................................................................**

**Research Nurse/Study Coordinator: ......................................**

**Tel: .............................................................................................**

**This information sheet is yours to keep. If you decide to participate in this study, you will be asked to sign a consent form and a copy of the consent form will be provided for your records.**

**Thank you for considering joining this study.**

**This research study is funded by Fight for Sight.
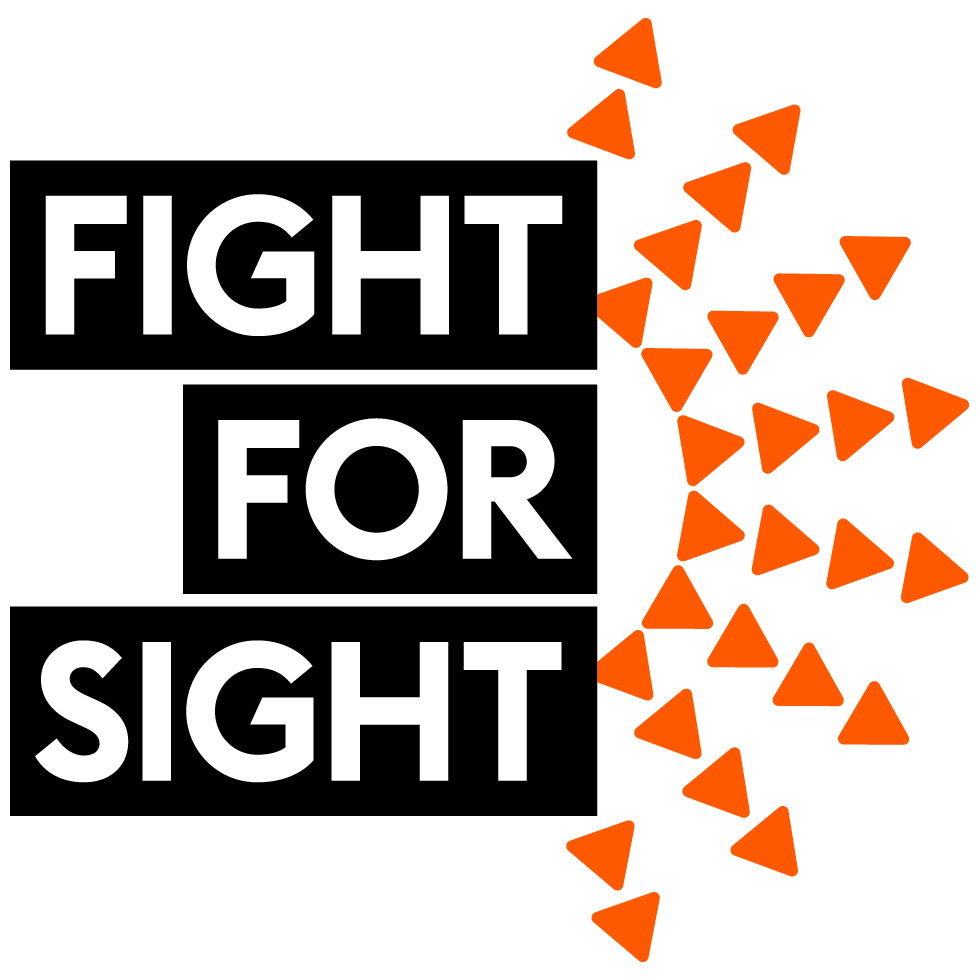
**
