# Supplementary material for: Vitrectomy, subretinal Tissue plasminogen activator and Intravitreal Gas for submacular haemorrhage secondary to Exudative Age-Related macular degeneration (TIGER): study protocol for a phase 3, pan-European, two-group, non-commercial, active-control, observer-masked, superiority, randomised controlled surgical trial
Source: Trials. 2022 Jan 31;23:99. doi: 10.1186/s13063-021-05966-3 (PMC8805308; doi:10.1186/s13063-021-05966-3)
Supplement: Supplementary file 9 — Additional file 9. Appendix 9: Ethical Approval Documentation. [file 13063_2021_5966_MOESM9_ESM.pdf]

Ymchwil Iechyd a Gofal Cymru
Health and Care Research Wales

NHS
Health Research Authority

Timothy Jackson
King's College London
King's College Hospital
Denmark Hill
SE5 9RS

Email: approvals@hra.nhs.uk
HCRW.approvals@wales.nhs.uk

19 February 2021

Dear Jackson

HRA and Health and Care Research Wales (HCRW) Approval Letter

**Study title:**	**Vitrectomy, subretinal Tissue plasminogen activator and Intravitreal Gas for submacular haemorrhage secondary to Exudative age-Related macular degeneration (TIGER): a pan-European, two-group, active-control, observer-masked, superiority, randomised controlled surgical trial.**
**IRAS project ID:**	**276366**
**EudraCT number:**	**2020-004917-10**
**Protocol number:**	**TIGER**
**REC reference:**	**20/EE/0293**
**Sponsor**	**King's College London**

I am pleased to confirm that **HRA and Health and Care Research Wales (HCRW) Approval** has been given for the above referenced study, on the basis described in the application form, protocol, supporting documentation and any clarifications received. You should not expect to receive anything further relating to this application.

Please now work with participating NHS organisations to confirm capacity and capability, in line with the instructions provided in the "Information to support study set up" section towards the end of this letter.

How should I work with participating NHS/HSC organisations in Northern Ireland and Scotland?

HRA and HCRW Approval does not apply to NHS/HSC organisations within Northern Ireland and Scotland.

If you indicated in your IRAS form that you do have participating organisations in either of these devolved administrations, the final document set and the study wide governance report (including this letter) have been sent to the coordinating centre of each participating nation. The relevant national coordinating function/s will contact you as appropriate.

Please see IRAS Help for information on working with NHS/HSC organisations in Northern Ireland and Scotland.

How should I work with participating non-NHS organisations?
HRA and HCRW Approval does not apply to non-NHS organisations. You should work with your non-NHS organisations to obtain local agreement in accordance with their procedures.

What are my notification responsibilities during the study?

The standard conditions document "*After Ethical Review – guidance for sponsors and investigators*", issued with your REC favourable opinion, gives detailed guidance on reporting expectations for studies, including:

- Registration of research
- Notifying amendments
- Notifying the end of the study

The HRA website also provides guidance on these topics, and is updated in the light of changes in reporting expectations or procedures.

Who should I contact for further information?
Please do not hesitate to contact me for assistance with this application. My contact details are below.

Your IRAS project ID is **276366**. Please quote this on all correspondence.

Yours sincerely,
Laura Greenfield

Approvals Specialist

Email: approvals@hra.nhs.uk

Copy to: *Ms Amy Holton*

List of Documents

The final document set assessed and approved by HRA and HCRW Approval is listed below.

Document	*Version*	*Date*
Confirmation of Clinical Trial Authorisation from MHRA and relevant correspondence		16 February 2021
Contract/Study Agreement template		
Copies of materials calling attention of potential participants to the research [Poster for patients]	1.1	03 January 2021
Copies of materials calling attention of potential participants to the research [Poster for clinic]	1.1	03 January 2021
Covering letter on headed paper [HRA Response Cover letter]		19 January 2021
Covering letter on headed paper [HRA Response Cover letter]		17 February 2021
Evidence of Sponsor insurance or indemnity (non NHS Sponsors only) [Insurance]		04 August 2020
GP/consultant information sheets or letters [GP letter]	1.1	03 January 2021
IRAS Application Form [IRAS_Form_19012021]		19 January 2021
IRAS Checklist XML [Checklist_17022021]		17 February 2021
Letter from funder [Funder letter]		07 September 2020
Letter from funder		
Letters of invitation to participant [Pre-invite letter]	1	12 November 2020
Non-validated questionnaire [Health Resource use Questionnaire]	1.0	05 January 2021
Organisation Information Document		
Other [Pocket guide]	1.3	19 January 2021
Other [Bayer letter of support]		28 August 2019
Other [PPI minutes]		09 October 2018
Other [Dec of Conformity Cannula]		30 January 2020
Other [CE status of Cannula]		29 December 2020
Other [MedOne CE Certificate 2020]		16 December 2019
Other [Instructions for Posturing]	1.0	30 December 2020
Other [GCP of Chief Investigator]		29 November 2020
Participant consent form [Consent Form]	1.2	15 February 2021
Participant information sheet (PIS) [PIS]	1.2	15 February 2021
Participant information sheet (PIS) [GDPR statement]	1	21 November 2019
Referee's report or other scientific critique report [Scientific Review]		
Research protocol or project proposal [Protocol]	1.3	19 January 2021
Sample diary card/patient card [Patient ID card]	1	12 November 2020
Schedule of Events or SoECAT [SoE]	12/11/2020	
Summary CV for Chief Investigator (CI) [CV for Chief Investigator]		27 November 2020
Summary of product characteristics (SmPC) [SmPC]		11 June 2019
Validated questionnaire [NEI VFQ25 - Validated questionnaire]		
Validated questionnaire [EQ-5D Questionnaire]		
Validated questionnaire [SWEMWBS Questionnaire]		

IRAS project ID	276366

Information to support study set up

The below provides all parties with information to support the arranging and confirming of capacity and capability with participating NHS organisations in England and Wales. This is intended to be an accurate reflection of the study at the time of issue of this letter.

Types of participating NHS organisation	Expectations related to confirmation of capacity and capability	Agreement to be used	Funding arrangements	Oversight expectations	HR Good Practice Resource Pack expectations
This is a multicentre non-commercial CTIMP study where the research activities undertaken at each site use the same protocol	Research activities should not commence at participating NHS organisations in England or Wales prior to their formal confirmation of capacity and capability to deliver the study	An Organisation Information Document has been submitted and the sponsor is intending to use a separate site agreement. The agreement is unmodified.	Funding arrangements are located in the submitted site agreement	A Principal Investigator is expected to be in place at the participating NHS site	All research activities at sites will be undertaken by local staff, it is therefore unlikely that additional arrangements (letters of access or honorary research contracts) will be applicable, except where individuals employed by another Trust or University (e.g. local network staff) are involved, and arrangements are not already in place.<br>Where no prior arrangements are in place, network staff (or similar) undertaking any of the research activities listed in IRAS form A18 or A19 (except for administration of questionnaires or surveys), would be expected to obtain an honorary research contract from one NHS organisation (if University

					employed), followed by Letters of Access for subsequent organisations. This would be on the basis of a Research Passport (if University employed) or an NHS to NHS confirmation of pre-engagement checks letter (if NHS employed). These should confirm enhanced DBS checks, including appropriate barred list checks, and occupational health clearance. For research team members administering questionnaires or surveys only, a Letter of Access based on standard DBS checks and occupational health clearance would be appropriate.

Other information to aid study set-up and delivery

This details any other information that may be helpful to sponsors and participating NHS organisations in England and Wales in study set-up.
The applicant has indicated that they intend to apply for inclusion on the NIHR CRN Portfolio.
