# Supplementary material for: Vitrectomy, subretinal Tissue plasminogen activator and Intravitreal Gas for submacular haemorrhage secondary to Exudative Age-Related macular degeneration (TIGER): study protocol for a phase 3, pan-European, two-group, non-commercial, active-control, observer-masked, superiority, randomised controlled surgical trial
Source: Trials. 2022 Jan 31;23:99. doi: 10.1186/s13063-021-05966-3 (PMC8805308; doi:10.1186/s13063-021-05966-3)
Supplement: Supplementary file 10 — Additional file 10. Appendix 10: Funding Letter of Support. [file 13063_2021_5966_MOESM10_ESM.pdf]

DocuSign Envelope ID: 788B065E-56B7-401E-9727-A5723741E82C

Professor Timothy Jackson
King's College London
School of Life Sciences and Medicine
Department of Ophthalmology
King's College Hospital
London SE5 9RS

11 August 2020

Dear Professor Jackson

Sub-Macular Haemorrhage Clinical Research Grant– Grant Ref: 2036 / 2037

I am pleased to inform you that the Trustees of Fight for Sight have chosen to award King's College London (KCL) (the "Host Institution") a Grant (the "Grant") for the following Clinical Trial Award on the terms of this letter and the enclosed terms and conditions (the "Grant Terms"). The words and phrases used in this letter shall, unless the context requires otherwise, have the same meaning as the defined words and phrases in the Grant Terms.

Terms and Conditions:
The Grant is awarded for the research outlined in your application of November 2018 entitled ***"Vitrectomy, subretinal Tissue plasminogen activator and Intravitreal Gas for submacular haemorrhage secondary to Exudative age-Related macular degeneration (TIGER): a European randomised controlled surgical trial".*** The agreed start date is September 2020. Any changes to this start date must be agreed in writing with Fight for Sight.

18 Mansell Street London E1 8AA
T 020 7264 3907 E Grants@fightforsight.org.uk
fightforsight.org.uk

British Eye Research Foundation operating as Fight for Sight is a company limited by guarantee registered in England (Company Number: 05525503) and a charity registered in England by the Charity Commission for England and Wales (Registered Charity Number: 1111438) whose registered office is at 18 Mansell Street, London E1 8AA

Please note the following amendments to the terms and conditions which this award letter supersedes:

- The Award Holder, Professor Tim Jackson, will retain full responsibility of the grant, which will move with the Award Holder in the event of a change in the Host Institution.
- The total amount of the Grant will be £1,799,779, paid in tranches over **60** months as in Table 1 below.

Finances:

Table 1: Breakdown of Payments

Item	Year 1* (inc. setup)	Year 2	Year 3	Year 4	Year 5	Total
Salary – Senior Trial Manager (King's College Hospital) 40%WTE	£26,267	£26,864	£27,134	£27,711	£29,056	£137,032
Salary- Clinical Research Fellow (King's College Hospital)	£15,463	£15,619	£15,774	£15,932	£16,091	£78,879
Salary- Trial Manager (King's College London) 50% WTE	£22,449	£23,944	£25,537	£27,235	£29,047	£128,212
Salary- Barnaby Reeves (University of Bristol)	£7,021	£0	£0	£0	£0	£7,021
Salary – Statistician (King's College London)	£9,478	£10,160	£10,996	£11,732	£12,516	£54,882
Other costs including Conferences and travel	£21,064	£20,412	£20,412	£20,412	£16,012	£98,312
Site payments	£78,000	£75,000	£75,000	£75,000	£75,000	£378,000
Equipment including Computers	£5,069	£3,738	£0	£0	£0	£8,807
Publication / Dissemination costs	£1,500	£0	£0	£0	£2,881	£4,381
Randomisation and database	£19,497	£11,152	£11,152	£10,000	£10,000	£61,801
External subcontractors/consultants providing services	£116,894	£50,163	£45,625	£45,625	£38,750	£297,057
Consumables	£960	£960	£960	£960	£960	£4,800
Research facilities- KHPCTO	£108,119	£108,119	£108,119	£108,119	£108,119	£540,595
**Total Support Awarded**	**£431,781**	**£346,131**	**£340,709**	**£342,726**	**£338,432**	**£1,799,779**

**This letter (dated 11 August 2020) releases Year one payment of the full award.*

The Grant will be paid in instalments by cheque or electronic payment. Claims for reimbursement should be submitted quarterly in arrears, within one month of the quarter end, by the Host Institution's Finance Office. Each claim should include a detailed breakdown of the expenditure incurred.

DocuSign Envelope ID: 788B065E-56B7-401E-9727-A5723741E82C

The award of this grant is conditional on funding being received from the Foundation of the Society of Retina Specialists (Foundation). Fight for Sight will not underwrite the full term of the grant and only commits to funding the grant on an annual basis subject to sufficient payments being received from the Foundation.

Release of each annual tranche of funding will be dependent on satisfactory progress of the trial. Trial progress will be assessed by the Research, Policy and Innovation Committee which will decide whether the next grant award letter should be released. Points that will be considered include, submission and approval of relevant ethics and regulatory applications, site set-up and patient recruitment (including first and last patient recruited). Release of the final grant payment will be dependent on receipt of a Final Report. Invoices and the supporting breakdown of expenditure should be emailed to **invoices@fightforsight.org.uk**. Any unspent funds should be returned to Fight for Sight at the conclusion of the grant. The Award Holder must obtain the written consent and approval from Fight for Sight before repurposing any funds.

Reporting and Monitoring:
One Fight for Sight staff member should be included on the Trial Steering Committee as an observer and funder representative. Fight for Sight will monitor the progress of this award for its full duration, including any agreed extension periods. Monitoring includes, but is not restricted to, quarterly updates on patient recruitment and trial progress, annual submission of a pro-forma report, and submission of an end of grant Final Report. Any significant changes to the study design (for example, but not limited to, the addition of removal of a study arm, a change to the study drug, a change to the sample size) can only be made with prior agreement from Fight for Sight. You are advised to contact us at the earliest possible opportunity to discuss any such changes.

Under no conditions shall Fight for Sight or the Foundation hold any of the responsibilities or obligations of a trial “sponsor” (as defined in any of the relevant legislation or guidelines, including the EU Clinical Trials Directive 2001/20/EC, the pending EU Regulation 536/2014, their equivalent national legislation in any jurisdiction, or in ICH-GCP). The legal obligations of a trial “sponsor” shall sit with the Host Institution or any other third party it has identified for this purpose.

Clinical studies funded by Fight for Sight qualify for adoption into the UK Clinical Research Network (UKCRN) Portfolio and are consequently eligible for consideration for research infrastructure and service support from the NIHR Clinical Research Network. We expect you to keep your study record on the Portfolio Database up to date and upload your recruitment data on a monthly basis. The Portfolio Database is also used to monitor the progress of clinical studies by regularly recording recruitment data. The Grant holder must ensure that up-to-date trial information, including recruitment data, is submitted monthly through the designated accrual data contact.

The Grant holder must register the trial on a recognised trials registry such as the ISRCTN registry, the EU Clinical Trials Register (EudraCT) or the ClinicalTrials.gov register before the first patient is recruited. Grant holders are required to make

DocuSign Envelope ID: 788B065E-56B7-401E-9727-A5723741E82C

summary results (whether positive or negative) of their trial publicly available, without unreasonable delay, and generally within 12 months of the end of trial (unless there is a scientifically justified longer time period). The results should be posted on the same registry as the trial was listed and the trials registry identifier should be used in publications to ensure the results are discoverable.

Please ensure that the Fight for Sight logo is included on Patient Information Sheets.

Intellectual Property:
The Host Institution shall carry out the Work in accordance with accepted scientific and/or academic principles and standards and shall endeavour to ensure the accuracy of the results of the work. However, the Parties recognise that this Grant provides for the carrying out of experimental research and that Arising IP or Background IP may contain experimental materials, data or processes whose properties and safety may not have been established. Therefore the Host Institution does not undertake that the work will provide specific results or provide data which can be used for a particular purpose, and any Arising IP, Background IP, information or materials which the Host Institution provides under this Agreement are supplied 'as is' and without any express or implied warranties, representations or undertakings.

The Host Institution shall not be liable for the consequences or effects of any use to which Fight for Sight or any third party may put Arising IP, except to the extent that such claims arise from the proven negligence or default of the Host Institution or its employees, students, representatives, consultants or agents. Fight for Sight shall fully and effectively indemnify, defend and hold harmless the Host Institution and its employees, students, directors, consultants, representatives and agents against all legal actions arising from the use which it puts, or permits to be put, Arising IP.

Dispute Resolution:
If any dispute arises out of this Agreement the Parties will first attempt to resolve the matter informally through designated senior representatives of each Party to the dispute, who are not otherwise involved with the Project. If the Parties are not able to resolve the dispute informally within a reasonable time not exceeding two (2) months from the date the informal process is requested by notice in writing they will attempt to settle it by mediation in accordance with the Centre for Effective Dispute Resolution (CEDR) Model Mediation Procedure.

Promotion and Impact:
Fight for Sight depends entirely on voluntary income to fund research and it's really important that we are able to maximise publicity to raise awareness of your research and fundraise for the work that we do. By signing the terms and conditions you are agreeing to acknowledge us in any reports or publications and to involve us in any publicity. You are also agreeing for Fight for Sight to have input and sign-off on any press releases or other activity promoting the outcomes of your work. Finally, you are agreeing that we may lead our own promotion of your research, working with you on any press releases, statements and interviews, and respecting any embargoes around research findings. Please do keep us updated on any milestones, developments or publications associated with your project by emailing the RPI team

DocuSign Envelope ID: 788B065E-56B7-401E-9727-A5723741E82C

at grants@fightforsight.org.uk. We are always keen to work with you to promote your work and raise awareness of the need for vital vision research.

Additionally, our press team can work with the communication office at the Host Institution but requests that they be the first point of contact before any research is published so as to maximise the opportunity for promotion of your work. They should be contacted well in advance at Press@fightforsight.org.uk.

We may also ask for your help in talking to local groups to support fundraising for Fight for Sight. By accepting the grant you are agreeing to respond to requests that concern your grant and to help to support Fight for Sight in its fundraising efforts. We would be extremely grateful for your support in this area and a member of Fight for Sight staff may be in touch to discuss the promotion of your research.

Please acknowledge acceptance of the Grant by an authorised signatory at the Host Institution signing below and returning one copy to Steven Smith, Grants Manager, at Fight for Sight, 18 Mansell Street, London E1 8AA.

Kind regards

Yours sincerely

Sherine Krause

Sherine Krause
Chief Executive

Host Institution Signatory Details:

**Signed**	DocuSigned by: Daniela Amadio EE3528425582473...
**Name in full**	Daniela Amadio
**Position**	Head of Research Grants
**Date**	07/09/2020
